# Metabolic connectivity has greater predictive utility for age and cognition than functional connectivity

**DOI:** 10.1093/braincomms/fcaf075

**Published:** 2025-02-18

**Authors:** Hamish A Deery, Emma X Liang, Chris Moran, Gary F Egan, Sharna D Jamadar

**Affiliations:** School of Psychological Sciences, Monash University, Melbourne 3800, Australia; Monash Biomedical Imaging, Monash University, Melbourne 3800, Australia; School of Psychological Sciences, Monash University, Melbourne 3800, Australia; Monash Biomedical Imaging, Monash University, Melbourne 3800, Australia; School of Public Health and Preventive Medicine, Monash University, Melbourne 3004, Australia; Monash Biomedical Imaging, Monash University, Melbourne 3800, Australia; School of Psychological Sciences, Monash University, Melbourne 3800, Australia; Monash Biomedical Imaging, Monash University, Melbourne 3800, Australia

**Keywords:** functional PET, fMRI, functional connectivity, ageing, cognition

## Abstract

Recently developed high temporal resolution functional (18F)-fluorodeoxyglucose positron emission tomography (fPET) offers promise as a method for indexing the dynamic metabolic state of the brain *in vivo* by directly measuring a time series of metabolism at the post-synaptic neuron. This is distinct from functional magnetic resonance imaging (fMRI) that reflects a combination of metabolic, haemodynamic and vascular components of neuronal activity. The value of using fPET to understand healthy brain ageing and cognition over fMRI is currently unclear. Here, we use simultaneous fPET/fMRI to compare metabolic and functional connectivity and test their predictive ability for ageing and cognition. Whole-brain fPET connectomes showed moderate topological similarities to fMRI connectomes in a cross-sectional comparison of 40 younger (mean age 27.9 years; range 20–42) and 46 older (mean 75.8; 60–89) adults. There were more age-related within- and between-network connectivity and graph metric differences in fPET than fMRI. fPET was also associated with performance in more cognitive domains than fMRI. These results suggest that ageing is associated with a reconfiguration of metabolic connectivity that differs from haemodynamic alterations. We conclude that metabolic connectivity has greater predictive utility for age and cognition than functional connectivity and that measuring glucodynamic changes has promise as a biomarker for age-related cognitive decline.

## Teaser

Deery *et al*. report that there is a reconfiguration of metabolic brain networks in ageing that is different to haemodynamic alterations and is more strongly linked to cognitive performance. They conclude that metabolic connectivity offers utility to understand brain network changes in health and disease.

## Graphical Abstract

**Graphical Abstract:**
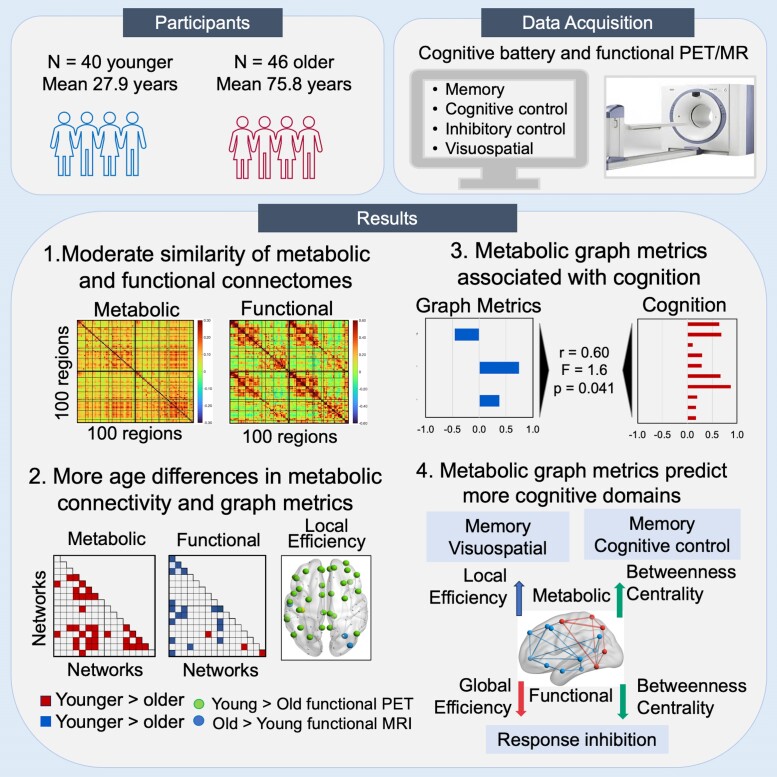

## Introduction

The history of neuroscientific inquiry into brain function that spans several centuries has vastly expanded our understanding of behaviour and cognition.^1^ One important area of study has been brain energetics because of the obligatory role that metabolism plays in brain function.^2^ A range of experimental methods and tools have been used to study metabolic pathways in the brain and have revealed a tight coupling between neuronal activity and metabolism at multiple temporal and spatial scales.^3-5^ Increases in the brain’s utilization of glucose matches synaptic activity,^4,6-9^ and at the cellular level, neuronal glucose oxidation is directly related to the frequency of spike activity and neurotransmitter cycling rates.^10,11^ Functional-metabolic associations are also evident at a regional level.^2,12^ Early studies using (18F)-fluorodeoxyglucose positron emission tomography (FDG-PET) compared images from rest and task conditions and showed an increase in glucose metabolism in task-relevant visual^13^ and motor^14^ regions.

The oxidation of glucose in the brain in response to neuronal activity is also accompanied by an increase in local oxygen consumption and cerebral blood flow, leading to the increase in oxygenation that produces the blood oxygenated level dependent (BOLD) signal that underpins fMRI.^15,16^ fMRI has provided ground-breaking knowledge about the functional architecture of the brain, and shown that cognitive functions are dependent on coherent activity between brain regions in large-scale networks.^17,18^ However, fMRI provides only one index of the coherent neurophysiological signals that underlie the brain’s functional network. Importantly, information transfer across the brain arises from multiple physiological processes, including haemodynamic, electrophysiological and molecular processes. Molecular imaging techniques, including high temporal resolution functional FDG-PET (fPET), allow for the dynamic within-subject measurement of molecular processes, such as glucose metabolism.^19-28^ The coherence of glucose signals across the brain indexed with FDG is termed ‘metabolic connectivity’.^29,30^ We have reported that metabolic connectivity provides important and complementary insights into brain function relative to BOLD-fMRI derived networks.^20,21,29^

Given the large individual and societal costs of age-related cognitive decline, brain changes in normative ageing and neurodegenerative conditions are a crucial focus of neuroscientific enquiry.^31^ fMRI has revealed that large-scale functional brain networks in ageing are characterized by a reduction of within-network connectivity, an increase in between-network connectivity and a reduction in the efficiency of connections across the brain, particularly connections between associative networks.^32^ Mechanistically, neuroimaging studies utilizing the BOLD signal and glucose metabolism have shared but important unique components that have implications for their interpretation.^30^ The BOLD signal reflects changes in deoxyhaemoglobin driven by localized changes in blood flow and blood oxygenation. However, the interpretation of altered BOLD signals between groups can be challenging, as any alteration in the coupling in cerebral blood flow, volume or oxygen metabolism, unrelated to changes in neuronal activity, will result in an altered BOLD signal.^16,33,34^ For example, the coupling between blood flow, blood volume and metabolism are differentially altered in healthy ageing and hence age-related changes of the BOLD signal may reflect alterations in one or more neurovascular component in older adults.^9,35-37^

In contrast, FDG-PET signals in the brain are proportional to glucose metabolism,^38^ and can therefore provide a complementary, more direct and quantifiable measure of neuronal activity than the BOLD signal. Whole-brain simultaneous fPET/fMRI in younger adults has revealed that metabolic connections are predominantly between frontal and parietal regions, whereas fMRI connections are more widespread across the brain.^29,39,40^ Moreover, fPET and fMRI connectomes in younger adults are differentially related to executive function.^24^ Using fPET, we recently showed differences in the metabolic connectomes of older and younger adults.^41^ We found lower metabolic connectivity in older than younger adults between the frontal and temporal regions, and more globally integrated posterior hub regions in older adults. Qualitatively, these results contrast with the fMRI literature, which shows increased connectivity between the frontal and temporal regions and a posterior to anterior shift in ageing.^32^

Here, we use simultaneous fPET/fMRI to compare metabolic and functional connectivity and test their predictive utility for healthy ageing and cognition. Given that the FDG and BOLD signals index some shared physiological processes, we hypothesize that there will be moderate similarity in the topology of fPET and fMRI connectomes.^29^ Secondly, following our previous qualitative observations of fPET and fMRI differences,^32,41^ we hypothesize that differences between younger and older adults in network connectivity will be more widespread in fMRI than fPET, with lower within- and higher between-network connectivity in older compared to younger adults, particularly for associative networks. Thirdly, that fPET connectomes will show lower local and global efficiency for older than younger adults in line with large-scale functional network alterations reported in fMRI studies.^32^ Finally, we hypothesize that fPET and fMRI graph metrics will both predict cognitive performance, and that the metrics will differentially associate with cognition across domains, in line with our previous work in younger adults.^24^

## Materials and methods

### Ethical considerations

The study methods were reviewed and approved by the Monash University Human Research Ethics Committee. The review ensured the study protocol adhered to the Australian National Statement of Ethical Conduct in Human Research (2023). The Monash Health Principal Medical Physicist approved the administration of ionising radiation in accordance with the Australian Radiation Protection and Nuclear Safety Agency Code of Practice (2005). The effective dose of radiation in this study was 4.8 mSv, below the exposure limit of 5mSv annually for adults in Australia.

### Participants

Ninety participants were recruited from the local community. An initial screening interview was undertaken to ensure participants did not have non-MR compatible implants and a clinical or research PET scan in the previous 12 months. Other exclusion criteria were a history of diabetes, neurological or psychiatric illness and psychoactive medication use. Women were screened for known or suspected pregnancy. Participants received a $100 gift voucher. Four participants were excluded from the analyses because of excessive head motion (*N* = 2) or incomplete PET data (*N* = 2).

#### Participant consent

Participants provided informed consent to participate in the study.

### Data acquisition

#### Demographic and cognitive variables

Participants completed an online demographic and lifestyle questionnaire and a battery of cognitive tests. We assessed performance in cognitive domains that show age-related changes.^42^ The domains included memory, cognitive control, inhibitory control and processing speed, tested as follows.

##### Hopkins verbal learning test

The Hopkins verbal learning test (HVLT) is a test of episodic memory.^43^ Participants were presented with 12 words in three learning trials. The 12 words included four words from each of three semantic categories. A free-recall and recognition test was undertaken 20–25 min after the learning trials. The free-recall tested was of any words remembered. In the recognition trial, participants were presented with 24 words. The 24 words comprised 12 target words from the original learning list and 12 false-positives words. Six of the false-positive words were semantically related to one of the learning list categories and six were semantically unrelated. Delayed recall (total words recalled) and a recognition discrimination index (number of correct minus number of false positives in the recognition task) were recorder to index episodic memory performance.

##### Digit span

The digit span task is a verbal short term and working memory test.^44^ Participants were presented with a series of digits. They were asked to repeat the digits in the order presented in the forward span trials and in the reverse order in the backwards span trials. The number of digits increased by one following a successful trial and the test was stopped after two consecutive failures of the same length. Working memory was indexed as the length of longest correct forward and backward digits recalled.

##### Task switching

Task switching is a test of cognitive control. Participants undertook the test on a computer. They were required to perform a categorization task based on a cue and a word presented on screen. In one series of tasks, a heart cue was presented together with a word, and participants were instructed to indicate if the word represented a living or a non-living object via a key press. In the second series of tasks, an arrow-cross cue was presented and participants were required to categorise a word as representing an object that is bigger or smaller than a basketball. The cue selection for the trials was randomized. Half the test trials were switch trials and half non-switch trials. Half of each type of trial was also congruent in the key presses and half were incongruent. Cognitive control was measured as the percentage of correct switch trials and the mean latency of correctly responding to a switch trial.^45^

##### Stop signal

The stop signal task is a computer-based test of response inhibition. Participants were shown an arrow on screen that pointed to the right or left.^46^ If the arrow pointed to the left, participants were instructed to press the left response key; if the arrow pointed to the right, they were required to press the right response key. If a signal beep was sounded, participants were required to stop their key press response. The delay from the arrow presentation to a stop signal beep commenced at 250 ms and was adjusted up or down depending on performance. The delay increased by 50 ms up to 1150 ms if the previous signal stop was successful and decreased by 50 ms if the previous stop signal was unsuccessful. Response inhibition was indexed from the stop signal trials as the percentage of correct trials and the mean reaction time.

##### Digit symbol substitution

Digit symbol substitution is a test of visuospatial processing in which participant are presented with an 18 columns × 16 rows matrix.^47^ The task required participants to translate symbols shown in a key at the top of the computer screen into digits and type them into the matrix. Visuospatial performance was recorded as the total count of correct responses across the 2 min trial. The latency (seconds) per correct response were also recorded.

##### Neuroimaging acquisition

Participants underwent a 90-minute simultaneous MR-PET scan in a Siemens (Erlangen) Biograph 3-Tesla molecular MR scanner. Half of the 260 MBq FDG tracer was administered at the beginning of the scan as a 130 MBq bolus. The other half of the FDG dose was infused at a rate of 36 ml/h over 50 min.^48^ During the first 12 min, a T1 3D MPRAGE and T2 FLAIR scan was acquired. At 13 min, list-mode PET and T2* EPI BOLD-EPI sequences began. A 40-min resting-state scan was undertaken while participants viewed a video of a drone flying over the Hawaii islands. Full details of the acquisition parameters are provided in the Supplement.

#### MRI and PET pre-processing

Freesurfer was used to extract the T1 images. Quality of the pial and white matter surface was manually checked and corrected where needed. The images were registered to MNI152 space using Advanced Normalization Tools (ANTs). For the BOLD-fMRI data, T2* images underwent brain extracted, unwarping and motion correction. Motion was corrected using six rotation and translation parameters in FSL MCFLIRT. The images were also temporally detrended, normalized to MNI space and smoothed at 8 mm FWHM.^49^ Framewise displacement was calculated for each participant. Excessive head motion was indexed as a mean framewise displacement above 0.3 mm.

The list-mode PET data were binned into 344 3D sinogram frames of 16 s intervals. The PET data were reconstruct with attenuation correction^50^ using the pseudo-CT method for hybrid PET-MR scanners, ordinary Poisson-ordered subset expectation maximization algorithm (3 iterations and 21 subsets) with point spread function. For each volume, the reconstructed DICOM slices were converted to NIFTI format with a size of 344 × 344 × 127 (1.39 × 1.39 × 2.03 mm^3^). The 3D data were concatenated to form a 4D NIFTI volume. After concatenation, the PET volumes were motion corrected using FSL MCFLIRT.^49^ The mean PET image was used to mask the 4D data. Partial volume effects in the PET images were corrected using the modified Müller–Gartner method^51^ in Petsurf.

**Figure 1:**
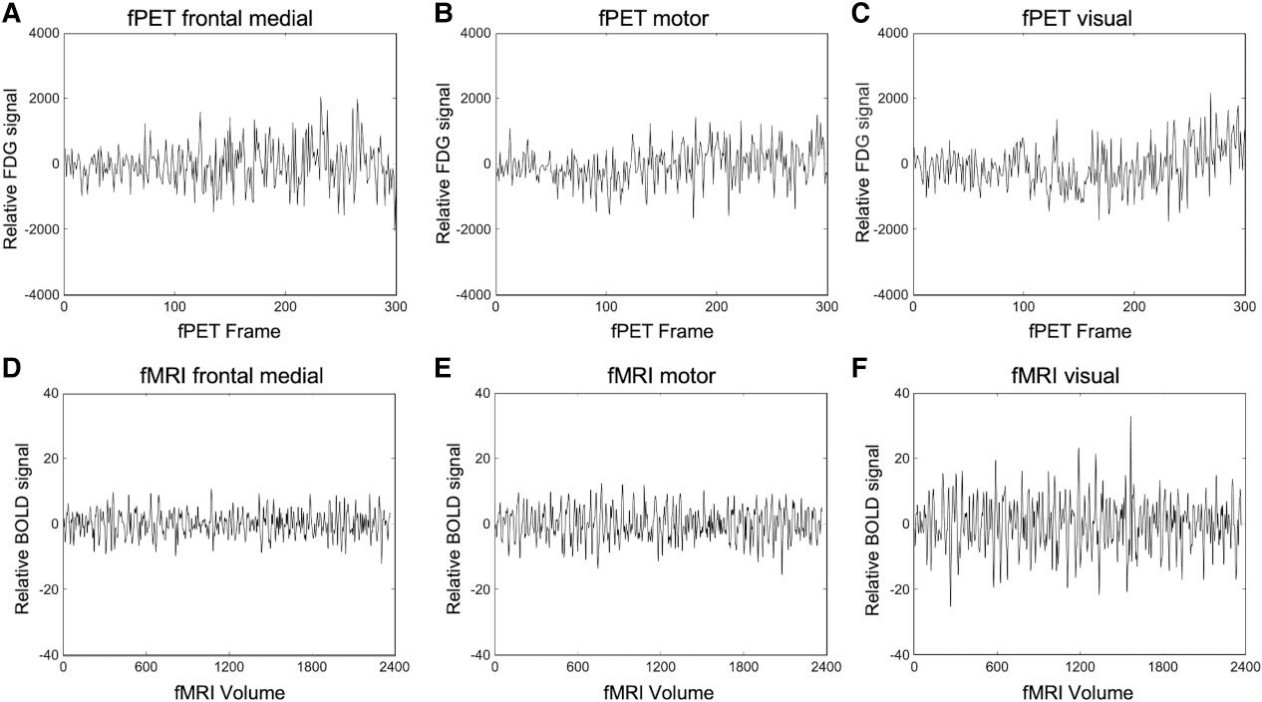
**fPET and fMRI regional time series.** Time series for three regions from a single participant after denoising in the *CONN* toolbox (https://web.conn-toolbox.org/home), including regressing out the white matter and cerebrospinal fluid signal. CONN scales the FDG and BOLD signal at each voxel to the global mean, i.e. the signal average is removed during denoising as a constant term in the regression equation. Hence the time series represents the relative FDG (becquerels) and BOLD signal (arbitrary units). The fPET time series (**A–C**) was analysed from the 10 min point of the scan to minimize the impact of the initial FDG uptake from the bolus and to reflect the point of a stable signal (i.e. frame zero in the plots A to C correspond to the 10 min point of the scan). The fPET framerate (**A–C**) was 16 s and the MRI repetition rate (**D–F**) was one second. Note that connectivity analyses in CONN is scale-invariant.

The *CONN* toolbox in Matlab^52^ was used to analyse participants’ fPET and fMRI data. The T1 images and pre-processed fMRI and fPET time series data for each participant was loaded to CONN. As grey matter uptake of FDG is relatively uniform across the brain, regions are likely to show some degree of connectivity, particularly if the initial steep rise of the tracer uptake is included in the time series. To minimize this issue, the first 10 min of the time series was excluded to remove the initial uptake and to reflect the time corresponding to the onset of a stable signal.^21^ The time series data were also denoised using the default CONN pipeline by regressing out white matter and CSF confounds and scaling to the global mean at each voxel, which further reduced any residual baseline uptake. High frequency noise in the PET signal was filtered using a low pass filter (0.0625 Hz).^25^ A bandpass frequency filtered of 0.01 to 0.1 Hz was used for the fMRI data. Regions of interest were generated for the time series data using the Schaefer atlas.^53^ The resulting fPET and fMRI time series represent the relative FDG and BOLD signal changes across frames and volumes, respectively, from which connectivity analyses was calculated (see Fig. 1).

It is currently unclear if fMRI-derived ‘functional’ atlases are transferable to metabolic connectomes.^19,28,29,40^ Hence, we repeated the analyses using an anatomical parcellation of the data based on the 106 cortical and subcortical regions of the Harvard Oxford atlas (see Supplementary Information, Section 3: Figs. S1–S6 and Tables S7–S12).

Graph theory metrics were calculated in CONN. Metrics were calculated at the top 30% of relative connection strength, a threshold at which age group differences in fPET can be reliably detected.^41^ Global efficiency, local efficiency, betweenness centrality and degree for each node and at the whole brain level were used as they have predictive utility for age in fMRI.^32^ They are defined as:

‘Global efficiency’ at each node is the average of the shortest inverse-distances between the node and all other nodes in the graph. Global efficiency is a measure of global integration across the entire graph.

‘Local efficiency’ at each node is the average of shortest inverse-distances between the node and its neighbouring node in the local sub-graph. Network local efficiency is a measure of local integration.

‘Betweenness centrality’ is the proportion of time that a node is part of a shortest-path between any two nodes of a graph. It is a measure of node centrality of the graph.

‘Degree’ at each node is the number of edges from and to that node. Degree represents the connectedness of each region of the network.

The graph metrics were also calculated at the Schaefer network level for the fPET and fMRI data. The regions within the networks differ in cortical volume. Hence, the regional values could not simply be averaged to calculate the network graph metric values. Instead, each region was weighted by the percentage its volume represented in the total volume of network.

#### Similarity of fPET and fMRI connectomes in the whole sample

To test the first hypothesis that there will be moderate similarity in the topology of fPET and fMRI connectomes, we qualitatively assessed the fPET and fMRI connectomes in the whole sample. We also calculated the Dice–Sørensen coefficient (DICE coefficient) between the connectomes at costs from 10% to 90% of connections in 10% increments to quantify their spatial similarity. To further explore the degree of similarity between the fPET and fMRI connectomes, we calculated the covariance between them, by calculating the Pearson’s correlation of each connection in the matrices across participants.

### Statistical analyses

A series of *t*-tests was used to test for age group differences in connectivity and graph metrics. A false-discovery rate of *P* = 0.05 was used to correct each series of analyses for multiple comparisons. The relative predictive strength of fPET and fMRI graph metrics for age group was assessed using discriminant function analysis. The relationship between the graph metrics and cognition was assessed using canonical correlation and multiple regression analyses.

#### Metabolic connectomes

We qualitatively evaluated the connectomes of older and younger adults across modalities to test the hypothesis that age differences in network connectivity will be more widespread in fMRI than fPET. The connectomes were generated after reverse transforming the Fisher z-values back to r-values for each participant. The average connectivity at each node was calculated separately for the younger and older adult groups for both the fPET and fMRI data. The Fisher transformed values were used for all other connectivity and graph metric analyses comparing younger and older adults.

#### Within- and between-network connectivity

Within- and between-network connectivity was calculated for the younger and older adult groups. ‘Within-network connectivity’ was calculated by averaging the Fisher transformed z-value of all regions within the network. ‘Between-network connectivity’ was calculated by averaging the regional connections within that network and the regions in each other network. To test the hypothesis that older adults will have lower within- and higher between-network connectivity than younger adults, independent sample *t*-tests were used and tested for significance at *P*-FDR < 0.05, one tailed.

#### Age differences in fPET and fMRI graph metrics

A series of *t*-tests was used to test the third hypothesis that the graph metrics derived from the fPET and fMRI connectomes will show lower global and local efficiency and centrality and degree in older than younger adults. First, we compared younger and older adults on the graph metrics at the region level. Second, we compared younger and older adults on the graph metrics at the network level. Each series of *t*-tests for a graph metric was tested for significance at *P*-FDR < 0.05, one tailed.

#### Relative predictive strength of fPET and fMRI graph metrics for age group

Discriminant function analysis was used to classify participants into their predefined age groups based on the fPET and fMRI whole brain graph metrics separately (degree was not included, as the graphs were thresholded at the top 30% of edges for all participants, resulting in the same degree at the whole brain level). Wilks’ Lambda was used to test the extent to which the graph metrics contribute significantly to the discriminant function. A χ^2^ test was used to evaluate the significance of the discriminant function, tested at *P* < 0.05.

#### Association between fPET and fMRI graph metrics and cognition

To test the associations between the graph metrics and cognition, we transformed some of the behavioural data so that larger numbers indicate improved performance. In particular, we multiplying by −1 the stop signal reaction time, seconds per correct response in the digit substitution and category switch reaction time. The cognitive scores were then converted to Z-scores.

Canonical correlational analyses were used to test the hypothesis that the graph metrics will differentially associate with performance on the 10 cognition measures. The graph theory metrics at the whole brain level from the fPET and fMRI were included in separate analyses to return a cognitive profile associated with the graph metrics. Three potential canonical variates could be identified in each analysis, corresponding to the three graph metrics. The null hypothesis that the canonical correlation and all smaller ones are equal to zero was tested using the F-statistic from the Wilk’s test.

To further test the hypothesis that the fPET and fMRI graph metrics will both predict cognitive performance, we used a series of multiple regression analyses. Whole brain global and local efficiency and betweenness centrality were used as the independent variables with each of the 10 cognitive measures as the dependent variable in separate analyses (degree was again excluded). A second series of regression analyses was run with age group also included to assess whether age mediated the relationships between the graph metrics and cognition.

## Results

### Sample characteristics

The characteristics of the younger (*N* = 40) and older (*N* = 46) cohorts of participants are shown in Table 1. The mean age of the younger group was 28 (range 20–42) years and the older group 76 years (range 60–89). The younger group had a higher proportion of women (53%) and lower mean BMI (24.1 kg/m^2^) than those in the older group (46% women, BMI 25.7 kg/m^2^). However, these differences were not statistically significant. The older group had a higher mean fasting glucose than the younger group (5.2 mmol/L versus 4.8 mmol/L, *P* < 0.001).

**Table 1:** Mean and standard deviation (SD) of demographic and cognitive measures for older and younger adults, and *P*-value of statistical tests of age group differences.^a^

	Whole sample (*N* = 86)		Younger (*N* = 40)		Older (*N* = 46)		Younger versus older *P*-value
	Mean	SD	Mean	SD	Mean	SD	
Age (years)	53.5	24.8	27.9	6.2	75.8	6.0	0.000
Sex (number and % female)	42 (49%)		21 (53%)		21 (46%)		0.662
Education (years)	17.0	3.5	18.0	2.7	16.3	4.0	0.028
Body Mass Index (kg/m^2^)	25.0	4.1	24.1	4.6	25.7	3.6	0.075
Fasting blood glucose (mmol/L)	5.0	0.5	4.8	0.4	5.2	0.6	0.000
HVLT: Delayed recall (total)	8.2	2.7	9.3	2.5	7.3	2.6	0.000
HVLT: Recognition discrimination index	9.8	2.0	10.7	1.6	9.1	2.1	0.000
Digit span: Forward (longest)	6.7	1.3	6.7	1.5	6.8	1.1	0.587
Digit span: Backwards (longest)	5.4	1.3	5.7	1.4	5.1	1.2	0.066
Category switch: RT (seconds)	0.38	0.37	0.28	0.23	0.48	−0.43	0.011
Category switch: % correct trials	0.9	0.1	0.94	0.08	0.89	0.13	0.028
Digit substitution: Correct count	44.2	23.4	63.6	16.5	27.8	13.9	0.000
Digit substitution: Se per correct	5.9	14.4	3.40	9.31	8.01	17.45	0.144
Stop signal: Prob of reacting	48.9	11.2	51.0	12.6	47.2	9.7	0.115
Stop signal: RT (seconds)	27.5	0.1	0.27	0.05	0.28	0.06	0.170

HVLT, Hopkins verbal learning test.

^a^All *P*-values are for *t*-tests (two-tailed) of age group differences, except for sex, which is tested with χ^2^ (df = 1,86). An alpha of.05 was used to test for age group differences on each measure. *t*-test degrees of freedom were 84 for all measures except education and fasting blood glucose (81) and HVLT discrimination index (83).

### Cognitive function

Compared to the younger group, the older adult group showed worse memory, processing speed and cognitive flexibility (Table 1). In particular, older adults showed worse delayed recall and recognition discrimination index in the verbal learning test (both *P* < 0.001). Older adults completed fewer correct switch trials (*P* = 0.028). Older adults also had a slower reaction time in the category switch task (*P* = 0.011) and a lower correct count in the digit substitution task (*P* < 0.001). Digit span and stop signal task performance was similar in both age groups.

### Similarity of fPET and fMRI connectomes in the whole sample

A summary of the results of the similarity of the connectomes in fPET and fMRI, the fPET and fMRI age group differences and the association of graph metrics and cognition is provided in Table 2.

**Table 2:** Summary of the study results

**Similarity of fPET and fMRI connectomes**
There was moderate similarity in network strength and topology. The DICE coefficient was 0.67 for the top 50% of connections based on relative connection strength, and 0.42 at top 10% of connections. Within network-connectivity was higher in fMRI than fPET but between-network strength was similar.
**Age differences in fPET and fMRI connectivity**
Age differences were more widespread in fPET than fMRI, particularly for associative networks. Age differences in within-network connectivity were found in eight fPET networks compared to five fMRI networks. Younger adults had higher fPET within-network connectivity in the salience ventral attention, limbic, control and default networks. Older adults had higher fMRI within-network connections in the visual, somatomotor and dorsal attention networks and lower connectivity in the temporal network. Younger adults had higher between-network connectivity in 26 fPET network pairs, particularly between associative networks, and three fMRI network pairs. In contrast fMRI between-network connectivity was higher in 16 network pairs for older adults, including visual and associative networks.
**Age differences in graph metrics**
fPET connectomes showed lower local and global efficiency for older than younger adults, whereas the direction of age differences in fMRI global and local efficiency varied by region. Older adults had lower fPET global efficiency than older adults in 20 regions, mostly in the prefrontal cortex in the control, salience ventral attention and default networks. Older adults had lower fPET local efficiency than older adults in 36 regions across the brain, including in the prefrontal, parietal and motor cortices and the cingulate in the control, dorsal attention, salience ventral attention, default and somatomotor networks.
**Association and predictive strength of fPET and fMRI graph metrics and cognition**
A closer multivariate relationship was found between cognitive task performance and fPET than fMRI graph metrics. fPET graph metrics predicted episodic and working memory, cognitive control, visuospatial performance and processing speed. fMRI graph metrics predicted response inhibition.

**Figure 2:**
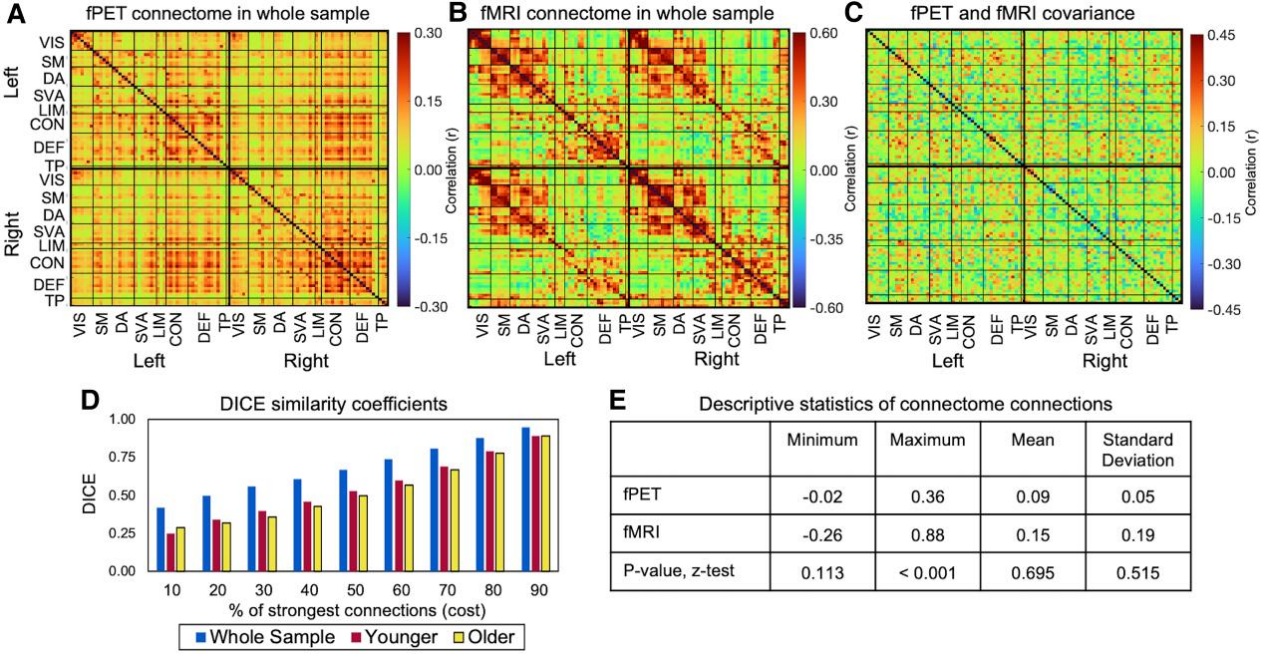
**fPET and fMRI connectomes and covariance among the whole sample (*N* = 86).** (**A**) fPET connectome, (**B**) fMRI connectome, (**C**) across-subject covariance of fPET and fMRI connectomes, (**D**) Dice–Sørensen coefficient (DICE coefficients) of fPET and fMRI connectome similarity at connection costs and (**E**) descriptive statistics and z-test of modality differences. VIS, visual; SM, somatomotor; DA, dorsal attention; SVA, salience ventral attention; LIM, limbic; CON, control; DEF, default; TP, temporal parietal.

Given that glucose metabolism contributes to the BOLD signal,^54^ we predicted that fPET and fMRI connectomes would have moderate similarity. While both whole sample averaged connectomes showed a clustering of connections in line with the functional network structure (Fig. 2), a notable characteristic of the connectomes was the differences and heterogeneity of the strength of the connections between modalities. The within- and between-network connections using fPET were in the order of *r* = 0.20 to 0.35, and were relatively homogenous in strength (SD = 0.05) (Fig. 2A). Although the connectivity strength was more heterogeneous using fMRI (SD = 0.19) than fPET, these differences were not statistically significant (*P* = 0.515). The maximum correlations were larger in the fMRI (r = 0.88) than the fPET (r = 0.36) (*P* < 0.001).

The DICE coefficient between the fPET and fMRI connectome was 0.67 for the top 50% of connections and 0.42 at top 10% of connections (Fig. 2D). It is notable that the fPET and fMRI connectomes showed low-to-moderate covariance, with values ranging from r = −0.36 to 0.40 (Fig. 2C). However, the fPET-fMRI covariance patterns did not tend to cluster together: regions within- and between-networks differed in covariance between the two modalities.

### fPET and fMRI connectome characteristics and age group differences

**Figure 3:**
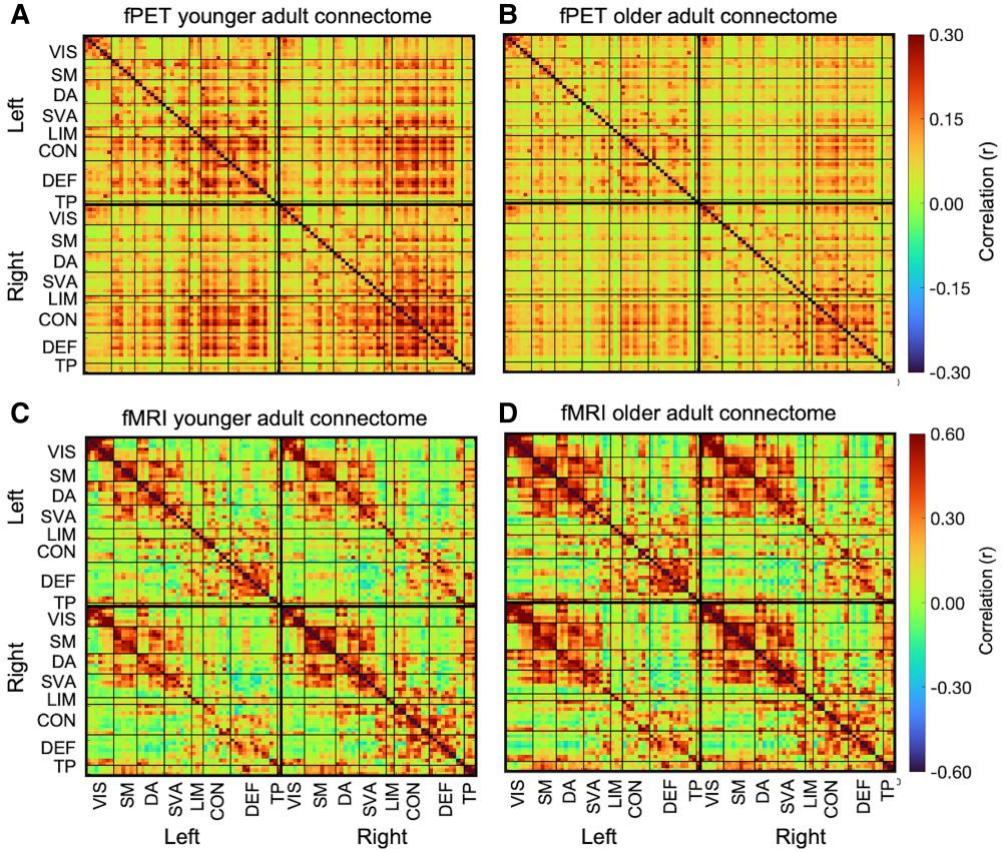
**fPET and fMRI connectomes for younger and older adults using the Schaefer parcellation.** (**A**) Younger (*N* = 40) and (**B**) older adult (*N* = 46) fPET metabolic connectomes, and (**C**) younger (*N* = 40) and (**D**) older adult (*N* = 46) fMRI connectomes. Connectomes are across the 100 nodes in 8 functional networks. VIS, visual; SM, somatomotor; DA, dorsal attention; SVA, salience ventral attention; LIM, limbic; CON, control; DEF, default; TP, temporal parietal. See Supplementary Table S1 for descriptive statistics and z-tests of differences in within- and between-network connectivity in the connectomes of younger (*N* = 40) and older (*N* = 46) adults in fPET and fMRI.

A salient qualitative characteristic of the group-averaged connectomes is the apparent age group differences in the fPET relative to the fMRI (Fig. 3). Using fPET, younger adults had stronger connections than older adults (indicated by the darker red-to-orange colours), particularly between the dorsal attention, salience ventral attention, control and default networks. In contrast, using fMRI, the correlations were visually similar in both age groups with both younger and older adults showing relatively high connectivity between the somatomotor and dorsal attention and salience ventral attention networks, as well as the control and default networks.

#### Within and between-network connectivity

**Figure 4:**
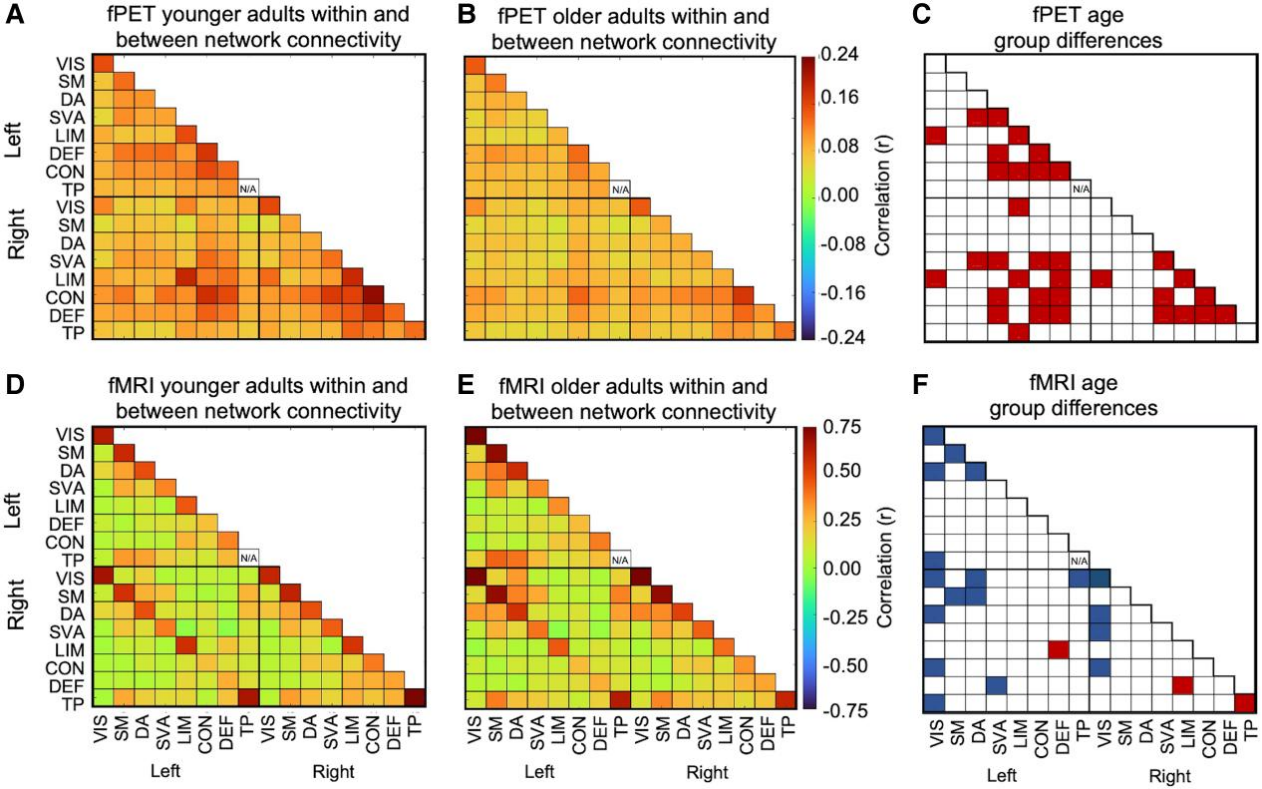
**fPET and fMRI within and between-network connectivity for younger and older adults.** Within and between-network connectivity was calculated by averaging the nodes within the networks for (**A**) younger (*N* = 40) and (**B**) older (*N* = 46) adults in fPET, and (**D**) younger (*N* = 40) and (**E**) older adults (*N* = 46) in fMRI. Within connectivity is shown in the diagonal cells and between connectivity on the off-diagonal cells of each matrix. Significance test (*t*-test, df = 84) of younger versus older adults, with shaded cells indicating a statistically significant age group differences at *P*-FDR < 0.05 (**C** and **F**). Red shading younger > older; blue shading older > younger. VIS, visual; SM, somatomotor; DA, dorsal attention; SVA, salience ventral attention; LIM, limbic; CON, control; DEF, default; TP, temporal parietal.

The strength, heterogeneity and topology of connections in the connectomes noted above for younger and older adults were evident when within- and between-network connectivity was calculated. There were also differences between fPET and fMRI in the connection strength for within-relative to between-network connectivity. The fPET connectomes had lower and more homogenous connection strength within-networks (Fig. 4A and B) than the fMRI connectomes (Fig. 4D and E). In contrast, the average fPET strength across all between-network connections was only slightly lower and showed less variability compared to the fMRI between-network connectivity (see Supplementary Table S1 for minimum, maximum, average and standard deviations of the connections).

Although fPET showed lower within-network connectivity maxima than fMRI, age group differences were more widespread in fPET than fMRI. Age differences in within-network connectivity were found in eight fPET networks compared to five fMRI networks (Fig. 4C and F). With fPET, younger adults had higher within-network connectivity strength than older adults bilaterally within the salience ventral attention, limbic, control and default networks. In contrast, with fMRI, the within-network connections were relatively high for both younger and older adults. Older adults also had higher fMRI within-network connections than younger adults in the left and right visual network and the left somatomotor and dorsal attention networks but low within network-connectivity in the right temporal network.

The topology and direction of age group differences in between-network connectivity were notably different for the fPET and fMRI. Using fPET, younger adults had higher between-network connectivity strength than older adults in 26 network pairs (Fig. 4C). However, when using fMRI, younger adults had lower connectivity than older adults in most, but not all network pairs (16 of 19) (Fig. 4F). For fPET, younger adults had higher network connectivity strength between the limbic, dorsal attention, salience ventral attention, control and default networks in the left and right hemispheres and across hemispheres. In the fMRI, young adults had higher connectivity strength between the left and right limbic and default networks. In contrast, older adults had higher fMRI network connectivity between the visual and dorsal attention, temporal and control networks. Younger adults also had lower fMRI connectivity between the dorsal attention and somatomotor networks.

#### Regional efficiency, centrality and degree

**Figure 5:**
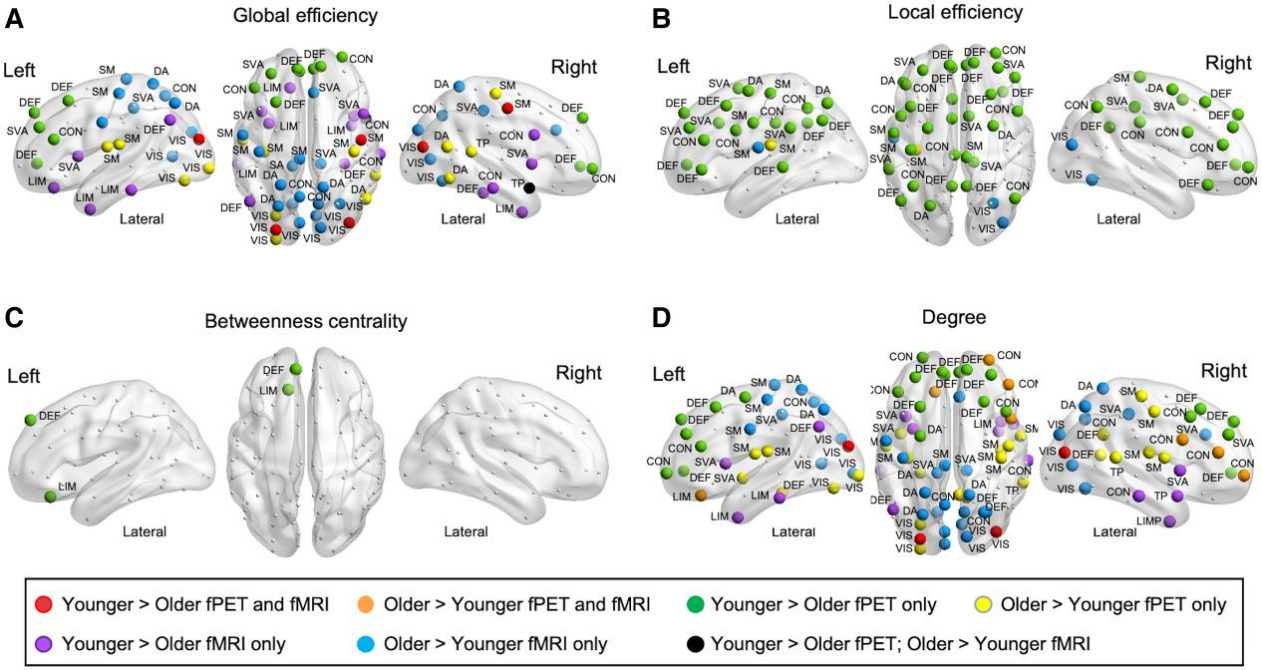
**fPET and fMRI age group differences in graph metrics.** Age group differences in (**A**) global efficiency, (**B**) local efficiency, (**C**) betweenness centrality and (**D**) degree. Regions are shown as coloured dots where there is a statistically significant difference at *P*-FDR < 0.05 from one-sided *t*-tests comparing younger (*N* = 40) and older (*N* = 46) adults, and the label indicates the network in which the region belongs. VIS, visual; SM, somatomotor; DA, dorsal attention; SVA, salience ventral attention; LIM, limbic; CON, control; DEF, default; TP, temporal parietal. For each region, the mean, standard deviations, age group effect sizes and *t*-tests are shown in Supplementary Tables S1–S4. Global efficiency (**A**) is average of the shortest inverse-distances between the node and all other nodes in the graph; local efficiency (**B**) is the average of shortest inverse-distances between the nodes within the neighbouring sub-graph; betweenness centrality (**C**) is the proportion of times that a node is part of a shortest-path between any two nodes within the graph; and degree (**D**) at each node defined as the number of edges from and to that node.

The strength, direction and topology of age group differences in the graph metrics were notably different between the fPET and fMRI. Significant age group differences were found in global efficiency for 20 fPET regions and 30 fMRI regions (Fig. 5A and Supplementary Table S2). Younger adults had higher fPET global efficiency than older adults in regions in the prefrontal cortex in the control, salience ventral attention and default networks. In contrast, in the fMRI, young adults had higher global efficiency in the insula, temporal regions of the limbic network and left inferior parietal lobule and right parahippocampal cortex in the default network. Older adults had higher fPET and fMRI global efficiency than younger adults in a number of visual and somatomotor regions. For fMRI, older adults also had higher global efficiency in regions of the superior parietal lobule and postcentral cortex in the dorsal attention network and the precuneus in the control network.

The pattern of results was relatively similar for degree, with significant age group differences in 31 fPET regions and 32 fMRI regions (Fig. 5D and Supplementary Table S5). Younger adults had higher fPET degree than older adults in regions in the prefrontal cortex but lower degree in regions in the visual and sensorimotor network, and posterior regions of the control and default networks. For fMRI, younger adults had lower degree in regions in the visual and somatomotor networks but higher degree in temporal regions in the limbic and temporal parietal networks, the temporal poles and the insula in the salience ventral attention network.

Significant age group differences were found in local efficiency for 36 fPET regions and four fMRI regions (Fig. 5B and Supplementary Table S3). Younger adults had higher fPET local efficiency than older adults across the brain, including regions in the prefrontal, parietal and motor cortices and the cingulate in the control, dorsal attention, salience ventral attention, default and somatomotor networks. In contrast, older adults had higher fMRI local efficiency than younger adults in regions of the visual and somatomotor networks and lower local efficiency in the precuneus in the control network.

Significant age group differences were found in betweenness centrality for two fPET regions but no fMRI regions (Fig. 5C and Supplementary Table S4). Younger adults had higher fPET betweenness centrality than older adults in the orbital prefrontal cortex in the limbic network and dorsal prefrontal cortex in the default network.

#### Network efficiency, centrality, and degree

**Figure 6:**
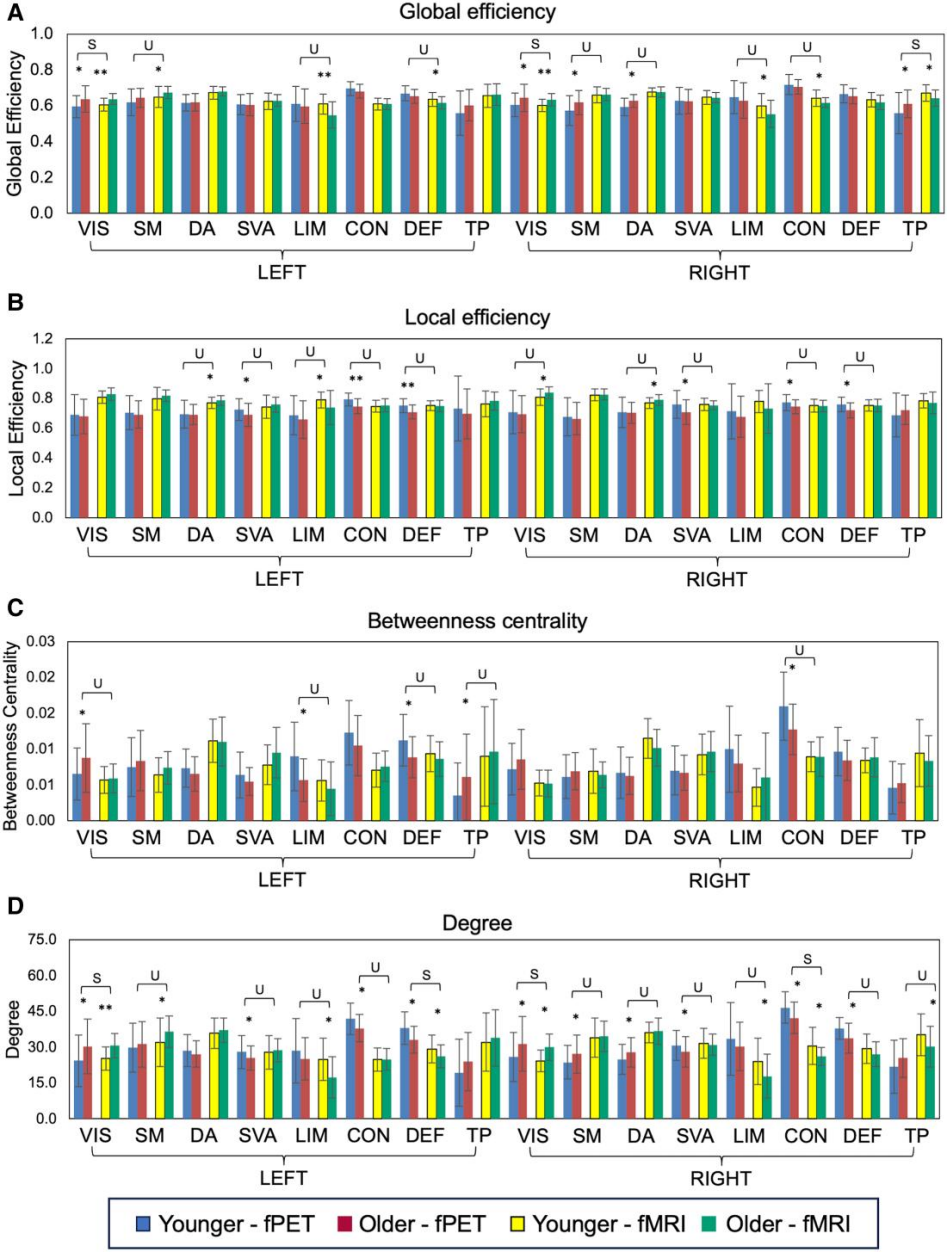
**Mean and standard deviation (error bars) of fPET and fMRI graph metrics for younger and older adults in the Schaefer networks.** Global efficiency (**A**) is average of the shortest inverse-distances between the node and all other nodes in the graph; local efficiency (**B**) is the average of shortest inverse-distances between the nodes within the neighbouring sub-graph; betweenness centrality (**C**) is the proportion of times that a node is part of a shortest-path between any two nodes within the graph; and degree (**D**) at each node defined as the number of edges from and to that node. Differences at *P-FDR < 0.05 and **P-FDR < 0.001 comparing younger (*N* = 40) versus older (*N* = 46) adults from one-sided *t*-tests. U, unique age group differences in one modality; S, Age group differences, same direction in both modalities. VIS, visual; SM, somatomotor; DA, dorsal attention; SVA, salience ventral attention; LIM, limbic; CON, control; DEF, default; TP, temporal parietal.

Age group differences at the network level largely followed the patterns at the region level. Older adults had significantly higher fPET and fMRI global efficiency than younger adults in the left and right visual network. Older adults also had higher fPET global efficiency than younger adults in the right somatomotor network and higher fMRI global efficiency in the left somatomotor network (Fig. 6A). Older adults had higher fPET and fMRI betweenness centrality and degree than younger adults in the visual and somatomotor networks (Fig. 6C and D). In contrast, younger adults had higher fPET local efficiency in the bilateral salience ventral attention, control and default networks (Fig. 6B) and higher fMRI local efficiency in the left limbic and right dorsal attention networks. Younger adults also had higher fPET betweenness centrality in the left limbic, default, temporal parietal and right control networks (Fig. 6C).

#### Relative predictive strength of fPET and fMRI graph metrics for age group

The discriminant function analysis was significant for the fPET whole brain graph metrics (Wilk’s Lambda = 0.795, χ^2^ = 18.9, *P* < 0.001) but not the fMRI metrics (Wilk’s Lambda = 0.958, χ^2^ = 3.5, *P* = 0.318). The probability of a correct age group classification based on the graph metrics was 60% and 61% for younger and older adults in the fPET, respectively; and 52% and 52% for younger and older adults in the fMRI.

#### Multivariate association between fPET and fMRI and cognition

**Figure 7:**
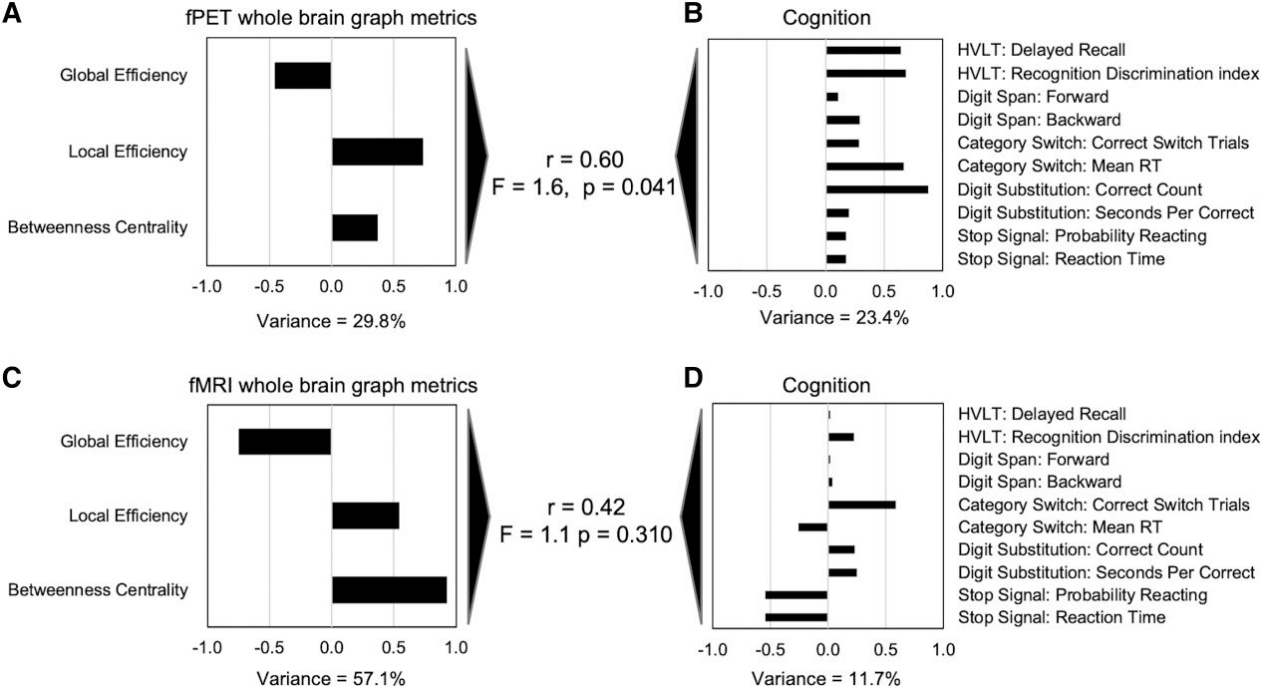
**Multivariate relationships between graph metrics and cognition.** Canonical correlations between the whole brain graph metrics from fPET and fMRI (**A** and **C**) and cognition (**B** and **D**) in the whole sample (*N* = 86). The r-value is the canonical correlation between the linear combinations of the graph metrics and cognition variables that maximally co-vary across subjects. F-statistic is Wilk’s test of the null hypothesis that the canonical correlation and all smaller ones are equal to zero and was significant for one canonical variate for fPET but not fMRI. The correlations on each variable set represent the strength of the association between the variable and the canonical variate. Variance explained is the percentage of variance explained by the variables in their variate. Stop signal reaction time, seconds per correct response in the digit substitution and category switch reaction time were multiplied by −1 so that higher scores reflect better performance. The test of difference between two correlations = 1.6; *P* = 0.114 (two-tailed).

The canonical correlation analyses revealed a closer multivariate relationship between the fPET than fMRI whole brain graph metrics and cognitive task performance. The canonical correlation analysis of the fPET graph metrics identified one significant canonical mode (Fig. 7). The linear combinations of the fPET graph metrics and the cognition measures were significantly correlated with each other, with a moderately high correlation coefficient of 0.60 (*P* = 0.041). However, for the fMRI analysis, the linear combinations of the graph metrics and the cognition measures were lower and not significant (r = 0.42, *P* = 0.310). For the fPET, higher local efficiency in particular was associated with better delayed recall and discrimination index score in the HVLT, a faster category switch reaction time and higher score in the digit substitution task.

#### Relative predictive strength of fPET and fMRI graph metrics for cognition

The multiple regression analyses revealed that the fPET graph metrics were significantly associated with more cognitive measures than the fMRI graph metrics (Table 3). The regression analyses were significant for the discrimination index in the HVLT (*P* = 0.021), correct number of responses in the digit substitution task (*P* < 0.001), reaction time in the category switch task (*P* = 0.044), and probability of reaction in the stop signal task (*P* = 0.037). The regression analysis for the backward digit span approached significance (*P* = 0.051). Higher fPET local efficiency was associated with better recognition discrimination in the HVLT, more correct responses in the digit substitution task and faster reaction time in the category switch tasks. Higher fPET betweenness centrality was also associated with better performance in the backward digit span task and digit substitution task. Higher global efficiency and betweenness centrality in the fMRI was associated with lower probability of reacting in the stop signal task.

**Table 3:** Regression analyses predicting cognitive performance from fPET and fMRI whole brain graph metrics from the Schaefer atlas

	HVLT: delayed recall	HVLT: discrim. index	Digit span: forward	Digit span: back	Category switch: % Trials	Category switch: RT	Dig sub: number correct	Dig sub: sec per correct	Stop signal: Pr reacting	Stop signal: RT
ANOVA F	1.7	2.7	0.1	2.2	1.8	2.3	4.5	0.5	2.4	0.9
ANOVA *P*	0.132	0.021	0.995	0.051	0.109	0.044	<0.001	0.784	0.037	0.498
Variance explained	12%	17%	1%	14%	12%	15%	26%	4%	15%	6%
fPET: Global efficiency	0.08	0.25	0.04	−0.05	−0.15	0.20	0.11	−0.17	−0.03	0.04
fPET: Local efficiency	0.40*	0.53*	0.03	−0.01	0.09	0.47*	0.55*	0.02	0.14	0.13
fPET: Betweenness centrality	0.15	0.20	0.07	0.33*	0.15	0.15	0.23*	0.12	−0.12	0.00
fMRI: Global efficiency	0.18	−0.15	−0.08	0.49	0.32	0.21	0.22	0.02	−0.89*	−0.02
fMRI: Local efficiency	−0.09	−0.11	0.05	−0.09	−0.13	−0.12	−0.26	0.07	−0.15	−0.01
fMRI: Betweenness centrality	0.25	0.10	−0.11	0.43	0.64	0.15	0.49	0.10	−0.87*	−0.23

The ANOVA results for the overall models are shown in the top three rows and the standardized beta weights for each graph metric’s relationship with cognition in subsequent rows. Significant beta weights are indicated at *P < 0.05 and **P < 0.001.

Discrim, discrimination; Dig Sub, digit substitution; Pr reacting, probability of reacting.

When age group was included in the regression analyses (see Supplementary Table S6), a significant association remained between fPET local efficiency and the HVLT discrimination index and betweenness centrality in the backward digit span task (both *P* < 0.05). However, fPET local efficiency was no longer a significant predictor of the correct number of responses in the digit substitution task and category switch reaction time. Higher fMRI global efficiency and betweenness centrality remained significantly associated with probability of reacting in the stop signal task (*P* < 0.001).

## Discussion

In this simultaneous fPET/fMRI study, we found that whole-brain metabolic fPET connectomes have greater predictive utility for age and cognition than functional fMRI connectomes. Although fMRI connectomes showed higher maximum connectivity strength than fPET connectomes, fPET connectomes more strongly predicted age, revealed more age group differences in graph metrics and predicted cognition across more domains than fMRI connectomes. Together, these results build on our previous conclusion that fPET and fMRI provide complementary insights into brain function^24,29^ by suggesting that metabolic connectivity has greater utility for understanding metabolic network changes in ageing and as a potential biomarker for functional and cognitive declines in ageing and neurodegeneration.^55^

As we hypothesized, the whole-brain fPET connectome was moderately similar in topology to the connectome calculated from fMRI data. These results were expected given that glucose metabolism contributes to the BOLD signal.^54^ We also found noteworthy differences between the modalities; some differences that supported and others that contrasted with our hypotheses. Contrary to our hypothesis, age group differences in connectivity were more widespread in the fPET than the fMRI connectomes. As we expected, younger adults had higher fPET within-network connectivity in several associative networks, namely, bilaterally in the salience ventral attention, limbic, control and default networks. In fMRI, older adults also showed lower within-network connectivity, albeit in a smaller number of networks. Older adults had higher fMRI within-network connections than younger adults, mostly in primary processing and attentional networks. Older adults also had higher fMRI local efficiency in a number of regions in the dorsal attention, salience ventral attention and default networks. When between-network connectivity and global efficiency were assessed, the topology and direction of the age differences between the imaging modalities was also noteworthy. Younger adults had significantly higher fPET between-network connectivity and higher global efficiency than older adults, again primarily in the associative networks. A smaller number of associative networks showed age group differences in fMRI. Moreover, older adults had higher fMRI between-network connectivity than younger adults between the visual networks and the dorsal attention, control and default networks. Our fMRI results are consistent with previous research in terms of the primary processing and attentional networks. However, our results also diverge from previous fMRI research, which has also largely shown higher connectivity among older than younger adults between associative networks.^32^

A likely reason for the modality differences relates to the fact that fMRI and fPET are indexing different aspects of neuronal activity. The BOLD signal stems from a regional increase in cerebral blood flow, metabolic rate of oxygen, lactate and a decrease in oxygen extraction fraction.^9^ In contrast, FDG-PET is less likely to be affected by blood flow or haemodynamic differences and provides a more direct measure of glucose uptake at the excitatory post-synaptic neuron.^56,57^ As such, the differences we found between metabolic fPET and functional fMRI connectivity likely reflect at least in part differences in the timing, rate and severity of physiological changes in ageing, as indexed by the fPET and fMRI signals. For example, alterations to neurovascular coupling,^58^ cerebral blood flow, metabolic rate of oxygen, lactate, and oxygen extraction fraction can be differentially affected in ageing.^36,37,59,60^ Health status, such as blood pressure, insulin resistance, adiposity and level of tau or amyloid pathology may be impacting the fEPT and fMRI signals. The potential moderating role of these factors on cognitive ageing and fPET and fMRI connectivity could be assessed in future studies.

A second and related possible reason for the modality differences relates to the underlying rates of CMR_GLC_, cerebral blood flow and neurovascular coupling. Previous research has shown that blood flow and glucose metabolism can be uncoupled^35,61^ or vary in their association across brain regions in ageing,^62,63^ and even change in opposite directions from rests to task conditions.^64^ Similarly, there is regional variability between the BOLD signal and local field potentials in EEG,^65^ suggesting that neuronal activity and the BOLD signal are not always well coupled.

As we hypothesized, fPET and fMRI graph metrics were differentially associated with cognition across domains.^24^ However, we also found that differences in graph metrics predicted cognition across more domains in fPET than fMRI. Higher fPET whole-brain local efficiency was associated with better episodic and working memory, visuospatial performance, and speed of response inhibition. Higher global efficiency and betweenness centrality in the fMRI was associated with response inhibition. Similarly, the multivariate canonical relationship between the graph metrics and the cognitive domains was significant for fPET but not fMRI, although the level of the correlations was similar between modalities. The discriminant function analysis was also significant for fPET but not fMRI. Our results build on previous studies suggesting that fPET and fMRI are differentially associated with cognition.^24,29,66,67^ They indicate that there is a reconfiguration of the dynamic metabolic signals in ageing that is different to vascular and haemodynamic alterations and is more strongly linked to cognitive performance. They also suggest that metabolic connectivity has potential to serve as biomarkers for cognitive decline and to help guide early interventions to slow age- or disease-related cognitive decline. For example, metabolic connectivity could play a role as diagnostic tools for the early detection of brain changes in health and disease. It could also be used to monitor the brain’s response to lifestyle interventions or pharmacological and clinical treatments targeting metabolic health.

We assessed metabolic networks in the PET data at a temporal scale of 16 s. Additional advances in PET reconstruction and filtering techniques^23,68^ may further reduce the temporal resolution of fPET to that of fMRI. On the other hand, some evidence from animal models suggests that changes in metabolic flux emerge in less than half a second after neuronal activity^69^ but persist for tens of seconds.^64^ High-temporal resolution fPET studies in humans show that the FDG signal does not decrease to the baseline level immediately after the task stimulation.^22,28^ For example, the FDG signal continued to increase during a 32 s working memory task with a return to baseline at ∼10 s after task completion.^70^ Together these studies suggest that a timescale of around 10 s may set a limit on the ability of fPET to capture higher-frequency metabolic components of neural signals and their coherence across large-scale brain networks at rest. Additional research at different temporal scales is needed to further test this possibility.

A limitation of this study is the cross-sectional design. It is possible that the age differences we identified reflect underlying cohort or unmeasured differences between the two groups. It is also possible that alterations in metabolic connectivity are non-linear across the lifespan,^32^ something we cannot test due to the absence of middle age adults in our sample. We also used a convenient sampling approach and note that our sample appears to be relatively healthy based on BMI and fasting glucose and to have a high level of education. Additional studies are needed to replicate and extend our findings with other ageing samples. Additional research, including longitudinal studies, is also needed to assess fPET changes across the adult lifespan and to characterize the timeframe and mechanisms driving connectivity alterations in normative ageing.

In conclusion, functional brain networks can be reliably derived from fPET data, reflecting the close link between neural activity and dynamic glucose metabolism across the brain network. fPET has greater predictive utility for age and cognition than fMRI. There is a reconfiguration of metabolic networks in ageing that is different to haemodynamic alterations and is more strongly linked to cognitive performance. These results highlight the important role that dynamic glucose metabolism plays in neuronal communication across the brain. They suggest that interventions to help maintain efficient brain metabolism in early and mid-adulthood may help to conserve metabolic connectivity and cognitive health in ageing. They also underscore the utility of metabolic connectivity to understand brain network changes in health and disease and as a potential biomarker for functional and cognitive declines in ageing and neurodegeneration.

## Supplementary Material

fcaf075_Supplementary_Data

## Data Availability

The datasets used and/or analysed during the current study available from the corresponding author on reasonable request.

## Code availability statement

No custom code was developed in this work.
