# Supplementary material for: Metabolic connectivity has greater predictive utility for age and cognition than functional connectivity
Source: Brain Commun. 2025 Feb 18;7(1):fcaf075. doi: 10.1093/braincomms/fcaf075 (PMC11851278; doi:10.1093/braincomms/fcaf075)
Supplement: fcaf075_Supplementary_Data [file fcaf075_supplementary_data.pdf]

Supplementary Information

Metabolic connectivity has greater predictive utility for age and cognition than functional connectivity.

Deery, Liang, Moran, Egan, Jamadar

Table of Contents

1. Supplementary Methods 2
1.1 MR-PET Data Acquisition 2
1.2 Correction for Partial Volume Effects 2
2. Supplementary Results from the Main Manuscript 3
3. Anatomical Parcellation: Harvard Oxford Atlas 9

1. Supplementary Methods

1.1 MR-PET Data Acquisition

Participants underwent a 90-minute simultaneous MR-PET scan in a Siemens (Erlangen) Biograph 3-Tesla molecular MR scanner. Participants were directed to consume a high-protein/low-sugar diet for the 24 hours prior to the scan. They were also instructed to fast for six hours and to drink 2–6 glasses of water. Prior to FDG infusion, participants were cannulated in the vein in each forearm and a 10ml baseline blood sample taken. At the beginning of the scan, half of the 260 MBq FDG tracer was administered via the left forearm as a bolus, providing a strong PET signal from the beginning of the scan. The remaining 130 MBq of the FDG tracer dose was infused at a rate of 36ml/hour over 50 minutes, minimising the amount of signal decay over the course of the data acquisition. We have previously demonstrated that this protocol provides a good balance between a fast increase in signal-to-noise ratio at the start of the scan, and maintenance of signal-to-noise ratio over the duration of the scan [1].

Participants were positioned supine in the scanner bore with their head in a 32-channel radiofrequency head coil and were instructed to lie as still as possible. The scan sequence was as follows. Non-functional MRI scans were acquired during the first 12 minutes, including a T1 3DMPRAGE (TA = 3.49 min, TR = 1640ms, TE = 234ms, flip angle = 8°, field of view = 256 × 256 mm2, voxel size = 1.0 × 1.0 × 1.0 mm3, 176 slices, sagittal acquisition) and T2 FLAIR (TA = 5.52 min, TR = 5,000ms, TE = 396ms, field of view = 250 × 250 mm2, voxel size = .5 × .5 × 1 mm3, 160 slices) to image the anatomical grey and white matter structures, respectively. Thirteen minutes into the scan, list-mode PET (voxel size = 1.39 x 1.39 x 5.0mm3) and T2* EPI BOLD-fMRI (TA = 40 minutes; TR = 1000ms, TE = 39ms, FOV = 210 mm2, 2.4 × 2.4 × 2.4 mm3 voxels, 64 slices, ascending axial acquisition) sequences were initiated. A 40-minute resting-state scan was undertaken in naturalistic viewing conditions watching a movie of a drone flying over the Hawaii Islands. At 53 minutes, pseudo-continuous arterial spin labelling (pc-ASL) began, and at 58 minutes, diffusion-weighted imaging (DWI) was acquired with 71 directions to index white matter connectivity. pcASL, DWI and fMRI results are not reported here.

Plasma radioactivity levels were measured throughout the duration of the scan. Beginning at 10-minutes post infusion onset, 5ml blood samples were taken from the right forearm using a vacutainer at 10-minute intervals for a total of nine samples. The blood sample were immediately placed in a Heraeus Megafuge 16 centrifuge (ThermoFisher Scientific, Osterode, Germany) and spun at 2,000 rpm (RCF ~ 515g) for 5 minutes. 1,000-μL plasma was pipetted, transferred to a counting tube, and placed in a well counter for four minutes. The count start time, total number of counts, and counts per minute were recorded for each sample.

1.2 Correction for Partial Volume Effects

PET images were corrected for partial volume effects using the modified Müller-Gartner method implemented in PetSurf (https://surfer.nmr.mgh.harvard.edu/fswiki/PetSurfer) [2,3]. The method corrects for white matter spill in and grey matter spill out of the PET signal. The equation subtracts from the grey matter voxel signal the white matter signal (convoluted by the point spread function) and divides by the grey matter signal. This division can introduce over-correction at the grey matter boundary, and hence a grey matter binary mask is recommended, with the threshold level needing to be chosen. A grey matter threshold of 20-30% is recommended in ageing because atrophy can influence results [2]. For our analyses, we chose a 25% grey matter threshold and surface-based spatial smoothing [3]. We used a Gaussian kernel with a full width at half maximum of 8mm to increase the signal-to-noise ratio. Subcortical structures were partial volume corrected and spatially smoothed in volume space and merged with the cortical data.

2. Supplementary Results from the Main Manuscript

Supplementary Table 1. Descriptive statistics and z-tests of differences in within- and between-network connectivity in the connectomes of younger (N = 40) and older (N = 46) adults in fPET and fMRI. The connectomes are shown in Figure 3 of the main manuscript.

	Within Network Connectivity				Between Network Connectivity			
fPET								
	Min	Max	Mean	SD	Min	Max	Mean	SD
Younger	0.09	0.22	0.14	0.04	0.04	0.22	0.10	0.03
Older	0.06	0.17	0.11	0.03	0.04	0.17	0.08	0.02
fMRI								
	Min	Max	Mean	SD	Min	Max	Mean	SD
Younger	0.23	0.74	0.48	0.14	-0.03	0.67	0.15	0.13
Older	0.24	0.78	0.49	0.18	-0.03	0.83	0.18	0.14
fPET vs fMRI, p-value (Z-test)								
	Min	Max	Mean	SD	Min	Max	Mean	SD
Younger	.534	.002	.100	.664	.763	.012	.827	.665
Older	.392	.000	.048	.481	.745	.000	.637	.575

Supplementary Table 2. Mean, standard deviation and effect sizes (Cohen’s D) of regional global efficiency for younger (N = 40) and older (N = 86) adults in fPET and fMRI, and t-tests of age group differences.

	fPET								fMRI							
	Younger		Older		Younger vs Older				Younger		Older		Younger vs Older			
	Mean	SD	Mean	SD	Cohen's D	*t* -value	p	p-FDR	Mean	SD	Mean	SD	Cohen's D	*t* -value	p	p-FDR
Visual Central: Extra Striate Cortex 1 L	0.60	0.10	0.66	0.10	-0.55	-2.6	0.006	0.037	0.61	0.07	0.63	0.05	-0.21	-1.0	0.167	0.232
Visual Central: Extra Striate Cortex 2 L	0.63	0.10	0.69	0.10	-0.64	-3.0	0.002	0.020	0.58	0.08	0.60	0.07	-0.32	-1.5	0.073	0.133
Visual Central: Striate Cortex 1 L	0.59	0.11	0.64	0.09	-0.48	-2.2	0.014	0.061	0.57	0.07	0.60	0.06	-0.46	-2.1	0.018	0.057
Visual Central: Extra Striate Cortex 3 L	0.59	0.09	0.65	0.08	-0.71	-3.3	0.001	0.015	0.61	0.05	0.66	0.05	-0.93	-4.3	0.000	0.001
Visual Peripheral: Extra Striate Inferior 1 L	0.54	0.09	0.57	0.12	-0.28	-1.3	0.098	0.177	0.63	0.07	0.66	0.06	-0.36	-1.7	0.049	0.107
Visual Peripheral: Striate Cortex Calcarine 1 L	0.60	0.09	0.58	0.12	0.10	0.5	0.316	0.369	0.61	0.07	0.66	0.06	-0.69	-3.2	0.001	0.007
Visual Peripheral: Extra Striate CortexSup 1 L	0.58	0.10	0.59	0.08	-0.09	-0.4	0.339	0.383	0.63	0.07	0.67	0.05	-0.75	-3.5	0.000	0.005
Somatomotor A: 1 L	0.62	0.14	0.64	0.09	-0.17	-0.8	0.221	0.283	0.65	0.08	0.69	0.05	-0.55	-2.6	0.006	0.026
Somatomotor A: 2 L	0.64	0.15	0.65	0.09	-0.12	-0.6	0.285	0.347	0.65	0.07	0.70	0.04	-0.80	-3.7	0.000	0.004
Somatomotor B: Auditory 1 L	0.69	0.08	0.69	0.08	-0.05	-0.2	0.411	0.446	0.66	0.08	0.67	0.07	-0.17	-0.8	0.217	0.274
Somatomotor B: S2 1 L	0.47	0.17	0.57	0.11	-0.70	-3.3	0.001	0.015	0.67	0.07	0.66	0.08	0.25	1.2	0.122	0.183
Somatomotor B: S2 2 L	0.58	0.09	0.63	0.08	-0.61	-2.8	0.003	0.024	0.68	0.07	0.66	0.05	0.29	1.3	0.095	0.161
Somatomotor B: Central 1 L	0.59	0.09	0.61	0.09	-0.29	-1.4	0.088	0.177	0.58	0.09	0.63	0.07	-0.62	-2.9	0.003	0.012
Dorsal Attention A: Temporal Occipital 1 L	0.60	0.11	0.63	0.09	-0.33	-1.5	0.065	0.144	0.69	0.06	0.67	0.05	0.32	1.5	0.070	0.133
Dorsal Attention A: Parietal Occipital 1 L	0.55	0.10	0.59	0.11	-0.41	-1.9	0.032	0.085	0.69	0.06	0.68	0.08	0.26	1.2	0.112	0.179
Dorsal Attention A: Superior Parietal Lobule 1 L	0.67	0.07	0.67	0.07	0.00	0.0	0.493	0.494	0.66	0.05	0.68	0.05	-0.50	-2.3	0.012	0.043
Dorsal Attention B: Post Central 1 L	0.60	0.09	0.58	0.12	0.25	1.2	0.125	0.199	0.67	0.07	0.69	0.04	-0.31	-1.4	0.077	0.138
Dorsal Attention B: Post Central 2 L	0.53	0.16	0.58	0.11	-0.36	-1.7	0.051	0.116	0.65	0.07	0.67	0.05	-0.34	-1.6	0.061	0.120
Dorsal Attention B: Post Central 3 L	0.62	0.10	0.61	0.08	0.11	0.5	0.302	0.360	0.67	0.05	0.70	0.04	-0.63	-2.9	0.002	0.012
Dorsal Attention B: Frontal Eye Fields 1 L	0.66	0.11	0.61	0.10	0.48	2.2	0.015	0.061	0.67	0.08	0.68	0.06	-0.20	-0.9	0.183	0.244
Salience Ventral Attention A: Parietal Operculum 1 L	0.64	0.08	0.62	0.08	0.27	1.3	0.107	0.181	0.67	0.05	0.65	0.07	0.36	1.7	0.049	0.107
Salience Ventral Attention A: Insula: 1 L	0.43	0.18	0.51	0.15	-0.45	-2.1	0.020	0.069	0.63	0.08	0.59	0.12	0.38	1.8	0.040	0.098
Salience Ventral Attention A: Insula: 2 L	0.55	0.16	0.60	0.08	-0.42	-1.9	0.028	0.081	0.64	0.06	0.62	0.06	0.49	2.2	0.014	0.045
Salience Ventral Attention A: Parietal Medial 1 L	0.57	0.12	0.60	0.11	-0.24	-1.1	0.136	0.200	0.67	0.08	0.71	0.06	-0.64	-2.9	0.002	0.012
Salience Ventral Attention A: Frontal Medial 1 L	0.61	0.11	0.61	0.12	0.03	0.1	0.445	0.459	0.62	0.08	0.64	0.06	-0.20	-0.9	0.182	0.244
Salience Ventral Attention B: Lateral Prefrontal Cortex 1 L	0.73	0.07	0.69	0.06	0.60	2.8	0.003	0.025	0.57	0.08	0.61	0.08	-0.42	-1.9	0.029	0.079
Salience Ventral Attention B: Medial Posterior Prefrontal 1 L	0.63	0.08	0.59	0.12	0.36	1.7	0.049	0.114	0.62	0.08	0.63	0.07	-0.25	-1.2	0.124	0.183
Limbic A Temporal Pole 1 L	0.67	0.12	0.67	0.08	0.04	0.2	0.425	0.449	0.59	0.08	0.50	0.14	0.84	3.9	0.000	0.003
Limbic A: Temporal Pole 2 L	0.53	0.15	0.53	0.16	0.00	0.0	0.494	0.494	0.61	0.08	0.57	0.08	0.51	2.3	0.011	0.042
Limbic B: Orbital Frontal Cortex 1 L	0.66	0.10	0.61	0.13	0.41	1.9	0.031	0.085	0.63	0.07	0.58	0.07	0.74	3.4	0.000	0.005
Control A: Intraparietal Sulcus 1 L	0.69	0.07	0.67	0.06	0.25	1.2	0.121	0.198	0.62	0.05	0.62	0.06	-0.01	0.0	0.489	0.494
Control A: Lateral Prefrontal Cortex 1 L	0.76	0.05	0.73	0.07	0.58	2.7	0.004	0.031	0.59	0.06	0.60	0.05	-0.08	-0.4	0.362	0.393
Control A: Lateral Prefrontal Cortex 2 L	0.64	0.08	0.62	0.07	0.25	1.2	0.123	0.198	0.64	0.06	0.61	0.06	0.46	2.1	0.017	0.056
Control B: Lateral Prefrontal Cortexv 1 L	0.75	0.07	0.72	0.06	0.44	2.0	0.022	0.069	0.62	0.09	0.59	0.08	0.26	1.2	0.120	0.183
Control C: Precuneus 1 L	0.66	0.07	0.67	0.08	-0.18	-0.8	0.209	0.274	0.58	0.07	0.60	0.06	-0.37	-1.7	0.046	0.105
Control C: Precuneus 2 L	0.61	0.08	0.62	0.12	-0.13	-0.6	0.271	0.335	0.61	0.07	0.66	0.06	-0.85	-3.9	0.000	0.003
Control C: Cingulate Posterior 1 L	0.60	0.13	0.63	0.12	-0.30	-1.4	0.084	0.172	0.58	0.07	0.60	0.06	-0.17	-0.8	0.212	0.271
Default A: Dorsal Prefrontal Cortex 1 L	0.68	0.10	0.62	0.08	0.70	3.2	0.001	0.015	0.60	0.08	0.60	0.09	-0.03	-0.2	0.439	0.458
Default A: Precuneus Posterior Cingulate Cortex1 L	0.69	0.09	0.71	0.12	-0.19	-0.9	0.196	0.265	0.62	0.06	0.62	0.06	0.13	0.6	0.269	0.332
Default A: Medial Prefrontal Cortex 1 L	0.67	0.07	0.62	0.07	0.61	2.8	0.003	0.024	0.63	0.07	0.61	0.08	0.27	1.2	0.111	0.179
Default B: Temp 1 L	0.57	0.16	0.60	0.08	-0.21	-1.0	0.168	0.237	0.65	0.07	0.64	0.06	0.27	1.3	0.105	0.175
Default B: Temp 2 L	0.60	0.09	0.62	0.08	-0.17	-0.8	0.211	0.274	0.65	0.08	0.62	0.06	0.34	1.6	0.060	0.120
Default B: Inferior Parietal Lobule 1 L	0.71	0.07	0.69	0.07	0.28	1.3	0.103	0.177	0.66	0.06	0.62	0.06	0.69	3.2	0.001	0.007
Default B: Dorsal Prefrontal Cortex 1 L	0.75	0.05	0.68	0.08	1.03	4.8	0.000	0.000	0.65	0.07	0.64	0.07	0.10	0.5	0.327	0.372
Default B: Lateral Prefrontal Cortex 1 L	0.68	0.08	0.66	0.07	0.28	1.3	0.103	0.177	0.62	0.08	0.62	0.06	0.00	0.0	0.494	0.494
Default B: Ventral Prefrontal Cortex 1 L	0.58	0.12	0.59	0.12	-0.04	-0.2	0.419	0.449	0.62	0.06	0.59	0.11	0.31	1.4	0.080	0.140
Default B: Ventral Prefrontal Cortex 2 L	0.75	0.08	0.73	0.08	0.28	1.3	0.103	0.177	0.63	0.06	0.61	0.06	0.40	1.9	0.033	0.085
Default C: RetroSuperior Parietal Lobuleenial 1 L	0.54	0.11	0.60	0.12	-0.44	-2.0	0.023	0.071	0.61	0.05	0.61	0.05	-0.03	-0.1	0.446	0.460
Default C: Parahippocampal Cortex 1 L	0.45	0.14	0.51	0.15	-0.40	-1.9	0.034	0.085	0.60	0.07	0.57	0.11	0.23	1.1	0.144	0.203
Temporal Parietal 1 L	0.56	0.12	0.60	0.09	-0.42	-1.9	0.028	0.081	0.66	0.07	0.66	0.06	-0.13	-0.6	0.282	0.343
Visual Central: Extra Striate Cortex 1 R	0.61	0.10	0.63	0.09	-0.27	-1.2	0.111	0.185	0.61	0.05	0.64	0.05	-0.64	-3.0	0.002	0.011
Visual Central: Extra Striate Cortex 2 R	0.66	0.10	0.71	0.11	-0.47	-2.2	0.017	0.063	0.57	0.07	0.59	0.06	-0.24	-1.1	0.135	0.193
Visual Central: Extra Striate Cortex 3 R	0.59	0.09	0.67	0.09	-0.84	-3.9	0.000	0.005	0.61	0.05	0.64	0.05	-0.62	-2.9	0.002	0.012
Visual Peripheral: Striate Cortex Calcarine 1 R	0.60	0.12	0.62	0.09	-0.23	-1.1	0.142	0.203	0.58	0.06	0.63	0.06	-0.77	-3.5	0.000	0.005
Visual Peripheral: Extra Striate Inferior 1 R	0.54	0.11	0.57	0.13	-0.24	-1.1	0.135	0.200	0.66	0.08	0.68	0.06	-0.41	-1.9	0.031	0.081
Visual Peripheral: Extra Striate Superior 1 R	0.58	0.10	0.60	0.08	-0.28	-1.3	0.097	0.177	0.62	0.05	0.66	0.06	-0.72	-3.3	0.001	0.006
Somatomotor A: 1 R	0.49	0.15	0.57	0.09	-0.67	-3.1	0.001	0.018	0.66	0.06	0.69	0.04	-0.49	-2.3	0.012	0.043
Somatomotor A: 2 R	0.52	0.16	0.60	0.09	-0.66	-3.0	0.002	0.020	0.65	0.07	0.66	0.05	-0.11	-0.5	0.304	0.362
Somatomotor A: 3 R	0.51	0.17	0.56	0.11	-0.37	-1.7	0.044	0.108	0.67	0.06	0.69	0.04	-0.25	-1.2	0.123	0.183
Somatomotor A: 4 R	0.58	0.17	0.61	0.13	-0.20	-0.9	0.174	0.241	0.67	0.05	0.68	0.04	-0.32	-1.5	0.072	0.133
Somatomotor B: Auditory 1 R	0.69	0.09	0.71	0.07	-0.20	-0.9	0.184	0.252	0.68	0.07	0.66	0.07	0.26	1.2	0.113	0.179
Somatomotor B: S2 1 R	0.47	0.17	0.54	0.14	-0.46	-2.1	0.019	0.067	0.66	0.06	0.66	0.07	0.10	0.4	0.328	0.372
Somatomotor B: S2 2 R	0.56	0.11	0.61	0.08	-0.50	-2.3	0.011	0.054	0.67	0.05	0.66	0.06	0.11	0.5	0.313	0.368
Somatomotor B: Central 1 R	0.55	0.13	0.60	0.07	-0.47	-2.2	0.017	0.063	0.60	0.09	0.60	0.08	-0.08	-0.4	0.358	0.393
Dorsal Attention A: Temporal Occipital 1 R	0.58	0.09	0.62	0.08	-0.55	-2.6	0.006	0.037	0.68	0.05	0.66	0.06	0.35	1.6	0.057	0.120
Dorsal Attention A: Parietal Occipital 1 R	0.61	0.08	0.65	0.07	-0.54	-2.5	0.007	0.037	0.69	0.06	0.67	0.06	0.41	1.9	0.029	0.079
Dorsal Attention A: Superior Parietal Lobule 1 R	0.61	0.13	0.65	0.07	-0.47	-2.2	0.016	0.063	0.64	0.06	0.66	0.05	-0.38	-1.8	0.042	0.100
Dorsal Attention B: Post Central 1 R	0.52	0.15	0.56	0.12	-0.32	-1.5	0.073	0.156	0.68	0.05	0.67	0.06	0.20	0.9	0.181	0.244
Dorsal Attention B: Post Central 2 R	0.58	0.13	0.61	0.07	-0.31	-1.4	0.081	0.168	0.67	0.05	0.71	0.05	-0.77	-3.5	0.000	0.005
Dorsal Attention B: Frontal Eye Fields 1 R	0.63	0.14	0.62	0.09	0.10	0.5	0.321	0.369	0.68	0.05	0.69	0.06	-0.04	-0.2	0.432	0.458
Salience Ventral Attention A: Parietal Operculum 1 R	0.60	0.10	0.61	0.07	-0.12	-0.6	0.288	0.347	0.69	0.05	0.67	0.04	0.24	1.1	0.132	0.191
Salience Ventral Attention A: Insula: 1 R	0.61	0.13	0.61	0.11	-0.02	-0.1	0.468	0.477	0.68	0.05	0.64	0.05	0.65	3.0	0.002	0.011
Salience Ventral Attention A: Parietal Medial 1 R	0.60	0.12	0.61	0.11	-0.04	-0.2	0.427	0.449	0.67	0.08	0.71	0.05	-0.58	-2.7	0.004	0.019
Salience Ventral Attention A: Frontal Medial 1 R	0.61	0.13	0.60	0.12	0.03	0.1	0.445	0.459	0.67	0.06	0.67	0.05	-0.06	-0.3	0.395	0.425
Salience Ventral Attention B: Inferior Parietal Lobule 1 R	0.58	0.10	0.59	0.07	-0.07	-0.3	0.376	0.417	0.63	0.08	0.61	0.06	0.34	1.6	0.061	0.120
Salience Ventral Attention B: Lateral Prefrontal Cortex 1 R	0.76	0.07	0.73	0.06	0.40	1.8	0.034	0.085	0.60	0.08	0.59	0.07	0.12	0.6	0.284	0.343
Salience Ventral Attention B: Medial Posterior Prefrontal 1 R	0.61	0.13	0.60	0.12	0.14	0.7	0.254	0.321	0.60	0.07	0.63	0.07	-0.55	-2.5	0.007	0.027
Limbic B: Orbital Frontal Cortex 0 R	0.71	0.11	0.71	0.07	-0.05	-0.2	0.408	0.446	0.57	0.13	0.53	0.11	0.33	1.5	0.064	0.123
Limbic A: Temporal Pole 1 R	0.61	0.10	0.58	0.14	0.24	1.1	0.130	0.200	0.62	0.06	0.57	0.09	0.65	3.0	0.002	0.011
Control A: Intraparietal Sulcus 1 R	0.58	0.16	0.62	0.06	-0.29	-1.3	0.093	0.177	0.64	0.05	0.64	0.06	0.09	0.4	0.335	0.377
Control A: Lateral Prefrontal Cortex 1 R	0.76	0.06	0.73	0.06	0.44	2.0	0.022	0.069	0.63	0.07	0.60	0.05	0.46	2.1	0.019	0.057
Control A: Lateral Prefrontal Cortex 2 R	0.73	0.09	0.69	0.07	0.50	2.3	0.012	0.054	0.69	0.06	0.65	0.06	0.72	3.4	0.001	0.006
Control B: Temporal 1 R	0.69	0.09	0.68	0.07	0.14	0.6	0.263	0.328	0.68	0.07	0.61	0.07	1.03	4.8	0.000	0.000
Control B: inferior parietal lobule 1 R	0.68	0.09	0.67	0.13	0.09	0.4	0.341	0.383	0.62	0.08	0.59	0.07	0.37	1.7	0.044	0.103
Control B: Lateral Prefrontal Cortexd 1 R	0.76	0.08	0.72	0.07	0.44	2.1	0.022	0.069	0.63	0.08	0.62	0.06	0.08	0.4	0.357	0.393
Control B: Lateral Prefrontal Cortexv 1 R	0.81	0.04	0.77	0.07	0.65	3.0	0.002	0.020	0.62	0.09	0.59	0.07	0.42	2.0	0.027	0.079
Control C: Cingulate Posterior 1 R	0.63	0.13	0.66	0.12	-0.24	-1.1	0.135	0.200	0.60	0.07	0.60	0.06	0.04	0.2	0.435	0.458
Control C: Precuneus 1 R	0.69	0.07	0.71	0.08	-0.37	-1.7	0.046	0.109	0.62	0.06	0.66	0.05	-0.71	-3.3	0.001	0.006
Default A: Inferior Parietal Lobule 1 R	0.59	0.10	0.62	0.11	-0.23	-1.1	0.140	0.203	0.64	0.07	0.62	0.06	0.29	1.4	0.090	0.156
Default A: Dorsal Prefrontal Cortex 1 R	0.71	0.13	0.67	0.09	0.32	1.5	0.072	0.156	0.62	0.08	0.61	0.07	0.10	0.5	0.317	0.369
Default A: Precuneus Posterior Cingulate Cortex 1 R	0.64	0.08	0.67	0.13	-0.28	-1.3	0.098	0.177	0.62	0.07	0.61	0.06	0.14	0.6	0.266	0.332
Default A: Medial Prefrontal Cortex 1 R	0.70	0.08	0.66	0.07	0.55	2.5	0.006	0.037	0.62	0.07	0.62	0.07	-0.01	-0.1	0.473	0.482
Default B: Dorsal Prefrontal Cortex 1 R	0.76	0.05	0.71	0.08	0.82	3.8	0.000	0.005	0.65	0.08	0.64	0.08	0.19	0.9	0.186	0.245
Default B: Ventral Prefrontal Cortex 1 R	0.65	0.08	0.65	0.06	-0.10	-0.5	0.318	0.369	0.63	0.06	0.61	0.07	0.34	1.6	0.060	0.120
Default B: Ventral Prefrontal Cortex 2 R	0.72	0.06	0.71	0.06	0.24	1.1	0.136	0.200	0.64	0.07	0.62	0.05	0.39	1.8	0.037	0.094
Default C: RetroSuperior Parietal Lobuleenial 1 R	0.54	0.12	0.59	0.12	-0.40	-1.9	0.033	0.085	0.61	0.05	0.62	0.06	-0.19	-0.9	0.196	0.254
Default C: Parahippocampal Cortex 1 R	0.49	0.15	0.53	0.13	-0.29	-1.3	0.092	0.177	0.66	0.07	0.63	0.06	0.50	2.3	0.012	0.043
Temporal Parietal 1 R	0.50	0.19	0.59	0.12	-0.54	-2.5	0.007	0.037	0.67	0.06	0.63	0.08	0.61	2.8	0.003	0.014
Temporal Parietal 2 R	0.61	0.14	0.63	0.07	-0.18	-0.8	0.201	0.268	0.67	0.06	0.64	0.06	0.42	1.9	0.029	0.079
Temporal Parietal 3 R	0.55	0.10	0.61	0.11	-0.54	-2.5	0.007	0.037	0.68	0.06	0.66	0.06	0.26	1.2	0.117	0.182

Supplementary Table 3. Mean, standard deviation and effect sizes (Cohen's D) of regional local efficiency for younger (N = 40) and older (N = 86) adults in fPET and fMRI, and t-tests of age group differences.

	fPET								fMRI							
	Younger		Older		Younger vs Older				Younger		Older		Younger vs Older			
	Mean	SD	Mean	SD	Cohen's D	*t*-value	p	p-FDR	Mean	SD	Mean	SD	Cohen's D	*t*-value	p	p-FDR
Visual Central: Extra Striate Cortex 1 L	0.71	0.22	0.69	0.17	0.11	0.5	0.312	0.389	0.81	0.06	0.84	0.06	-0.44	-2.0	0.022	0.150
Visual Central: Extra Striate Cortex 2 L	0.74	0.15	0.71	0.12	0.19	0.9	0.190	0.283	0.81	0.09	0.85	0.08	-0.46	-2.1	0.019	0.150
Visual Central: Striate Cortex 1 L	0.69	0.29	0.70	0.13	-0.08	-0.4	0.360	0.428	0.81	0.10	0.82	0.10	-0.17	-0.8	0.211	0.360
Visual Central: Extra Striate Cortex 3 L	0.70	0.21	0.70	0.14	0.00	0.0	0.497	0.497	0.80	0.05	0.82	0.05	-0.31	-1.4	0.078	0.280
Visual Peripheral: Extra Striate Inferior 1 L	0.63	0.30	0.66	0.19	-0.12	-0.5	0.297	0.376	0.82	0.04	0.83	0.05	-0.26	-1.2	0.119	0.280
Visual Peripheral: Striate Cortex Calcarine 1 L	0.68	0.25	0.63	0.23	0.22	1.0	0.152	0.242	0.81	0.05	0.81	0.05	0.03	0.2	0.436	0.474
Visual Peripheral: Extra Striate CortexSup 1 L	0.67	0.28	0.66	0.19	0.02	0.1	0.456	0.480	0.80	0.05	0.81	0.04	-0.24	-1.1	0.134	0.280
Somatomotor A: 1 L	0.77	0.17	0.68	0.15	0.53	2.4	0.008	0.031	0.79	0.15	0.82	0.05	-0.31	-1.4	0.076	0.280
Somatomotor A: 2 L	0.72	0.20	0.69	0.15	0.17	0.8	0.221	0.315	0.82	0.08	0.82	0.05	0.08	0.4	0.362	0.426
Somatomotor B: Auditory 1 L	0.75	0.17	0.74	0.13	0.06	0.3	0.388	0.446	0.77	0.11	0.79	0.06	-0.25	-1.2	0.125	0.280
Somatomotor B: S2 1 L	0.57	0.34	0.72	0.11	-0.63	-2.8	0.004	0.015	0.79	0.05	0.80	0.06	-0.30	-1.4	0.085	0.280
Somatomotor B: S2 2 L	0.68	0.19	0.69	0.19	-0.03	-0.1	0.451	0.480	0.78	0.06	0.82	0.06	-0.74	-3.4	0.000	0.023
Somatomotor B: Central 1 L	0.79	0.14	0.66	0.20	0.69	3.2	0.001	0.006	0.83	0.14	0.86	0.05	-0.31	-1.4	0.077	0.280
Dorsal Attention A: Temporal Occipital 1 L	0.62	0.27	0.71	0.12	-0.44	-2.0	0.023	0.059	0.76	0.04	0.78	0.05	-0.43	-2.0	0.025	0.156
Dorsal Attention A: Parietal Occipital 1 L	0.62	0.29	0.69	0.18	-0.26	-1.2	0.122	0.207	0.79	0.06	0.79	0.06	-0.09	-0.4	0.335	0.420
Dorsal Attention A: Superior Parietal Lobule 1 L	0.80	0.09	0.75	0.07	0.70	3.2	0.001	0.005	0.77	0.05	0.78	0.05	-0.28	-1.3	0.103	0.280
Dorsal Attention B: Post Central 1 L	0.76	0.21	0.67	0.21	0.41	1.9	0.032	0.070	0.77	0.14	0.81	0.05	-0.47	-2.2	0.016	0.150
Dorsal Attention B: Post Central 2 L	0.71	0.24	0.68	0.15	0.14	0.6	0.270	0.353	0.81	0.07	0.83	0.07	-0.24	-1.1	0.134	0.280
Dorsal Attention B: Post Central 3 L	0.72	0.25	0.70	0.16	0.11	0.5	0.315	0.389	0.80	0.07	0.80	0.06	-0.02	-0.1	0.458	0.486
Dorsal Attention B: Frontal Eye Fields 1 L	0.79	0.11	0.66	0.21	0.76	3.5	0.000	0.004	0.75	0.07	0.77	0.05	-0.31	-1.4	0.078	0.280
Salience Ventral Attention A: Parietal Operculum 1 L	0.80	0.12	0.73	0.12	0.59	2.7	0.004	0.016	0.77	0.09	0.76	0.13	0.13	0.6	0.273	0.386
Salience Ventral Attention A: Insula: 1 L	0.56	0.26	0.63	0.19	-0.29	-1.2	0.114	0.197	0.79	0.10	0.80	0.10	-0.17	-0.8	0.218	0.360
Salience Ventral Attention A: Insula: 2 L	0.67	0.25	0.71	0.14	-0.22	-1.0	0.162	0.253	0.77	0.07	0.77	0.09	-0.08	-0.4	0.351	0.426
Salience Ventral Attention A: Parietal Medial 1 L	0.79	0.14	0.74	0.11	0.42	1.9	0.032	0.070	0.74	0.07	0.77	0.05	-0.52	-2.4	0.009	0.097
Salience Ventral Attention A: Frontal Medial 1 L	0.78	0.18	0.70	0.17	0.47	2.1	0.018	0.050	0.78	0.08	0.78	0.07	0.01	0.1	0.473	0.488
Salience Ventral Attention B: Lateral Prefrontal Cortex 1 L	0.80	0.05	0.76	0.06	0.79	3.7	0.000	0.002	0.70	0.18	0.74	0.11	-0.25	-1.1	0.130	0.280
Salience Ventral Attention B: Medial Posterior Prefrontal 1 L	0.81	0.11	0.70	0.17	0.83	3.8	0.000	0.002	0.71	0.15	0.74	0.06	-0.30	-1.4	0.082	0.280
Limbic A Temporal Pole 1 L	0.74	0.10	0.74	0.12	0.04	0.2	0.434	0.480	0.78	0.10	0.77	0.16	0.12	0.6	0.289	0.391
Limbic A: Temporal Pole 2 L	0.70	0.18	0.62	0.20	0.41	1.8	0.038	0.077	0.80	0.08	0.75	0.19	0.35	1.6	0.053	0.250
Limbic B: Orbital Frontal Cortex 1 L	0.75	0.13	0.71	0.14	0.30	1.4	0.089	0.161	0.78	0.07	0.76	0.09	0.27	1.3	0.107	0.280
Control A: Intraparietal Sulcus 1 L	0.81	0.05	0.75	0.07	1.03	4.8	0.000	0.000	0.74	0.06	0.75	0.06	-0.15	-0.7	0.250	0.373
Control A: Lateral Prefrontal Cortex 1 L	0.79	0.04	0.75	0.06	0.71	3.3	0.001	0.005	0.73	0.09	0.75	0.07	-0.16	-0.7	0.235	0.362
Control A: Lateral Prefrontal Cortex 2 L	0.81	0.12	0.73	0.12	0.67	3.1	0.001	0.006	0.75	0.06	0.73	0.10	0.21	1.0	0.172	0.320
Control B: Lateral Prefrontal Cortexv 1 L	0.78	0.06	0.76	0.06	0.27	1.2	0.107	0.189	0.77	0.08	0.77	0.11	0.00	0.0	0.499	0.499
Control C: Precuneus 1 L	0.80	0.14	0.75	0.13	0.34	1.6	0.057	0.111	0.75	0.10	0.78	0.06	-0.41	-1.9	0.031	0.173
Control C: Precuneus 2 L	0.78	0.16	0.74	0.09	0.30	1.4	0.088	0.161	0.72	0.10	0.76	0.06	-0.52	-2.4	0.009	0.097
Control C: Cingulate Posterior 1 L	0.83	0.07	0.75	0.12	0.71	3.2	0.001	0.005	0.78	0.07	0.76	0.07	0.27	1.3	0.106	0.280
Default A: Dorsal Prefrontal Cortex 1 L	0.80	0.14	0.74	0.15	0.41	1.9	0.032	0.070	0.71	0.14	0.73	0.14	-0.18	-0.8	0.208	0.360
Default A: Precuneus Posterior Cingulate Cortex1 L	0.81	0.06	0.76	0.06	0.83	3.8	0.000	0.002	0.74	0.06	0.74	0.08	0.09	0.4	0.336	0.420
Default A: Medial Prefrontal Cortex 1 L	0.81	0.09	0.70	0.18	0.80	3.7	0.000	0.002	0.74	0.11	0.76	0.09	-0.13	-0.6	0.274	0.386
Default B: Temp 1 L	0.68	0.23	0.68	0.19	0.00	0.0	0.492	0.497	0.77	0.06	0.75	0.06	0.20	0.9	0.179	0.320
Default B: Temp 2 L	0.78	0.11	0.73	0.11	0.50	2.3	0.012	0.040	0.78	0.06	0.76	0.08	0.23	1.1	0.143	0.285
Default B: Inferior Parietal Lobule 1 L	0.79	0.06	0.73	0.10	0.63	2.9	0.002	0.010	0.75	0.04	0.74	0.04	0.36	1.7	0.049	0.247
Default B: Dorsal Prefrontal Cortex 1 L	0.78	0.05	0.73	0.09	0.69	3.2	0.001	0.006	0.76	0.06	0.74	0.09	0.24	1.1	0.133	0.280
Default B: Lateral Prefrontal Cortex 1 L	0.81	0.12	0.74	0.10	0.63	2.9	0.002	0.010	0.74	0.11	0.74	0.10	-0.04	-0.2	0.431	0.474
Default B: Ventral Prefrontal Cortex 1 L	0.79	0.10	0.75	0.13	0.41	1.9	0.032	0.070	0.76	0.08	0.75	0.07	0.16	0.8	0.225	0.360
Default B: Ventral Prefrontal Cortex 2 L	0.78	0.07	0.74	0.06	0.52	2.4	0.009	0.032	0.75	0.06	0.75	0.05	-0.16	-0.7	0.228	0.360
Default C: RetroSuperior Parietal Lobuleenial 1 L	0.67	0.30	0.70	0.13	-0.15	-0.7	0.249	0.341	0.75	0.07	0.78	0.11	-0.28	-1.3	0.097	0.280
Default C: Parahippocampal Cortex 1 L	0.60	0.31	0.52	0.24	0.29	1.2	0.108	0.189	0.77	0.16	0.82	0.09	-0.39	-1.8	0.040	0.209
Temporal Parietal 1 L	0.73	0.22	0.70	0.17	0.18	0.8	0.203	0.298	0.76	0.09	0.78	0.06	-0.27	-1.2	0.110	0.280
Visual Central: Extra Striate Cortex 1 R	0.73	0.16	0.73	0.08	0.02	0.1	0.456	0.480	0.79	0.05	0.83	0.04	-0.82	-3.8	0.000	0.013
Visual Central: Extra Striate Cortex 2 R	0.75	0.11	0.70	0.13	0.40	1.9	0.033	0.070	0.82	0.11	0.86	0.07	-0.42	-1.9	0.029	0.171
Visual Central: Extra Striate Cortex 3 R	0.76	0.17	0.72	0.17	0.23	1.0	0.149	0.242	0.79	0.07	0.84	0.05	-0.71	-3.3	0.001	0.023
Visual Peripheral: Striate Cortex Calcarine 1 R	0.70	0.22	0.70	0.14	0.04	0.2	0.431	0.480	0.83	0.08	0.85	0.05	-0.30	-1.4	0.087	0.280
Visual Peripheral: Extra Striate Inferior 1 R	0.68	0.23	0.68	0.14	-0.03	-0.1	0.453	0.480	0.79	0.04	0.81	0.05	-0.27	-1.3	0.105	0.280
Visual Peripheral: Extra Striate Superior 1 R	0.69	0.23	0.69	0.18	0.02	0.1	0.454	0.480	0.81	0.06	0.83	0.05	-0.26	-1.2	0.115	0.280
Somatomotor A: 1 R	0.62	0.30	0.64	0.21	-0.07	-0.3	0.375	0.436	0.81	0.10	0.81	0.06	-0.05	-0.2	0.414	0.470
Somatomotor A: 2 R	0.64	0.26	0.63	0.23	0.01	0.1	0.479	0.497	0.84	0.06	0.84	0.05	-0.04	-0.2	0.427	0.474
Somatomotor A: 3 R	0.74	0.20	0.65	0.22	0.45	2.0	0.025	0.061	0.81	0.05	0.82	0.05	-0.14	-0.6	0.263	0.382
Somatomotor A: 4 R	0.75	0.17	0.61	0.20	0.72	3.3	0.001	0.005	0.83	0.06	0.83	0.04	-0.03	-0.1	0.449	0.483
Somatomotor B: Auditory 1 R	0.78	0.11	0.76	0.07	0.13	0.6	0.272	0.353	0.80	0.06	0.80	0.06	-0.04	-0.2	0.431	0.474
Somatomotor B: S2 1 R	0.64	0.27	0.71	0.16	-0.33	-1.4	0.080	0.152	0.80	0.07	0.82	0.06	-0.28	-1.3	0.101	0.280
Somatomotor B: S2 2 R	0.73	0.24	0.76	0.12	-0.14	-0.7	0.257	0.342	0.81	0.07	0.82	0.07	-0.25	-1.2	0.124	0.280
Somatomotor B: Central 1 R	0.73	0.24	0.70	0.17	0.17	0.8	0.225	0.316	0.89	0.06	0.88	0.06	0.14	0.6	0.260	0.382
Dorsal Attention A: Temporal Occipital 1 R	0.65	0.25	0.68	0.15	-0.14	-0.7	0.256	0.342	0.77	0.06	0.80	0.05	-0.59	-2.7	0.004	0.059
Dorsal Attention A: Parietal Occipital 1 R	0.73	0.17	0.73	0.12	-0.01	0.0	0.485	0.497	0.75	0.06	0.76	0.05	-0.12	-0.6	0.286	0.391
Dorsal Attention A: Superior Parietal Lobule 1 R	0.75	0.18	0.75	0.09	0.01	0.0	0.490	0.497	0.75	0.09	0.79	0.05	-0.61	-2.8	0.003	0.059
Dorsal Attention B: Post Central 1 R	0.66	0.27	0.63	0.21	0.13	0.6	0.280	0.359	0.81	0.06	0.83	0.05	-0.23	-1.1	0.142	0.285
Dorsal Attention B: Post Central 2 R	0.77	0.17	0.70	0.16	0.38	1.8	0.041	0.083	0.78	0.06	0.78	0.05	-0.07	-0.3	0.372	0.432
Dorsal Attention B: Frontal Eye Fields 1 R	0.78	0.14	0.71	0.15	0.51	2.3	0.011	0.036	0.77	0.07	0.79	0.07	-0.20	-0.9	0.174	0.320
Salience Ventral Attention A: Parietal Operculum 1 R	0.76	0.20	0.69	0.12	0.40	1.8	0.036	0.075	0.80	0.06	0.80	0.06	-0.12	-0.5	0.296	0.395
Salience Ventral Attention A: Insula: 1 R	0.79	0.12	0.75	0.09	0.46	2.1	0.019	0.051	0.78	0.05	0.76	0.06	0.25	1.2	0.125	0.280
Salience Ventral Attention A: Parietal Medial 1 R	0.79	0.18	0.71	0.16	0.48	2.2	0.016	0.047	0.74	0.10	0.77	0.05	-0.46	-2.1	0.018	0.150
Salience Ventral Attention A: Frontal Medial 1 R	0.77	0.13	0.70	0.16	0.48	2.2	0.016	0.046	0.77	0.07	0.77	0.05	0.00	0.0	0.497	0.499
Salience Ventral Attention B: Inferior Parietal Lobule 1 R	0.72	0.25	0.66	0.21	0.23	1.0	0.150	0.242	0.74	0.10	0.72	0.11	0.17	0.8	0.219	0.360
Salience Ventral Attention B: Lateral Prefrontal Cortex 1 R	0.80	0.06	0.75	0.06	0.89	4.1	0.000	0.001	0.77	0.07	0.73	0.12	0.44	2.1	0.021	0.150
Salience Ventral Attention B: Medial Posterior Prefrontal 1 R	0.80	0.13	0.74	0.11	0.44	2.0	0.024	0.060	0.72	0.08	0.73	0.10	-0.11	-0.5	0.301	0.397
Limbic B: Orbital Frontal Cortex 0 R	0.73	0.15	0.76	0.07	-0.20	-0.9	0.185	0.282	0.77	0.12	0.75	0.18	0.16	0.7	0.230	0.360
Limbic A: Temporal Pole 1 R	0.70	0.25	0.66	0.17	0.20	0.9	0.186	0.282	0.80	0.08	0.76	0.16	0.28	1.3	0.102	0.280
Control A: Intraparietal Sulcus 1 R	0.73	0.21	0.72	0.11	0.08	0.4	0.352	0.424	0.77	0.05	0.77	0.06	-0.08	-0.4	0.360	0.426
Control A: Lateral Prefrontal Cortex 1 R	0.80	0.05	0.76	0.06	0.68	3.1	0.001	0.006	0.74	0.09	0.74	0.08	-0.07	-0.3	0.377	0.433
Control A: Lateral Prefrontal Cortex 2 R	0.80	0.05	0.77	0.07	0.49	2.3	0.013	0.040	0.76	0.06	0.75	0.05	0.15	0.7	0.242	0.367
Control B: Temporal 1 R	0.77	0.07	0.75	0.08	0.37	1.7	0.045	0.089	0.76	0.07	0.73	0.11	0.30	1.4	0.081	0.280
Control B: inferior parietal lobule 1 R	0.79	0.14	0.73	0.09	0.49	2.3	0.013	0.040	0.73	0.15	0.73	0.07	0.00	0.0	0.493	0.499
Control B: Lateral Prefrontal Cortexd 1 R	0.76	0.13	0.75	0.06	0.18	0.8	0.206	0.298	0.75	0.06	0.74	0.06	0.20	0.9	0.179	0.320
Control B: Lateral Prefrontal Cortexv 1 R	0.76	0.04	0.74	0.04	0.58	2.7	0.004	0.016	0.78	0.07	0.77	0.07	0.08	0.4	0.360	0.426
Control C: Cingulate Posterior 1 R	0.83	0.06	0.75	0.13	0.76	3.5	0.000	0.004	0.74	0.07	0.74	0.07	0.02	0.1	0.466	0.486
Control C: Precuneus 1 R	0.80	0.06	0.76	0.07	0.71	3.3	0.001	0.005	0.71	0.09	0.75	0.04	-0.69	-3.2	0.001	0.025
Default A: Inferior Parietal Lobule 1 R	0.74	0.18	0.73	0.13	0.09	0.4	0.342	0.417	0.74	0.10	0.74	0.07	0.09	0.4	0.343	0.424
Default A: Dorsal Prefrontal Cortex 1 R	0.81	0.04	0.73	0.14	0.73	3.4	0.001	0.005	0.75	0.11	0.74	0.08	0.11	0.5	0.314	0.403
Default A: Precuneus Posterior Cingulate Cortex 1 R	0.83	0.07	0.75	0.11	0.88	4.1	0.000	0.001	0.75	0.07	0.76	0.06	-0.20	-0.9	0.177	0.320
Default A: Medial Prefrontal Cortex 1 R	0.81	0.07	0.75	0.14	0.52	2.4	0.010	0.033	0.75	0.07	0.75	0.06	-0.10	-0.5	0.315	0.403
Default B: Dorsal Prefrontal Cortex 1 R	0.79	0.04	0.73	0.07	0.95	4.4	0.000	0.001	0.76	0.05	0.75	0.07	0.25	1.1	0.128	0.280
Default B: Ventral Prefrontal Cortex 1 R	0.83	0.07	0.75	0.11	0.85	3.9	0.000	0.002	0.77	0.07	0.76	0.06	0.12	0.6	0.288	0.391
Default B: Ventral Prefrontal Cortex 2 R	0.79	0.09	0.76	0.06	0.41	1.9	0.029	0.070	0.76	0.06	0.74	0.07	0.28	1.3	0.098	0.280
Default C: RetroSuperior Parietal Lobuleenial 1 R	0.67	0.26	0.69	0.19	-0.07	-0.3	0.375	0.436	0.77	0.06	0.80	0.07	-0.44	-2.0	0.023	0.150
Default C: Parahippocampal Cortex 1 R	0.63	0.25	0.64	0.20	-0.05	-0.2	0.413	0.470	0.75	0.06	0.79	0.07	-0.60	-2.8	0.004	0.059
Temporal Parietal 1 R	0.75	0.20	0.73	0.13	0.16	0.7	0.238	0.331	0.78	0.07	0.77	0.13	0.16	0.7	0.229	0.360
Temporal Parietal 2 R	0.79	0.12	0.74	0.11	0.45	2.1	0.021	0.055	0.78	0.05	0.76	0.10	0.22	1.0	0.161	0.316
Temporal Parietal 3 R	0.69	0.26	0.73	0.11	-0.25	-1.1	0.131	0.219	0.79	0.05	0.79	0.05	0.02	0.1	0.463	0.486

Supplementary Table 4 Mean, standard deviation and effect sizes (Cohen's D) of regional betweenness centrality for younger (N = 40) and older (N = 46) adults, and t-tests of age group differences.

	fPET								fMRI							
	Younger		Older		Younger vs Older				Younger		Older		Younger vs Older			
	Mean	SD	Mean	SD	Cohen's D	*t* -value	p	p-FDR	Mean	SD	Mean	SD	Cohen's D	*t* -value	p	p-FDR
Visual Central: Extra Striate Cortex 1 L	0.008	0.012	0.010	0.008	-0.26	-1.2	0.115	0.244	0.006	0.007	0.005	0.006	0.19	0.9	0.189	0.411
Visual Central: Extra Striate Cortex 2 L	0.009	0.008	0.014	0.012	-0.52	-2.4	0.009	0.072	0.004	0.005	0.004	0.005	-0.01	0.0	0.483	0.494
Visual Central: Striate Cortex 1 L	0.005	0.005	0.009	0.010	-0.51	-2.4	0.010	0.072	0.006	0.009	0.006	0.006	0.00	0.0	0.494	0.495
Visual Central: Extra Striate Cortex 3 L	0.006	0.006	0.009	0.005	-0.46	-2.1	0.019	0.097	0.006	0.005	0.007	0.004	-0.22	-1.0	0.157	0.397
Visual Peripheral: Extra Striate Inferior 1 L	0.004	0.005	0.004	0.004	0.05	0.2	0.417	0.465	0.007	0.006	0.006	0.004	0.13	0.6	0.276	0.417
Visual Peripheral: Striate Cortex Calcarine 1 L	0.008	0.010	0.006	0.005	0.32	1.5	0.069	0.185	0.006	0.004	0.007	0.005	-0.35	-1.6	0.055	0.306
Visual Peripheral: Extra Striate CortexSup 1 L	0.005	0.006	0.005	0.005	0.06	0.3	0.389	0.450	0.007	0.005	0.007	0.004	-0.09	-0.4	0.334	0.432
Somatomotor A: 1 L	0.007	0.007	0.009	0.011	-0.32	-1.5	0.070	0.185	0.006	0.005	0.007	0.005	-0.31	-1.4	0.077	0.345
Somatomotor A: 2 L	0.009	0.008	0.009	0.007	0.04	0.2	0.419	0.465	0.005	0.006	0.008	0.005	-0.64	-3.0	0.002	0.095
Somatomotor B: Auditory 1 L	0.013	0.009	0.012	0.007	0.16	0.7	0.231	0.335	0.008	0.005	0.009	0.006	-0.17	-0.8	0.212	0.411
Somatomotor B: S2 1 L	0.002	0.002	0.003	0.003	-0.51	-2.4	0.010	0.072	0.010	0.006	0.007	0.005	0.49	2.3	0.013	0.142
Somatomotor B: S2 2 L	0.005	0.005	0.007	0.005	-0.55	-2.6	0.006	0.068	0.009	0.005	0.007	0.004	0.57	2.6	0.005	0.097
Somatomotor B: Central 1 L	0.004	0.006	0.006	0.005	-0.26	-1.2	0.114	0.244	0.003	0.004	0.003	0.003	-0.15	-0.7	0.251	0.411
Dorsal Attention A: Temporal Occipital 1 L	0.010	0.007	0.007	0.006	0.35	1.6	0.057	0.175	0.014	0.008	0.012	0.006	0.39	1.8	0.036	0.301
Dorsal Attention A: Parietal Occipital 1 L	0.005	0.006	0.005	0.004	-0.01	0.0	0.490	0.495	0.012	0.007	0.011	0.008	0.10	0.5	0.320	0.432
Dorsal Attention A: Superior Parietal Lobule 1 L	0.009	0.008	0.010	0.006	-0.10	-0.5	0.315	0.396	0.010	0.006	0.011	0.006	-0.29	-1.3	0.090	0.348
Dorsal Attention B: Post Central 1 L	0.005	0.006	0.005	0.005	0.02	0.1	0.470	0.495	0.009	0.007	0.007	0.004	0.35	1.6	0.053	0.306
Dorsal Attention B: Post Central 2 L	0.003	0.004	0.005	0.008	-0.39	-1.8	0.037	0.131	0.005	0.004	0.006	0.004	-0.18	-0.8	0.205	0.411
Dorsal Attention B: Post Central 3 L	0.005	0.005	0.005	0.005	-0.07	-0.3	0.376	0.447	0.008	0.005	0.011	0.007	-0.38	-1.8	0.040	0.306
Dorsal Attention B: Frontal Eye Fields 1 L	0.008	0.008	0.005	0.005	0.45	2.1	0.019	0.097	0.012	0.008	0.014	0.011	-0.13	-0.6	0.268	0.417
Salience Ventral Attention A: Parietal Operculum 1 L	0.007	0.006	0.005	0.005	0.30	1.4	0.085	0.196	0.009	0.006	0.009	0.005	0.07	0.3	0.374	0.432
Salience Ventral Attention A: Insula: 1 L	0.001	0.002	0.002	0.003	-0.38	-1.8	0.040	0.139	0.005	0.005	0.005	0.006	0.03	0.2	0.440	0.473
Salience Ventral Attention A: Insula: 2 L	0.005	0.007	0.005	0.004	0.12	0.5	0.293	0.386	0.008	0.005	0.007	0.006	0.14	0.7	0.256	0.411
Salience Ventral Attention A: Parietal Medial 1 L	0.004	0.007	0.005	0.004	-0.12	-0.6	0.284	0.378	0.013	0.009	0.016	0.009	-0.28	-1.3	0.102	0.363
Salience Ventral Attention A: Frontal Medial 1 L	0.005	0.008	0.006	0.004	-0.03	-0.2	0.439	0.483	0.007	0.007	0.009	0.008	-0.25	-1.1	0.127	0.373
Salience Ventral Attention B: Lateral Prefrontal Cortex 1 L	0.013	0.010	0.010	0.006	0.40	1.9	0.034	0.129	0.005	0.005	0.009	0.008	-0.55	-2.5	0.007	0.097
Salience Ventral Attention B: Medial Posterior Prefrontal 1 L	0.006	0.006	0.004	0.004	0.23	1.1	0.147	0.266	0.009	0.008	0.013	0.013	-0.31	-1.4	0.080	0.345
Limbic A Temporal Pole 1 L	0.014	0.012	0.009	0.006	0.57	2.6	0.005	0.068	0.004	0.003	0.003	0.004	0.43	2.0	0.026	0.236
Limbic A: Temporal Pole 2 L	0.004	0.005	0.003	0.003	0.24	1.1	0.138	0.263	0.006	0.005	0.005	0.005	0.14	0.7	0.252	0.411
Limbic B: Orbital Frontal Cortex 1 L	0.011	0.007	0.006	0.006	0.69	3.2	0.001	0.049	0.007	0.005	0.006	0.006	0.23	1.1	0.143	0.397
Control A: Intraparietal Sulcus 1 L	0.011	0.009	0.009	0.007	0.25	1.2	0.124	0.252	0.008	0.006	0.009	0.006	-0.08	-0.4	0.355	0.432
Control A: Lateral Prefrontal Cortex 1 L	0.017	0.008	0.014	0.009	0.34	1.6	0.058	0.175	0.005	0.004	0.007	0.006	-0.27	-1.2	0.109	0.363
Control A: Lateral Prefrontal Cortex 2 L	0.006	0.007	0.007	0.009	-0.14	-0.6	0.259	0.359	0.009	0.006	0.007	0.005	0.36	1.7	0.051	0.306
Control B: Lateral Prefrontal Cortexv 1 L	0.019	0.012	0.014	0.010	0.49	2.3	0.013	0.084	0.008	0.006	0.007	0.006	0.07	0.3	0.366	0.432
Control C: Precuneus 1 L	0.008	0.006	0.010	0.007	-0.22	-1.0	0.159	0.270	0.005	0.004	0.006	0.005	-0.08	-0.4	0.358	0.432
Control C: Precuneus 2 L	0.007	0.008	0.007	0.005	0.06	0.3	0.386	0.450	0.008	0.006	0.011	0.008	-0.55	-2.5	0.006	0.097
Control C: Cingulate Posterior 1 L	0.005	0.004	0.006	0.004	-0.34	-1.6	0.061	0.176	0.005	0.005	0.006	0.005	-0.19	-0.9	0.195	0.411
Default A: Dorsal Prefrontal Cortex 1 L	0.007	0.004	0.005	0.005	0.27	1.2	0.108	0.240	0.006	0.004	0.007	0.005	-0.15	-0.7	0.240	0.411
Default A: Precuneus Posterior Cingulate Cortex1 L	0.011	0.010	0.013	0.009	-0.22	-1.0	0.156	0.269	0.009	0.005	0.008	0.004	0.16	0.7	0.235	0.411
Default A: Medial Prefrontal Cortex 1 L	0.008	0.008	0.006	0.005	0.31	1.5	0.075	0.187	0.009	0.006	0.007	0.005	0.30	1.4	0.083	0.345
Default B: Temp 1 L	0.006	0.007	0.005	0.004	0.26	1.2	0.117	0.244	0.012	0.007	0.011	0.008	0.07	0.3	0.380	0.432
Default B: Temp 2 L	0.005	0.004	0.006	0.006	-0.10	-0.5	0.327	0.403	0.009	0.006	0.008	0.006	0.12	0.6	0.285	0.417
Default B: Inferior Parietal Lobule 1 L	0.015	0.012	0.011	0.007	0.42	1.9	0.028	0.116	0.012	0.009	0.009	0.008	0.32	1.5	0.069	0.344
Default B: Dorsal Prefrontal Cortex 1 L	0.020	0.011	0.011	0.008	0.94	4.3	0.000	0.002	0.010	0.007	0.011	0.007	-0.12	-0.6	0.288	0.417
Default B: Lateral Prefrontal Cortex 1 L	0.008	0.007	0.008	0.007	0.01	0.1	0.477	0.495	0.008	0.006	0.008	0.004	-0.16	-0.7	0.235	0.411
Default B: Ventral Prefrontal Cortex 1 L	0.004	0.004	0.004	0.005	-0.16	-0.7	0.229	0.335	0.008	0.005	0.008	0.008	-0.07	-0.3	0.375	0.432
Default B: Ventral Prefrontal Cortex 2 L	0.018	0.011	0.016	0.010	0.17	0.8	0.211	0.324	0.009	0.006	0.007	0.005	0.30	1.4	0.081	0.345
Default C: RetroSuperior Parietal Lobuleenial 1 L	0.004	0.007	0.005	0.004	-0.14	-0.6	0.264	0.361	0.008	0.007	0.006	0.004	0.27	1.3	0.107	0.363
Default C: Parahippocampal Cortex 1 L	0.001	0.002	0.003	0.004	-0.56	-2.6	0.006	0.068	0.006	0.006	0.006	0.007	0.00	0.0	0.495	0.495
Temporal Parietal 1 L	0.004	0.005	0.006	0.006	-0.48	-2.2	0.015	0.093	0.009	0.007	0.010	0.007	-0.10	-0.5	0.326	0.432
Visual Central: Extra Striate Cortex 1 R	0.006	0.007	0.007	0.005	-0.10	-0.5	0.317	0.396	0.007	0.007	0.005	0.003	0.25	1.1	0.127	0.373
Visual Central: Extra Striate Cortex 2 R	0.013	0.011	0.015	0.011	-0.24	-1.1	0.139	0.263	0.003	0.003	0.003	0.003	-0.01	0.0	0.484	0.494
Visual Central: Extra Striate Cortex 3 R	0.006	0.010	0.009	0.007	-0.40	-1.8	0.035	0.130	0.005	0.004	0.005	0.003	0.16	0.7	0.232	0.411
Visual Peripheral: Striate Cortex Calcarine 1 R	0.005	0.005	0.007	0.007	-0.22	-1.0	0.153	0.269	0.004	0.004	0.005	0.004	-0.22	-1.0	0.159	0.397
Visual Peripheral: Extra Striate Inferior 1 R	0.004	0.006	0.005	0.004	-0.02	-0.1	0.472	0.495	0.009	0.007	0.009	0.007	-0.06	-0.3	0.398	0.435
Visual Peripheral: Extra Striate Superior 1 R	0.005	0.006	0.005	0.005	0.11	0.5	0.311	0.396	0.006	0.004	0.006	0.005	-0.16	-0.7	0.231	0.411
Somatomotor A: 1 R	0.003	0.005	0.004	0.004	-0.15	-0.7	0.241	0.344	0.008	0.008	0.009	0.006	-0.13	-0.6	0.275	0.417
Somatomotor A: 2 R	0.004	0.005	0.006	0.005	-0.46	-2.1	0.017	0.097	0.004	0.004	0.005	0.003	-0.08	-0.4	0.357	0.432
Somatomotor A: 3 R	0.003	0.004	0.003	0.003	-0.14	-0.6	0.259	0.359	0.007	0.005	0.007	0.004	-0.02	-0.1	0.465	0.485
Somatomotor A: 4 R	0.005	0.005	0.007	0.006	-0.33	-1.5	0.066	0.184	0.007	0.007	0.006	0.003	0.20	0.9	0.184	0.411
Somatomotor B: Auditory 1 R	0.014	0.011	0.013	0.008	0.13	0.6	0.277	0.375	0.010	0.009	0.009	0.006	0.12	0.6	0.285	0.417
Somatomotor B: S2 1 R	0.002	0.002	0.003	0.004	-0.45	-2.1	0.019	0.097	0.009	0.008	0.007	0.005	0.33	1.5	0.066	0.344
Somatomotor B: S2 2 R	0.005	0.007	0.004	0.004	0.01	0.0	0.486	0.495	0.007	0.005	0.007	0.005	0.03	0.1	0.448	0.477
Somatomotor B: Central 1 R	0.004	0.004	0.005	0.004	-0.19	-0.9	0.196	0.311	0.002	0.002	0.002	0.002	-0.18	-0.8	0.204	0.411
Dorsal Attention A: Temporal Occipital 1 R	0.006	0.006	0.007	0.006	-0.11	-0.5	0.313	0.396	0.014	0.009	0.009	0.006	0.57	2.6	0.005	0.097
Dorsal Attention A: Parietal Occipital 1 R	0.009	0.009	0.007	0.006	0.24	1.1	0.133	0.263	0.014	0.007	0.011	0.006	0.46	2.1	0.018	0.180
Dorsal Attention A: Superior Parietal Lobule 1 R	0.008	0.010	0.008	0.005	0.03	0.1	0.454	0.493	0.009	0.006	0.009	0.004	0.06	0.3	0.391	0.435
Dorsal Attention B: Post Central 1 R	0.003	0.005	0.004	0.004	-0.24	-1.1	0.140	0.263	0.007	0.004	0.006	0.004	0.14	0.7	0.254	0.411
Dorsal Attention B: Post Central 2 R	0.005	0.009	0.005	0.004	0.09	0.4	0.341	0.416	0.011	0.006	0.012	0.006	-0.30	-1.4	0.086	0.345
Dorsal Attention B: Frontal Eye Fields 1 R	0.007	0.007	0.006	0.006	0.17	0.8	0.220	0.333	0.011	0.006	0.012	0.008	-0.07	-0.3	0.372	0.432
Salience Ventral Attention A: Parietal Operculum 1 R	0.005	0.006	0.007	0.006	-0.30	-1.4	0.086	0.196	0.009	0.006	0.008	0.005	0.06	0.3	0.388	0.435
Salience Ventral Attention A: Insula: 1 R	0.006	0.007	0.006	0.005	-0.06	-0.3	0.394	0.450	0.011	0.006	0.010	0.006	0.14	0.7	0.259	0.411
Salience Ventral Attention A: Parietal Medial 1 R	0.004	0.004	0.006	0.005	-0.40	-1.9	0.033	0.129	0.013	0.009	0.014	0.007	-0.11	-0.5	0.311	0.432
Salience Ventral Attention A: Frontal Medial 1 R	0.005	0.005	0.005	0.004	0.06	0.3	0.396	0.450	0.012	0.008	0.012	0.008	0.06	0.3	0.400	0.435
Salience Ventral Attention B: Inferior Parietal Lobule 1 R	0.006	0.008	0.004	0.003	0.36	1.7	0.048	0.159	0.008	0.006	0.007	0.004	0.16	0.7	0.232	0.411
Salience Ventral Attention B: Lateral Prefrontal Cortex 1 R	0.016	0.012	0.014	0.008	0.20	0.9	0.183	0.300	0.005	0.004	0.006	0.006	-0.17	-0.8	0.216	0.411
Salience Ventral Attention B: Medial Posterior Prefrontal 1 R	0.006	0.007	0.004	0.005	0.21	1.0	0.171	0.285	0.007	0.005	0.011	0.008	-0.57	-2.6	0.005	0.097
Limbic B: Orbital Frontal Cortex 0 R	0.017	0.013	0.013	0.008	0.36	1.7	0.050	0.163	0.003	0.003	0.004	0.005	-0.26	-1.2	0.121	0.373
Limbic A: Temporal Pole 1 R	0.006	0.005	0.005	0.005	0.19	0.9	0.189	0.305	0.005	0.004	0.007	0.009	-0.24	-1.1	0.138	0.394
Control A: Intraparietal Sulcus 1 R	0.006	0.007	0.007	0.005	-0.01	-0.1	0.473	0.495	0.009	0.006	0.008	0.005	0.18	0.8	0.202	0.411
Control A: Lateral Prefrontal Cortex 1 R	0.018	0.011	0.013	0.007	0.61	2.8	0.003	0.068	0.007	0.004	0.007	0.006	-0.09	-0.4	0.338	0.432
Control A: Lateral Prefrontal Cortex 2 R	0.014	0.008	0.009	0.008	0.64	3.0	0.002	0.066	0.012	0.007	0.011	0.007	0.15	0.7	0.250	0.411
Control B: Temporal 1 R	0.013	0.009	0.010	0.008	0.32	1.5	0.072	0.186	0.012	0.007	0.010	0.010	0.22	1.0	0.156	0.397
Control B: inferior parietal lobule 1 R	0.011	0.009	0.011	0.007	0.01	0.0	0.484	0.495	0.007	0.004	0.006	0.004	0.15	0.7	0.249	0.411
Control B: Lateral Prefrontal Cortexd 1 R	0.021	0.012	0.015	0.008	0.57	2.6	0.005	0.068	0.007	0.004	0.011	0.009	-0.49	-2.3	0.013	0.142
Control B: Lateral Prefrontal Cortexv 1 R	0.029	0.014	0.022	0.011	0.57	2.6	0.005	0.068	0.006	0.004	0.007	0.005	-0.12	-0.5	0.293	0.418
Control C: Cingulate Posterior 1 R	0.007	0.008	0.008	0.007	-0.22	-1.0	0.155	0.269	0.007	0.005	0.007	0.005	-0.02	-0.1	0.464	0.485
Control C: Precuneus 1 R	0.011	0.009	0.014	0.009	-0.30	-1.4	0.082	0.195	0.012	0.008	0.013	0.007	-0.08	-0.4	0.355	0.432
Default A: Inferior Parietal Lobule 1 R	0.005	0.006	0.005	0.004	0.00	0.0	0.496	0.496	0.008	0.007	0.009	0.006	-0.07	-0.3	0.374	0.432
Default A: Dorsal Prefrontal Cortex 1 R	0.012	0.009	0.009	0.006	0.42	2.0	0.027	0.116	0.006	0.004	0.007	0.004	-0.25	-1.2	0.125	0.373
Default A: Precuneus Posterior Cingulate Cortex 1 R	0.007	0.007	0.009	0.006	-0.43	-2.0	0.026	0.116	0.008	0.005	0.007	0.005	0.11	0.5	0.307	0.432
Default A: Medial Prefrontal Cortex 1 R	0.011	0.009	0.007	0.004	0.52	2.4	0.009	0.072	0.008	0.004	0.010	0.007	-0.37	-1.7	0.046	0.306
Default B: Dorsal Prefrontal Cortex 1 R	0.016	0.008	0.014	0.009	0.31	1.4	0.078	0.191	0.009	0.005	0.011	0.008	-0.28	-1.3	0.102	0.363
Default B: Ventral Prefrontal Cortex 1 R	0.006	0.005	0.008	0.008	-0.34	-1.6	0.062	0.176	0.008	0.006	0.009	0.009	-0.10	-0.5	0.320	0.432
Default B: Ventral Prefrontal Cortex 2 R	0.012	0.011	0.011	0.007	0.16	0.7	0.230	0.335	0.007	0.005	0.009	0.006	-0.35	-1.6	0.052	0.306
Default C: RetroSuperior Parietal Lobuleenial 1 R	0.003	0.004	0.005	0.004	-0.52	-2.4	0.009	0.072	0.007	0.007	0.007	0.006	0.07	0.3	0.376	0.432
Default C: Parahippocampal Cortex 1 R	0.002	0.002	0.002	0.003	-0.18	-0.8	0.201	0.314	0.014	0.009	0.008	0.007	0.70	3.2	0.001	0.088
Temporal Parietal 1 R	0.003	0.003	0.004	0.004	-0.42	-1.9	0.028	0.116	0.010	0.010	0.009	0.007	0.14	0.7	0.256	0.411
Temporal Parietal 2 R	0.007	0.007	0.006	0.005	0.08	0.4	0.358	0.431	0.010	0.006	0.008	0.006	0.22	1.0	0.152	0.397
Temporal Parietal 3 R	0.004	0.006	0.005	0.005	-0.23	-1.1	0.145	0.266	0.008	0.005	0.008	0.005	0.14	0.7	0.252	0.411

Supplementary Table 5. Mean, standard deviation and effect sizes (Cohen's D) of regional degree for younger (N = 40) and older (N = 46) adults in fPET and fMRI, and t-tests of age group differences.

	fPET								fMRI							
	Younger		Older		Younger vs Older				Younger		Older		Younger vs Older			
	Mean	SD	Mean	SD	Cohen's D	*t*-value	p	p-FDR	Mean	SD	Mean	SD	Cohen's D	*t*-value	p	p-FDR
Visual Central: Extra Striate Cortex 1 L	25.4	17.0	34.0	16.6	-0.51	-2.4	0.010	0.038	26.4	11.0	28.5	8.3	-0.21	-1.0	0.164	0.231
Visual Central: Extra Striate Cortex 2 L	30.0	18.6	40.3	18.3	-0.56	-2.6	0.006	0.027	21.2	11.2	25.4	9.5	-0.41	-1.9	0.030	0.071
Visual Central: Striate Cortex 1 L	24.1	18.2	29.7	16.0	-0.33	-1.5	0.066	0.126	20.4	8.8	24.7	9.1	-0.48	-2.2	0.014	0.047
Visual Central: Extra Striate Cortex 3 L	23.6	16.6	32.5	14.3	-0.58	-2.7	0.005	0.024	26.1	7.5	34.0	8.4	-0.98	-4.5	0.000	0.000
Visual Peripheral: Extra Striate Inferior 1 L	15.5	11.0	19.6	11.2	-0.37	-1.7	0.045	0.116	29.9	11.8	33.5	10.2	-0.33	-1.5	0.064	0.123
Visual Peripheral: Striate Cortex Calcarine 1 L	24.2	15.2	21.9	12.7	0.16	0.8	0.225	0.308	26.4	10.7	33.7	10.8	-0.68	-3.1	0.001	0.007
Visual Peripheral: Extra Striate CortexSup 1 L	22.6	15.3	22.3	11.5	0.02	0.1	0.462	0.481	28.8	10.6	36.2	8.8	-0.76	-3.5	0.000	0.004
Somatomotor A: 1 L	31.4	17.3	31.0	16.8	0.02	0.1	0.455	0.479	32.2	13.3	38.2	9.3	-0.53	-2.4	0.008	0.033
Somatomotor A: 2 L	33.5	19.4	32.2	16.4	0.07	0.3	0.366	0.419	32.1	11.8	40.3	8.4	-0.81	-3.7	0.000	0.003
Somatomotor B: Auditory 1 L	40.5	15.0	40.2	14.7	0.02	0.1	0.471	0.486	33.9	13.1	35.9	12.1	-0.16	-0.7	0.234	0.287
Somatomotor B: S2 1 L	10.5	9.6	18.9	10.5	-0.84	-3.9	0.000	0.002	36.1	11.7	33.3	12.8	0.23	1.0	0.149	0.216
Somatomotor B: S2 2 L	21.6	15.7	29.2	13.5	-0.52	-2.4	0.009	0.037	37.2	11.8	34.6	9.2	0.25	1.1	0.129	0.198
Somatomotor B: Central 1 L	22.5	16.4	25.5	15.5	-0.19	-0.9	0.192	0.285	22.0	13.1	29.5	11.5	-0.62	-2.8	0.003	0.013
Dorsal Attention A: Temporal Occipital 1 L	25.4	17.4	28.1	13.0	-0.18	-0.8	0.208	0.292	38.2	11.3	35.2	8.5	0.30	1.4	0.085	0.149
Dorsal Attention A: Parietal Occipital 1 L	17.1	13.5	22.2	11.8	-0.41	-1.9	0.032	0.083	39.7	11.0	37.0	13.6	0.21	1.0	0.164	0.231
Dorsal Attention A: Superior Parietal Lobule 1 L	37.2	13.3	36.0	13.5	0.09	0.4	0.343	0.408	32.6	9.0	37.5	9.0	-0.54	-2.5	0.007	0.027
Dorsal Attention B: Post Central 1 L	24.7	14.9	21.2	13.1	0.25	1.1	0.129	0.212	35.3	12.1	38.2	7.1	-0.30	-1.4	0.084	0.149
Dorsal Attention B: Post Central 2 L	17.7	13.7	20.4	12.4	-0.21	-1.0	0.165	0.253	31.1	13.1	34.8	8.4	-0.34	-1.6	0.061	0.120
Dorsal Attention B: Post Central 3 L	27.8	15.8	24.2	12.8	0.25	1.2	0.124	0.207	35.2	9.6	41.1	8.5	-0.66	-3.0	0.002	0.009
Dorsal Attention B: Frontal Eye Fields 1 L	36.4	17.3	26.2	15.7	0.62	2.9	0.002	0.015	35.5	12.9	37.5	11.4	-0.17	-0.8	0.222	0.284
Salience Ventral Attention A: Parietal Operculum 1 L	30.8	14.2	26.3	13.1	0.33	1.5	0.065	0.126	35.6	10.0	32.2	10.5	0.33	1.5	0.068	0.128
Salience Ventral Attention A: Insula: 1 L	6.8	6.1	12.6	9.1	-0.74	-3.4	0.000	0.005	28.1	12.2	23.7	11.3	0.37	1.7	0.043	0.090
Salience Ventral Attention A: Insula: 2 L	19.7	15.4	22.3	12.1	-0.19	-0.9	0.194	0.285	30.6	10.4	25.8	9.4	0.48	2.2	0.014	0.047
Salience Ventral Attention A: Parietal Medial 1 L	21.4	13.5	23.5	10.1	-0.18	-0.8	0.203	0.290	34.6	13.7	42.7	11.6	-0.64	-3.0	0.002	0.010
Salience Ventral Attention A: Frontal Medial 1 L	27.5	16.4	25.5	13.1	0.13	0.6	0.273	0.354	27.8	13.8	29.8	11.2	-0.16	-0.7	0.231	0.287
Salience Ventral Attention B: Lateral Prefrontal Cortex 1 L	48.0	12.6	39.5	12.9	0.66	3.1	0.001	0.010	19.9	10.1	24.8	12.1	-0.44	-2.0	0.023	0.063
Salience Ventral Attention B: Medial Posterior Prefrontal 1 L	29.1	14.2	22.8	13.4	0.46	2.1	0.019	0.059	25.8	13.2	28.8	11.8	-0.24	-1.1	0.138	0.206
Limbic A Temporal Pole 1 L	38.0	21.4	35.2	14.4	0.15	0.7	0.240	0.320	23.0	11.5	14.0	11.2	0.79	3.7	0.000	0.003
Limbic A: Temporal Pole 2 L	16.4	13.4	15.9	11.8	0.04	0.2	0.422	0.464	24.5	12.6	18.6	10.9	0.51	2.3	0.011	0.040
Limbic B: Orbital Frontal Cortex 1 L	35.7	16.5	27.3	14.4	0.54	2.5	0.007	0.033	27.8	11.8	19.6	10.3	0.74	3.4	0.000	0.004
Control A: Intraparietal Sulcus 1 L	40.8	12.8	36.3	11.6	0.37	1.7	0.047	0.117	26.1	9.2	26.6	10.2	-0.05	-0.3	0.400	0.417
Control A: Lateral Prefrontal Cortex 1 L	53.7	8.5	46.2	13.4	0.66	3.0	0.002	0.010	21.8	8.8	22.2	8.8	-0.05	-0.2	0.414	0.421
Control A: Lateral Prefrontal Cortex 2 L	30.7	15.6	25.9	13.4	0.33	1.5	0.064	0.126	29.7	11.2	24.9	9.8	0.46	2.1	0.019	0.056
Control B: Lateral Prefrontal Cortexv 1 L	52.5	13.9	46.0	12.4	0.50	2.3	0.012	0.043	27.3	13.9	23.5	11.4	0.30	1.4	0.088	0.149
Control C: Precuneus 1 L	34.7	11.8	36.1	12.4	-0.11	-0.5	0.300	0.375	19.7	9.0	23.6	9.9	-0.41	-1.9	0.030	0.071
Control C: Precuneus 2 L	26.0	12.5	28.4	13.5	-0.18	-0.8	0.202	0.290	24.6	11.3	34.3	11.4	-0.86	-4.0	0.000	0.002
Control C: Cingulate Posterior 1 L	25.5	12.6	30.0	12.4	-0.36	-1.7	0.050	0.120	21.1	11.0	22.2	8.9	-0.11	-0.5	0.308	0.363
Default A: Dorsal Prefrontal Cortex 1 L	40.2	15.8	26.6	12.9	0.95	4.4	0.000	0.001	23.4	10.5	23.8	12.5	-0.04	-0.2	0.429	0.429
Default A: Precuneus Posterior Cingulate Cortex1 L	39.9	13.9	43.9	12.2	-0.31	-1.4	0.079	0.146	27.1	10.3	25.3	8.7	0.18	0.9	0.198	0.261
Default A: Medial Prefrontal Cortex 1 L	36.2	13.1	26.8	12.6	0.74	3.4	0.001	0.005	28.0	10.7	25.4	10.0	0.25	1.2	0.125	0.196
Default B: Temp 1 L	24.1	18.2	23.2	12.9	0.06	0.3	0.397	0.441	32.6	11.4	28.9	10.2	0.34	1.6	0.060	0.120
Default B: Temp 2 L	25.6	14.3	25.7	13.0	-0.01	0.0	0.485	0.489	31.1	11.8	26.4	9.9	0.43	2.0	0.024	0.065
Default B: Inferior Parietal Lobule 1 L	44.9	14.9	40.0	13.7	0.34	1.6	0.061	0.126	33.0	11.1	25.3	10.2	0.73	3.4	0.001	0.005
Default B: Dorsal Prefrontal Cortex 1 L	52.5	10.9	38.3	16.1	1.03	4.8	0.000	0.000	31.1	11.4	30.2	11.4	0.07	0.3	0.368	0.402
Default B: Lateral Prefrontal Cortex 1 L	38.3	14.7	33.3	14.3	0.34	1.6	0.059	0.125	27.1	12.4	26.5	10.2	0.05	0.2	0.407	0.419
Default B: Ventral Prefrontal Cortex 1 L	23.1	13.2	22.7	11.7	0.03	0.1	0.444	0.479	26.8	9.8	24.0	10.7	0.27	1.3	0.107	0.175
Default B: Ventral Prefrontal Cortex 2 L	51.5	15.5	46.1	15.1	0.35	1.6	0.054	0.122	28.0	10.7	24.1	8.8	0.40	1.8	0.035	0.075
Default C: RetroSuperior Parietal Lobuleenial 1 L	17.8	14.4	23.7	11.9	-0.45	-2.1	0.020	0.060	24.2	9.3	25.2	8.1	-0.11	-0.5	0.307	0.363
Default C: Parahippocampal Cortex 1 L	6.7	6.3	13.1	9.7	-0.78	-3.6	0.000	0.005	22.8	11.5	21.8	11.1	0.09	0.4	0.341	0.388
Temporal Parietal 1 L	19.3	14.0	24.0	12.2	-0.36	-1.7	0.051	0.120	32.1	12.1	33.8	11.7	-0.15	-0.7	0.250	0.301
Visual Central: Extra Striate Cortex 1 R	26.2	15.7	28.4	13.5	-0.15	-0.7	0.246	0.324	25.3	7.8	31.5	8.8	-0.75	-3.5	0.000	0.004
Visual Central: Extra Striate Cortex 2 R	35.2	18.6	43.3	19.3	-0.43	-2.0	0.026	0.071	19.9	9.8	22.7	9.0	-0.30	-1.4	0.086	0.149
Visual Central: Extra Striate Cortex 3 R	23.4	14.9	35.8	14.3	-0.85	-3.9	0.000	0.002	24.3	8.0	30.4	7.7	-0.77	-3.6	0.000	0.004
Visual Peripheral: Striate Cortex Calcarine 1 R	25.7	17.7	27.2	15.4	-0.09	-0.4	0.334	0.403	21.2	8.8	28.5	8.5	-0.85	-3.9	0.000	0.002
Visual Peripheral: Extra Striate Inferior 1 R	16.6	10.8	20.6	12.1	-0.35	-1.6	0.055	0.122	33.3	13.0	38.3	10.4	-0.42	-2.0	0.027	0.070
Visual Peripheral: Extra Striate Superior 1 R	21.4	15.7	23.5	12.9	-0.15	-0.7	0.240	0.320	26.5	8.3	34.0	11.3	-0.75	-3.5	0.000	0.004
Somatomotor A: 1 R	12.2	9.2	19.2	11.6	-0.67	-3.1	0.001	0.010	33.8	11.5	38.3	7.8	-0.46	-2.1	0.018	0.053
Somatomotor A: 2 R	16.4	14.7	23.5	14.2	-0.49	-2.3	0.012	0.043	32.2	12.2	33.1	9.0	-0.09	-0.4	0.338	0.388
Somatomotor A: 3 R	15.3	11.6	17.8	11.0	-0.22	-1.0	0.160	0.252	36.3	10.9	38.4	8.1	-0.23	-1.1	0.147	0.216
Somatomotor A: 4 R	25.2	17.9	26.4	16.2	-0.07	-0.3	0.369	0.419	34.5	9.7	37.3	8.6	-0.30	-1.4	0.081	0.148
Somatomotor B: Auditory 1 R	41.0	15.6	42.3	11.8	-0.10	-0.5	0.324	0.400	37.8	12.2	34.8	12.1	0.25	1.2	0.125	0.196
Somatomotor B: S2 1 R	10.5	8.5	17.0	10.5	-0.68	-3.1	0.001	0.010	34.9	10.9	34.0	11.6	0.08	0.4	0.360	0.400
Somatomotor B: S2 2 R	18.9	11.1	24.7	11.5	-0.52	-2.4	0.009	0.038	34.8	9.8	34.3	9.7	0.05	0.2	0.417	0.421
Somatomotor B: Central 1 R	19.8	13.8	23.2	12.1	-0.26	-1.2	0.114	0.197	24.5	14.5	25.5	11.9	-0.08	-0.4	0.354	0.397
Dorsal Attention A: Temporal Occipital 1 R	20.9	13.7	26.9	14.4	-0.43	-2.0	0.025	0.071	37.3	9.4	34.8	10.1	0.25	1.2	0.123	0.196
Dorsal Attention A: Parietal Occipital 1 R	26.3	14.1	32.0	12.7	-0.43	-2.0	0.025	0.071	39.2	10.8	34.9	11.6	0.39	1.8	0.039	0.082
Dorsal Attention A: Superior Parietal Lobule 1 R	27.5	15.3	32.4	13.4	-0.34	-1.6	0.059	0.125	30.3	9.4	34.5	8.4	-0.47	-2.2	0.016	0.049
Dorsal Attention B: Post Central 1 R	15.7	12.5	18.1	11.9	-0.20	-0.9	0.177	0.268	37.1	9.9	35.6	10.0	0.16	0.7	0.235	0.287
Dorsal Attention B: Post Central 2 R	23.6	14.1	25.2	13.3	-0.12	-0.5	0.298	0.375	36.1	9.5	43.5	9.3	-0.78	-3.6	0.000	0.003
Dorsal Attention B: Frontal Eye Fields 1 R	32.3	18.5	27.2	15.2	0.31	1.4	0.081	0.147	37.7	8.6	38.3	10.3	-0.06	-0.3	0.384	0.405
Salience Ventral Attention A: Parietal Operculum 1 R	25.4	13.8	24.5	13.1	0.07	0.3	0.373	0.419	38.0	9.7	36.4	7.9	0.18	0.8	0.201	0.261
Salience Ventral Attention A: Insula: 1 R	27.9	14.5	26.5	11.0	0.12	0.5	0.298	0.375	36.5	9.5	30.3	9.2	0.66	3.1	0.001	0.009
Salience Ventral Attention A: Parietal Medial 1 R	25.8	12.7	24.8	11.2	0.08	0.4	0.357	0.416	35.2	13.0	42.1	10.6	-0.59	-2.7	0.004	0.017
Salience Ventral Attention A: Frontal Medial 1 R	27.5	16.3	24.9	14.0	0.17	0.8	0.220	0.306	35.4	9.9	36.0	9.9	-0.07	-0.3	0.382	0.405
Salience Ventral Attention B: Inferior Parietal Lobule 1 R	22.5	15.4	21.4	11.4	0.08	0.4	0.358	0.416	28.5	13.2	23.8	9.9	0.41	1.9	0.031	0.071
Salience Ventral Attention B: Lateral Prefrontal Cortex 1 R	53.4	11.7	47.1	12.8	0.51	2.4	0.010	0.038	24.3	13.1	21.8	11.0	0.20	0.9	0.174	0.242
Salience Ventral Attention B: Medial Posterior Prefrontal 1 R	28.0	15.3	23.5	14.3	0.30	1.4	0.082	0.147	23.0	10.0	29.2	10.2	-0.61	-2.8	0.003	0.014
Limbic B: Orbital Frontal Cortex 0 R	43.9	20.1	43.5	12.5	0.03	0.1	0.451	0.479	20.8	12.9	15.8	12.1	0.40	1.9	0.032	0.072
Limbic A: Temporal Pole 1 R	27.2	16.1	22.4	13.5	0.32	1.5	0.069	0.130	26.1	10.8	19.1	11.2	0.63	2.9	0.002	0.011
Control A: Intraparietal Sulcus 1 R	25.8	15.9	25.7	10.1	0.00	0.0	0.492	0.492	30.1	9.3	29.5	10.1	0.07	0.3	0.380	0.405
Control A: Lateral Prefrontal Cortex 1 R	53.1	9.7	47.0	12.8	0.53	2.5	0.008	0.034	27.7	12.9	22.6	8.0	0.48	2.2	0.014	0.047
Control A: Lateral Prefrontal Cortex 2 R	48.5	12.6	39.0	13.0	0.75	3.4	0.000	0.005	38.2	11.9	30.9	9.5	0.68	3.1	0.001	0.007
Control B: Temporal 1 R	41.3	16.5	37.7	14.2	0.23	1.1	0.140	0.226	36.4	12.2	24.7	10.6	1.02	4.7	0.000	0.000
Control B: inferior parietal lobule 1 R	39.7	15.2	38.2	15.8	0.09	0.4	0.335	0.403	26.6	11.5	21.9	9.6	0.44	2.1	0.022	0.062
Control B: Lateral Prefrontal Cortexd 1 R	53.7	13.1	46.0	14.2	0.57	2.6	0.005	0.026	29.0	13.0	26.9	9.9	0.19	0.9	0.195	0.261
Control B: Lateral Prefrontal Cortexv 1 R	63.9	8.2	55.6	13.0	0.74	3.4	0.000	0.005	28.4	14.3	22.5	8.8	0.50	2.3	0.011	0.040
Control C: Cingulate Posterior 1 R	30.7	14.1	34.2	12.0	-0.27	-1.2	0.110	0.193	23.5	11.0	22.8	9.6	0.07	0.3	0.370	0.402
Control C: Precuneus 1 R	39.8	12.0	44.0	13.0	-0.33	-1.5	0.063	0.126	26.9	9.4	33.5	9.5	-0.70	-3.2	0.001	0.006
Default A: Inferior Parietal Lobule 1 R	23.8	14.8	27.3	12.8	-0.25	-1.2	0.121	0.205	29.3	12.9	25.5	10.3	0.32	1.5	0.070	0.129
Default A: Dorsal Prefrontal Cortex 1 R	46.1	13.6	36.8	16.5	0.61	2.8	0.003	0.017	26.7	12.7	25.0	9.2	0.16	0.7	0.234	0.287
Default A: Precuneus Posterior Cingulate Cortex 1 R	31.9	12.7	38.1	13.0	-0.48	-2.2	0.014	0.046	26.4	10.2	24.7	9.3	0.18	0.8	0.200	0.261
Default A: Medial Prefrontal Cortex 1 R	42.5	13.5	33.8	12.3	0.68	3.1	0.001	0.010	26.9	11.0	27.9	9.7	-0.10	-0.5	0.325	0.378
Default B: Dorsal Prefrontal Cortex 1 R	54.2	8.5	43.0	15.5	0.88	4.0	0.000	0.002	32.7	12.8	30.3	11.7	0.19	0.9	0.190	0.260
Default B: Ventral Prefrontal Cortex 1 R	32.3	13.8	32.5	10.8	-0.01	-0.1	0.477	0.486	28.7	10.0	25.1	10.5	0.35	1.6	0.055	0.112
Default B: Ventral Prefrontal Cortex 2 R	47.0	11.7	42.8	11.9	0.36	1.6	0.052	0.120	29.6	12.6	25.3	8.1	0.41	1.9	0.031	0.071
Default C: RetroSuperior Parietal Lobuleenial 1 R	16.9	11.1	22.1	10.7	-0.49	-2.3	0.013	0.045	24.3	9.1	26.8	9.2	-0.28	-1.3	0.103	0.171
Default C: Parahippocampal Cortex 1 R	12.2	11.1	14.3	8.9	-0.22	-1.0	0.161	0.252	33.2	12.1	28.5	10.1	0.42	2.0	0.027	0.070
Temporal Parietal 1 R	16.7	12.7	21.9	13.0	-0.41	-1.9	0.031	0.083	35.2	11.1	27.9	13.2	0.59	2.7	0.004	0.016
Temporal Parietal 2 R	28.7	16.4	28.3	12.7	0.03	0.1	0.452	0.479	34.8	11.5	30.2	10.3	0.42	1.9	0.028	0.071
Temporal Parietal 3 R	17.8	12.7	25.5	11.6	-0.63	-2.9	0.002	0.014	35.9	11.6	33.1	11.7	0.24	1.1	0.137	0.206

Supplementary Table 6. Regression analyses predicting cognitive performance from fPET and fMRI whole brain graph metrics and age group in the whole sample (N = 86). The ANOVA results for the overall models are shown in the top three rows, and the standardised beta weights for age and each graph metric's relationship with cognition in the subsequent rows. Significant beta weights are indicated at *p < 0.05 and **p < 0.001.

	HVLT: Delayed Recall	HVLT: Discrim. Index	Digit Span: Forward	Digit Span: Back	Category Switch: % Trials	Category Switch: RT	Dig Sub: Number Correct	Dig Sub: Sec Per Correct	Stop Signal: Pro Reac	Stop Signal: RT
ANOVA F	2.3	3.5	0.2	2.0	1.9	5.5	18.3	0.7	2.5	1.7
ANOVA p	0.028	0.004	0.743	0.077	0.043	0.001	0.001	0.865	0.134	0.193
Variance Explained	17%	24%	1%	16%	15%	33%	63%	6%	18%	13%
fPET: Global Efficiency	0.04	0.21	0.05	-0.07	-0.17	0.14	0.02	-0.17	-0.06	0.00
fPET: Local Efficiency	0.25	0.38*	0.07	-0.07	-0.01	0.21	0.19	-0.06	0.04	-0.02
fPET: Betweenness Centrality	0.08	0.12	0.10	0.30*	0.10	0.01	0.04	0.06	-0.17	-0.09
fMRI: Global Efficiency	0.11	-0.23	-0.06	0.46	0.27	0.09	0.05	-0.03	-0.94	-0.09
fMRI: Local Efficiency	-0.03	-0.05	0.03	-0.06	-0.09	-0.02	-0.11	0.10	-0.11	0.05
fMRI: Betweenness Centrality	0.14	-0.01	-0.07	0.39	0.57	-0.04	0.25	0.05	-0.94*	-0.34
Age Group	-0.28*	-0.30*	0.09	-0.12	-0.18	-0.49**	-0.69**	-0.15	-0.19	-0.29

Discrim = discrimination; Dig Sub = Digit Substitution; Pro React = probability of reacting.

3. Anatomical Parcellation: Harvard Oxford Atlas

There is currently no consensus on the optimal atlas to parcellate metabolic data. In fMRI, it is widely accepted that a 'functional' atlas derived from resting-state fMRI data provides a robust estimates and high interpretability of functional connectivity results (e.g., Schaefer atlas [4]). Brain parcellations derived from a single modality (e.g., resting-state fMRI) show varying levels of transferability to other modalities [5] and it is currently untested how transferable fMRI-derived atlases are to FDG-PET data. Previous results from metabolic covariance analyses [6,7] indicate that fMRI-derived atlases are may not be directly transferable to FDG-PET data. Therefore, we chose to use a fMRI-derived functional atlas for results in the main paper to compare the fPET results to fMRI. However, results for the Harvard Oxford atlas are provided here for comparison. In addition, because PET SNR scales directly with spatial scale and the size of the ROI [8], we chose parcellations with relatively coarse granularity.

We found some differences between the parcellations, such as slightly lower maximum connectivity strength in the functional parcellation but slightly higher topological similarities to fMRI in the anatomical parcellation. The anatomical parcellation appears to be particularly effective at highlighting a reduction in connectivity strength in the frontal regions of the brain in ageing that parallel similar BOLD changes and changes to underlying cerebral metabolic rates of glucose metabolism in ageing [9]. The anatomical parcellation also predicted performance in different aspects of cognition, such as the percentage of correct response inhibition trials and reaction time in the stop signal task. On the basis of these and our previous results [10] at this stage we conclude that fMRI-derived parcellations of fPET data appear to be a valid approach for studying the coherence of dynamic glucose metabolism signals. We encourage the comparison of different parcellation approaches in fPET studies of other populations to further characterise similarities and differences.

Supplementary Table 7. Mean, standard deviation and effect sizes (Cohen's D) of regional global efficiency for younger (N = 40) and older (N = 46) adults in fPET and fMRI from the Harvard Oxford Atlas, and t-tests of age group differences.

	fPET								fMRI							
	Younger		Older		Younger vs Older				Younger		Older		Younger vs Older			
	Mean	SD	Mean	SD	Cohen's D	*t*-value	p	p-FDR	Mean	SD	Mean	SD	Cohen's D	*t*-value	p	p-FDR
Frontal Orbital Cortex L	0.71	0.06	0.67	0.06	0.55	2.6	0.006	0.038	0.68	0.07	0.64	0.09	0.48	2.2	0.015	0.054
Frontal Pole L	0.82	0.04	0.79	0.05	0.72	3.3	0.001	0.010	0.65	0.08	0.64	0.08	0.42	0.7	0.232	0.345
Frontal Operculum Cortex L	0.62	0.06	0.63	0.06	-0.12	-0.5	0.295	0.406	0.63	0.07	0.62	0.07	0.16	0.6	0.288	0.368
Superior Frontal Gyrus L	0.73	0.08	0.68	0.08	0.66	3.1	0.002	0.018	0.63	0.08	0.64	0.06	0.12	-0.3	0.381	0.444
Middle Frontal Gyrus L	0.76	0.04	0.72	0.06	0.72	3.3	0.001	0.010	0.61	0.07	0.62	0.07	0.12	-0.7	0.241	0.345
Inferior Frontal Gyrus; Pars Triangularis L	0.70	0.06	0.68	0.05	0.34	1.6	0.058	0.143	0.62	0.07	0.61	0.06	-0.07	0.7	0.237	0.345
Inferior Frontal Gyrus; pars opercularis L	0.67	0.07	0.64	0.06	0.46	2.1	0.019	0.074	0.63	0.06	0.62	0.07	-0.15	0.7	0.257	0.349
Paracingulate Gyrus L	0.71	0.05	0.65	0.06	1.02	4.7	0.000	0.000	0.61	0.08	0.63	0.07	-0.15	-1.0	0.169	0.280
Insular Cortex L	0.57	0.11	0.58	0.10	-0.09	-0.4	0.342	0.409	0.66	0.07	0.66	0.05	0.15	-0.4	0.334	0.408
Amygdala l	0.54	0.09	0.56	0.06	-0.31	-1.4	0.076	0.168	0.60	0.08	0.52	0.17	0.14	2.8	0.003	0.017
Juxtapositional Lobule Cortex - L	0.62	0.08	0.61	0.12	0.08	0.4	0.350	0.409	0.63	0.07	0.66	0.05	0.14	-2.6	0.005	0.028
Precentral Gyrus L	0.72	0.08	0.69	0.07	0.34	1.6	0.061	0.147	0.67	0.09	0.71	0.05	-0.21	-2.4	0.009	0.040
PostCG l Postcentral Gyrus L	0.70	0.08	0.70	0.08	-0.05	-0.2	0.418	0.443	0.66	0.08	0.70	0.04	-0.09	-3.4	0.001	0.006
Central Opercular Cortex L	0.59	0.07	0.63	0.07	-0.55	-2.6	0.006	0.038	0.67	0.07	0.67	0.06	-0.11	0.1	0.468	0.491
Superior Parietal Lobule L	0.67	0.09	0.66	0.06	0.21	1.0	0.164	0.285	0.64	0.07	0.69	0.05	0.61	-3.9	0.000	0.003
Supramarginal Gyrus; Anterior Division L	0.66	0.07	0.63	0.06	0.53	2.4	0.008	0.044	0.63	0.06	0.64	0.05	-0.57	-0.9	0.173	0.282
Supramarginal Gyrus; Posterior Division L	0.69	0.06	0.67	0.05	0.33	1.5	0.064	0.148	0.65	0.07	0.66	0.07	-0.69	-0.4	0.327	0.408
Angular Gyrus L	0.65	0.07	0.66	0.05	-0.06	-0.3	0.400	0.435	0.66	0.07	0.61	0.07	-0.51	3.5	0.000	0.004
Parietal Operculum Cortex L	0.62	0.08	0.63	0.06	-0.11	-0.5	0.308	0.408	0.68	0.06	0.68	0.07	-0.74	0.0	0.500	0.500
Parahippocampal Gyrus; Anterior Division L	0.54	0.08	0.57	0.10	-0.31	-1.4	0.080	0.172	0.61	0.09	0.60	0.11	-1.11	0.4	0.343	0.409
Parahippocampal Gyrus; Posterior Division L	0.52	0.06	0.56	0.11	-0.37	-1.7	0.045	0.118	0.59	0.09	0.59	0.09	0.02	0.0	0.488	0.498
Hippocampus l	0.56	0.09	0.56	0.11	-0.04	-0.2	0.422	0.443	0.64	0.07	0.59	0.12	-0.84	2.2	0.015	0.054
Thalamus L	0.55	0.06	0.59	0.11	-0.52	-2.4	0.009	0.046	0.60	0.10	0.59	0.10	-1.10	0.6	0.269	0.356
Caudate L	0.58	0.07	0.63	0.11	-0.55	-2.5	0.007	0.038	0.56	0.13	0.57	0.09	-0.20	-0.4	0.342	0.409
Putamen L	0.63	0.08	0.65	0.12	-0.19	-0.9	0.190	0.315	0.57	0.13	0.57	0.09	-0.10	0.0	0.493	0.498
Pallidum L	0.53	0.07	0.58	0.10	-0.62	-2.8	0.003	0.027	0.56	0.16	0.53	0.17	-0.10	0.6	0.276	0.356
Accumbens L	0.53	0.07	0.58	0.10	-0.57	-2.7	0.005	0.036	0.54	0.14	0.47	0.21	0.76	1.8	0.040	0.112
Temporal Pole L	0.60	0.07	0.59	0.11	0.19	0.9	0.193	0.315	0.69	0.05	0.67	0.06	0.00	1.5	0.068	0.155
Planum Polare L	0.53	0.07	0.57	0.10	-0.41	-1.9	0.029	0.092	0.64	0.07	0.62	0.09	0.00	1.1	0.130	0.242
Superior Temporal Gyrus; Anterior Division L	0.55	0.07	0.58	0.06	-0.47	-2.2	0.017	0.072	0.65	0.07	0.65	0.09	0.09	0.2	0.418	0.466
Superior Temporal Gyrus; Posterior Division L	0.60	0.07	0.63	0.05	-0.50	-2.3	0.011	0.055	0.67	0.07	0.67	0.07	-0.01	-0.1	0.447	0.483
Middle Temporal Gyrus; Anterior Division L	0.59	0.07	0.59	0.06	0.06	0.3	0.398	0.435	0.63	0.09	0.63	0.07	-0.01	0.0	0.490	0.498
Middle Temporal Gyrus; Posterior Division L	0.72	0.07	0.67	0.07	0.76	3.5	0.000	0.009	0.66	0.08	0.64	0.06	0.47	1.3	0.094	0.185
Inferior Temporal Gyrus; Anterior Division L	0.58	0.07	0.57	0.10	0.13	0.6	0.279	0.395	0.63	0.06	0.59	0.08	0.13	2.2	0.015	0.054
Inferior Temporal Gyrus; Posterior Division L	0.67	0.06	0.62	0.07	0.65	3.0	0.002	0.018	0.67	0.07	0.60	0.07	0.13	4.0	0.000	0.003
Planum Temporale L	0.70	0.06	0.68	0.05	0.44	2.0	0.022	0.080	0.68	0.07	0.70	0.07	-0.09	-1.4	0.079	0.170
Heschl's Gyrus L	0.64	0.07	0.66	0.06	-0.28	-1.3	0.101	0.194	0.64	0.07	0.65	0.08	0.00	-0.8	0.220	0.338
Temporal Fusiform Cortex; Anterior Division L	0.54	0.06	0.57	0.11	-0.28	-1.3	0.101	0.194	0.57	0.13	0.58	0.12	0.00	-0.3	0.379	0.444
Temporal Fusiform Cortex; Posterior Division L	0.58	0.06	0.59	0.10	-0.13	-0.6	0.280	0.395	0.67	0.08	0.66	0.05	0.13	0.8	0.214	0.334
Temporal Occipital Fusiform Cortex L	0.58	0.06	0.61	0.06	-0.60	-2.8	0.003	0.031	0.66	0.06	0.65	0.06	0.38	0.7	0.250	0.349
Middle Temporal Gyrus; Temporooccipital Part L	0.63	0.08	0.64	0.06	-0.07	-0.3	0.372	0.424	0.67	0.07	0.64	0.05	0.33	2.0	0.023	0.073
Inferior Temporal Gyrus; Temporooccipital Part L	0.60	0.07	0.61	0.05	-0.20	-0.9	0.175	0.298	0.66	0.06	0.63	0.06	0.32	2.4	0.009	0.040
Occipital Fusiform Gyrus L	0.64	0.09	0.66	0.07	-0.30	-1.4	0.081	0.172	0.62	0.07	0.65	0.06	0.25	-1.8	0.039	0.111
Supracalcarine Cortex L	0.61	0.05	0.63	0.06	-0.25	-1.2	0.122	0.227	0.59	0.08	0.64	0.05	0.22	-4.0	0.000	0.003
Cuneal Cortex L	0.61	0.07	0.62	0.06	-0.16	-0.7	0.233	0.363	0.61	0.08	0.67	0.06	0.04	-3.8	0.000	0.003
Lingual Gyrus L	0.61	0.12	0.63	0.12	-0.11	-0.5	0.300	0.406	0.61	0.07	0.65	0.05	-0.03	-2.6	0.005	0.028
Intracalcarine Cortex L	0.64	0.08	0.64	0.07	0.02	0.1	0.458	0.467	0.58	0.08	0.64	0.06	-0.03	-3.7	0.000	0.003
Lateral Occipital Cortex; Superior Division L	0.77	0.05	0.75	0.06	0.39	1.8	0.039	0.112	0.67	0.06	0.68	0.05	0.01	-0.7	0.254	0.349
Lateral Occipital Cortex; Inferior Division L	0.65	0.09	0.68	0.07	-0.34	-1.6	0.062	0.147	0.67	0.06	0.67	0.06	0.29	0.0	0.485	0.498
Occipital Pole L	0.69	0.08	0.70	0.08	-0.15	-0.7	0.238	0.366	0.59	0.07	0.63	0.06	0.34	-3.3	0.001	0.007
Frontal Medial Cortex	0.71	0.08	0.68	0.06	0.35	1.6	0.056	0.140	0.62	0.08	0.62	0.08	0.47	0.5	0.302	0.381
Cingulate Gyrus; Anterior Division	0.70	0.08	0.65	0.13	0.46	2.1	0.018	0.074	0.62	0.08	0.65	0.06	0.86	-2.5	0.007	0.036
Subcallosal Cortex	0.62	0.09	0.60	0.11	0.16	0.8	0.226	0.358	0.60	0.12	0.57	0.16	0.83	0.9	0.189	0.303
Cingulate Gyrus; Posterior Division	0.76	0.07	0.76	0.07	0.00	0.0	0.496	0.496	0.60	0.09	0.63	0.06	-0.30	-1.6	0.062	0.145
Precuneous Cortex	0.80	0.06	0.80	0.05	-0.09	-0.4	0.341	0.409	0.60	0.08	0.66	0.06	-0.17	-3.6	0.000	0.004
Brain-Stem	0.69	0.09	0.64	0.08	0.56	2.6	0.005	0.038	0.49	0.21	0.55	0.13	-0.16	-1.7	0.051	0.133
Frontal Orbital Cortex R	0.72	0.07	0.69	0.06	0.37	1.7	0.044	0.118	0.66	0.06	0.64	0.07	-0.07	1.6	0.053	0.133
Frontal Pole R	0.84	0.03	0.81	0.05	0.77	3.6	0.000	0.009	0.66	0.08	0.63	0.07	0.17	1.7	0.044	0.120
Frontal Operculum Cortex R	0.64	0.07	0.66	0.06	-0.29	-1.4	0.089	0.183	0.64	0.07	0.62	0.07	0.22	1.4	0.080	0.170
Superior Frontal Gyrus R	0.72	0.08	0.69	0.07	0.48	2.2	0.015	0.064	0.65	0.07	0.65	0.06	0.14	-0.2	0.427	0.470
Middle Frontal Gyrus R	0.78	0.07	0.75	0.07	0.55	2.5	0.007	0.038	0.62	0.07	0.61	0.06	0.44	0.2	0.430	0.470
Inferior Frontal Gyrus; Pars Triangularis R	0.70	0.05	0.69	0.05	0.13	0.6	0.277	0.395	0.65	0.07	0.63	0.05	0.51	1.4	0.077	0.170
Inferior Frontal Gyrus; Pars Opercularis R	0.73	0.05	0.69	0.05	0.88	4.1	0.000	0.003	0.65	0.07	0.61	0.06	0.52	2.3	0.013	0.051
Paracingulate Gyrus R	0.72	0.06	0.68	0.06	0.75	3.5	0.000	0.009	0.60	0.08	0.63	0.06	-0.39	-2.0	0.027	0.084
Insular Cortex R	0.60	0.07	0.60	0.10	0.10	0.4	0.329	0.409	0.68	0.05	0.68	0.05	-0.44	-0.1	0.454	0.483
Amygdala r	0.56	0.12	0.58	0.07	-0.23	-1.1	0.144	0.261	0.59	0.08	0.55	0.13	-0.85	1.7	0.047	0.125
Juxtapositional Lobule Cortex R	0.60	0.13	0.60	0.11	-0.05	-0.2	0.408	0.437	0.63	0.07	0.67	0.05	-0.82	-3.0	0.002	0.014
Precentral Gyrus R	0.73	0.06	0.70	0.08	0.41	1.9	0.031	0.093	0.69	0.07	0.70	0.04	-0.96	-1.0	0.158	0.269
Postcentral Gyrus R	0.66	0.07	0.66	0.08	-0.05	-0.2	0.402	0.435	0.68	0.06	0.69	0.05	-0.56	-1.1	0.135	0.243
Central Opercular Cortex R	0.62	0.10	0.65	0.06	-0.41	-1.9	0.030	0.092	0.67	0.06	0.68	0.06	-0.80	-0.7	0.238	0.345
Superior Parietal Lobule R	0.62	0.13	0.63	0.07	-0.06	-0.3	0.384	0.429	0.65	0.05	0.67	0.06	-1.00	-2.1	0.019	0.064
Supramarginal Gyrus; Anterior Division R	0.61	0.07	0.61	0.05	0.10	0.5	0.323	0.409	0.65	0.06	0.63	0.05	-0.14	1.1	0.148	0.257
Supramarginal Gyrus; Posterior Division R	0.65	0.07	0.66	0.06	-0.12	-0.6	0.287	0.401	0.64	0.07	0.64	0.06	-0.01	0.3	0.395	0.450
Angular Gyrus R	0.67	0.07	0.68	0.07	-0.08	-0.4	0.351	0.409	0.62	0.08	0.61	0.06	-0.01	1.2	0.113	0.217
Parietal Operculum Cortex R	0.60	0.07	0.61	0.08	-0.08	-0.4	0.349	0.409	0.66	0.07	0.68	0.06	-0.70	-1.5	0.069	0.155
Parahippocampal Gyrus; Anterior Division R	0.54	0.08	0.57	0.10	-0.33	-1.5	0.066	0.150	0.62	0.07	0.63	0.08	0.11	-0.4	0.335	0.408
Parahippocampal Gyrus; Posterior Division R	0.49	0.17	0.55	0.13	-0.39	-1.8	0.037	0.110	0.61	0.08	0.58	0.12	0.11	1.0	0.161	0.272
Hippocampus R	0.56	0.09	0.57	0.10	-0.15	-0.7	0.248	0.376	0.62	0.08	0.64	0.08	-0.54	-0.6	0.271	0.356
Thalamus R	0.55	0.11	0.59	0.11	-0.37	-1.7	0.045	0.118	0.62	0.09	0.62	0.08	0.19	0.1	0.456	0.483
Caudate R	0.56	0.07	0.61	0.11	-0.59	-2.7	0.004	0.031	0.56	0.12	0.59	0.10	0.17	-1.2	0.121	0.230
Putamen R	0.61	0.09	0.66	0.12	-0.43	-2.0	0.025	0.087	0.57	0.12	0.59	0.08	-0.33	-0.7	0.236	0.345
Pallidum R	0.52	0.08	0.56	0.11	-0.42	-1.9	0.027	0.091	0.55	0.14	0.53	0.13	-0.77	0.7	0.245	0.346
Accumbens R	0.53	0.07	0.57	0.10	-0.49	-2.3	0.013	0.062	0.52	0.16	0.51	0.17	-0.91	0.3	0.390	0.449
Temporal Pole R	0.62	0.07	0.62	0.12	0.10	0.4	0.329	0.409	0.70	0.05	0.68	0.06	-0.35	2.1	0.019	0.064
Planum Polare R	0.57	0.08	0.59	0.10	-0.13	-0.6	0.274	0.395	0.66	0.06	0.64	0.08	0.35	1.4	0.082	0.170
Superior Temporal Gyrus; Anterior Division R	0.55	0.07	0.59	0.10	-0.44	-2.0	0.022	0.080	0.70	0.06	0.68	0.07	0.33	1.1	0.133	0.243
Superior Temporal Gyrus; Posterior Division R	0.66	0.07	0.65	0.05	0.10	0.4	0.328	0.409	0.69	0.06	0.66	0.06	0.37	2.4	0.009	0.040
Middle Temporal Gyrus; Anterior Division R	0.64	0.06	0.63	0.05	0.23	1.1	0.145	0.261	0.66	0.05	0.63	0.05	0.31	2.9	0.003	0.017
Middle Temporal Gyrus; Posterior Division R	0.72	0.07	0.68	0.07	0.65	3.0	0.002	0.018	0.68	0.06	0.65	0.05	0.29	3.2	0.001	0.008
Inferior Temporal Gyrus; Anterior Division R	0.62	0.07	0.59	0.10	0.29	1.4	0.090	0.183	0.64	0.07	0.61	0.08	-0.04	1.4	0.084	0.172
Inferior Temporal Gyrus; Posterior Division R	0.64	0.06	0.63	0.07	0.22	1.0	0.157	0.278	0.64	0.08	0.62	0.09	0.04	1.1	0.142	0.251
Planum Temporale R	0.67	0.06	0.67	0.05	-0.04	-0.2	0.426	0.443	0.69	0.06	0.71	0.05	0.04	-1.8	0.037	0.110
Heschl's Gyrus R	0.63	0.07	0.65	0.06	-0.35	-1.6	0.053	0.136	0.65	0.07	0.65	0.07	0.31	0.3	0.399	0.450
Temporal Fusiform Cortex; Anterior Division R	0.53	0.08	0.57	0.10	-0.43	-2.0	0.026	0.089	0.58	0.09	0.59	0.08	0.49	-0.9	0.196	0.309
Temporal Fusiform Cortex; Posterior Division R	0.58	0.07	0.59	0.10	-0.10	-0.5	0.319	0.409	0.68	0.05	0.65	0.06	0.56	2.3	0.012	0.048
Temporal Occipital Fusiform Cortex R	0.59	0.06	0.60	0.10	-0.14	-0.6	0.259	0.387	0.65	0.07	0.68	0.07	-0.42	-1.6	0.059	0.142
Middle Temporal Gyrus; Temporooccipital Part R	0.65	0.06	0.65	0.05	-0.03	-0.1	0.442	0.455	0.69	0.07	0.66	0.07	-0.03	1.6	0.055	0.136
Inferior Temporal Gyrus; Temporooccipital Part R	0.60	0.06	0.61	0.06	-0.11	-0.5	0.303	0.406	0.66	0.06	0.63	0.06	-0.02	2.7	0.004	0.026
Occipital Fusiform Gyrus R	0.64	0.08	0.65	0.06	-0.17	-0.8	0.220	0.353	0.60	0.06	0.65	0.06	0.36	-3.3	0.001	0.007
Supracalcarine Cortex R	0.65	0.07	0.63	0.05	0.28	1.3	0.100	0.194	0.57	0.07	0.63	0.07	-0.64	-4.4	0.000	0.002
Cuneal Cortex R	0.63	0.06	0.64	0.06	-0.09	-0.4	0.336	0.409	0.61	0.09	0.66	0.06	-0.85	-3.4	0.001	0.006
Lingual Gyrus R	0.60	0.12	0.63	0.11	-0.20	-0.9	0.177	0.298	0.61	0.06	0.65	0.06	-0.22	-3.0	0.002	0.012
Intracalcarine Cortex R	0.64	0.07	0.64	0.05	0.07	0.3	0.376	0.424	0.59	0.08	0.63	0.06	-0.24	-2.4	0.008	0.040
Lateral Occipital Cortex; Superior Division R	0.75	0.06	0.76	0.06	-0.08	-0.4	0.360	0.415	0.65	0.05	0.67	0.05	-0.26	-1.4	0.090	0.180
Lateral Occipital Cortex; Inferior Division R	0.67	0.08	0.69	0.07	-0.28	-1.3	0.102	0.194	0.66	0.05	0.67	0.05	-0.15	-0.6	0.273	0.356
Occipital Pole R	0.71	0.09	0.71	0.07	0.02	0.1	0.462	0.467	0.59	0.08	0.62	0.06	-0.45	-1.8	0.035	0.106

Supplementary Table 8. Mean, standard deviation and effect sizes (Cohen's D) of regional local efficiency for younger (N = 40) and older (N = 46) adults in fPET and fMRI from the Harvard Oxford Atlas, and t-tests of age group differences.

	fPET								fMRI							
	Younger		Older		Younger vs Older				Younger		Older		Younger vs Older			
	Mean	SD	Mean	SD	Cohen's D	*t*-value	p	p-FDR	Mean	SD	Mean	SD	Cohen's D	*t*-value	p	p-FDR
Frontal Orbital Cortex L	0.78	0.06	0.75	0.07	0.42	1.9	0.027	0.061	0.75	0.08	0.75	0.05	-0.44	0.3	0.391	0.491
Frontal Pole L	0.75	0.03	0.74	0.04	0.41	1.9	0.031	0.067	0.78	0.06	0.74	0.07	0.23	2.7	0.004	0.081
Frontal Operculum Cortex L	0.73	0.14	0.72	0.11	0.13	0.6	0.273	0.340	0.78	0.06	0.76	0.06	0.06	1.3	0.096	0.356
Superior Frontal Gyrus L	0.78	0.03	0.74	0.10	0.58	2.7	0.004	0.019	0.75	0.09	0.76	0.05	0.07	-0.1	0.471	0.495
Middle Frontal Gyrus L	0.78	0.03	0.75	0.05	0.82	3.8	0.000	0.001	0.77	0.07	0.76	0.05	0.26	0.7	0.245	0.433
Inferior Frontal Gyrus; Pars Triangularis L	0.80	0.07	0.75	0.08	0.72	3.3	0.001	0.005	0.77	0.08	0.77	0.07	-0.32	0.0	0.500	0.500
Inferior Frontal Gyrus; pars opercularis L	0.79	0.08	0.75	0.08	0.55	2.6	0.006	0.020	0.78	0.05	0.75	0.09	-0.34	1.4	0.082	0.347
Paracingulate Gyrus L	0.81	0.04	0.74	0.09	1.01	4.7	0.000	0.000	0.76	0.08	0.73	0.11	-0.09	1.4	0.082	0.347
Insular Cortex L	0.67	0.19	0.65	0.11	0.08	0.4	0.354	0.395	0.77	0.06	0.77	0.05	0.21	-0.4	0.346	0.480
Amygdala l	0.52	0.26	0.53	0.20	-0.08	-0.4	0.359	0.397	0.78	0.11	0.77	0.21	0.19	0.3	0.400	0.491
Juxtapositional Lobule Cortex - L	0.77	0.18	0.73	0.09	0.26	1.2	0.117	0.189	0.79	0.09	0.79	0.06	-0.13	-0.4	0.356	0.480
Precentral Gyrus L	0.78	0.07	0.73	0.08	0.73	3.4	0.001	0.005	0.72	0.17	0.78	0.05	0.02	-2.3	0.013	0.175
PostCG l Postcentral Gyrus L	0.78	0.09	0.72	0.12	0.57	2.6	0.005	0.020	0.75	0.17	0.78	0.04	0.03	-1.3	0.097	0.356
Central Opercular Cortex L	0.67	0.18	0.72	0.09	-0.31	-1.4	0.079	0.141	0.78	0.08	0.79	0.06	-0.25	-1.1	0.132	0.368
Superior Parietal Lobule L	0.79	0.07	0.75	0.09	0.57	2.6	0.005	0.020	0.75	0.11	0.77	0.05	-0.16	-0.9	0.177	0.373
Supramarginal Gyrus; Anterior Division L	0.81	0.08	0.73	0.09	0.97	4.5	0.000	0.000	0.80	0.07	0.80	0.07	-0.19	-0.4	0.353	0.480
Supramarginal Gyrus; Posterior Division L	0.80	0.05	0.74	0.07	0.99	4.6	0.000	0.000	0.76	0.05	0.76	0.05	0.15	-0.2	0.436	0.491
Angular Gyrus L	0.80	0.08	0.74	0.09	0.64	3.0	0.002	0.011	0.76	0.05	0.76	0.06	0.06	-0.2	0.418	0.491
Parietal Operculum Cortex L	0.73	0.18	0.72	0.11	0.03	0.1	0.452	0.480	0.80	0.04	0.80	0.05	0.06	0.1	0.452	0.491
Parahippocampal Gyrus; Anterior Division L	0.51	0.25	0.57	0.17	-0.26	-1.2	0.116	0.188	0.79	0.08	0.79	0.08	0.45	0.2	0.439	0.491
Parahippocampal Gyrus; Posterior Division L	0.53	0.28	0.57	0.19	-0.16	-0.7	0.229	0.307	0.78	0.16	0.79	0.15	0.30	-0.4	0.358	0.480
Hippocampus l	0.55	0.28	0.55	0.19	-0.01	0.0	0.488	0.494	0.76	0.08	0.76	0.13	0.27	-0.2	0.406	0.491
Thalamus L	0.60	0.29	0.70	0.15	-0.43	-2.0	0.025	0.058	0.74	0.16	0.75	0.14	0.24	-0.2	0.413	0.491
Caudate L	0.64	0.22	0.71	0.07	-0.42	-2.0	0.027	0.061	0.75	0.12	0.80	0.09	0.52	-1.9	0.030	0.250
Putamen L	0.70	0.18	0.72	0.11	-0.17	-0.8	0.212	0.292	0.79	0.11	0.77	0.14	0.51	0.7	0.228	0.417
Pallidum L	0.56	0.25	0.62	0.15	-0.33	-1.5	0.068	0.126	0.83	0.10	0.75	0.20	0.62	2.3	0.012	0.175
Accumbens L	0.57	0.27	0.68	0.15	-0.51	-2.3	0.012	0.033	0.78	0.14	0.82	0.13	0.69	-1.1	0.144	0.368
Temporal Pole L	0.66	0.17	0.60	0.20	0.32	1.5	0.073	0.134	0.79	0.06	0.75	0.05	0.72	3.3	0.001	0.043
Planum Polare L	0.56	0.29	0.65	0.15	-0.40	-1.8	0.034	0.072	0.79	0.05	0.76	0.18	0.30	1.0	0.158	0.368
Superior Temporal Gyrus; Anterior Division L	0.56	0.27	0.64	0.14	-0.37	-1.7	0.046	0.094	0.78	0.05	0.80	0.08	0.23	-1.0	0.171	0.373
Superior Temporal Gyrus; Posterior Division L	0.73	0.17	0.73	0.09	0.02	0.1	0.465	0.488	0.77	0.05	0.77	0.05	0.22	0.2	0.433	0.491
Middle Temporal Gyrus; Anterior Division L	0.61	0.25	0.65	0.11	-0.21	-1.0	0.164	0.241	0.74	0.19	0.78	0.07	-0.39	-1.3	0.104	0.357
Middle Temporal Gyrus; Posterior Division L	0.76	0.07	0.71	0.09	0.57	2.6	0.005	0.020	0.78	0.05	0.76	0.07	0.06	1.5	0.064	0.322
Inferior Temporal Gyrus; Anterior Division L	0.67	0.21	0.58	0.20	0.43	2.0	0.024	0.058	0.81	0.06	0.79	0.07	0.06	1.6	0.055	0.292
Inferior Temporal Gyrus; Posterior Division L	0.76	0.09	0.68	0.17	0.63	2.9	0.002	0.011	0.78	0.06	0.76	0.09	-0.18	0.9	0.176	0.373
Planum Temporale L	0.79	0.06	0.75	0.09	0.52	2.4	0.010	0.029	0.77	0.06	0.78	0.05	0.50	-0.6	0.275	0.463
Heschl's Gyrus L	0.80	0.09	0.74	0.09	0.66	3.1	0.001	0.009	0.78	0.07	0.76	0.09	0.47	1.2	0.125	0.368
Temporal Fusiform Cortex; Anterior Division L	0.56	0.25	0.59	0.16	-0.12	-0.5	0.295	0.347	0.78	0.18	0.79	0.14	-0.34	-0.2	0.417	0.491
Temporal Fusiform Cortex; Posterior Division L	0.69	0.14	0.64	0.13	0.40	1.8	0.035	0.073	0.78	0.06	0.77	0.06	0.35	0.8	0.212	0.400
Temporal Occipital Fusiform Cortex L	0.68	0.19	0.70	0.11	-0.14	-0.6	0.264	0.340	0.79	0.06	0.80	0.06	0.35	-1.0	0.161	0.368
Middle Temporal Gyrus; Temporooccipital Part L	0.75	0.12	0.74	0.09	0.12	0.5	0.296	0.347	0.78	0.05	0.77	0.09	0.57	0.6	0.287	0.465
Inferior Temporal Gyrus; Temporooccipital Part L	0.69	0.19	0.70	0.10	-0.10	-0.5	0.319	0.367	0.77	0.06	0.78	0.05	-0.71	-0.4	0.328	0.480
Occipital Fusiform Gyrus L	0.74	0.12	0.72	0.12	0.13	0.6	0.269	0.340	0.82	0.07	0.83	0.06	-0.73	-0.3	0.386	0.491
Supracalcarine Cortex L	0.77	0.11	0.71	0.12	0.56	2.6	0.005	0.020	0.83	0.07	0.84	0.05	-0.93	-1.0	0.153	0.368
Cuneal Cortex L	0.74	0.15	0.71	0.13	0.21	1.0	0.171	0.248	0.82	0.07	0.82	0.05	-0.73	-0.4	0.352	0.480
Lingual Gyrus L	0.73	0.12	0.70	0.10	0.25	1.2	0.125	0.194	0.82	0.06	0.84	0.05	-0.90	-1.7	0.042	0.262
Intracalcarine Cortex L	0.74	0.15	0.70	0.13	0.27	1.2	0.108	0.182	0.83	0.08	0.85	0.06	-0.65	-0.8	0.202	0.393
Lateral Occipital Cortex; Superior Division L	0.77	0.03	0.74	0.04	1.00	4.6	0.000	0.000	0.74	0.06	0.76	0.06	-0.53	-1.1	0.137	0.368
Lateral Occipital Cortex; Inferior Division L	0.71	0.12	0.72	0.08	-0.13	-0.6	0.272	0.340	0.78	0.04	0.79	0.04	-0.61	-0.6	0.264	0.451
Occipital Pole L	0.76	0.08	0.72	0.09	0.49	2.2	0.014	0.038	0.83	0.08	0.84	0.07	-0.29	-1.1	0.139	0.368
Frontal Medial Cortex	0.76	0.08	0.74	0.08	0.26	1.2	0.112	0.186	0.77	0.11	0.77	0.07	-0.13	-0.1	0.459	0.491
Cingulate Gyrus; Anterior Division	0.80	0.09	0.72	0.13	0.73	3.3	0.001	0.005	0.76	0.08	0.76	0.05	-0.13	-0.1	0.461	0.491
Subcallosal Cortex	0.70	0.21	0.71	0.12	-0.06	-0.3	0.399	0.436	0.76	0.15	0.75	0.14	-0.39	0.2	0.420	0.491
Cingulate Gyrus; Posterior Division	0.77	0.13	0.75	0.06	0.13	0.6	0.277	0.342	0.70	0.13	0.74	0.10	0.06	-1.6	0.053	0.292
Precuneous Cortex	0.76	0.03	0.73	0.04	0.88	4.1	0.000	0.001	0.70	0.15	0.77	0.06	0.07	-2.8	0.003	0.081
Brain-Stem	0.81	0.07	0.71	0.16	0.73	3.3	0.001	0.005	0.80	0.11	0.77	0.15	0.59	1.3	0.101	0.356
Frontal Orbital Cortex R	0.78	0.07	0.75	0.08	0.48	2.2	0.015	0.041	0.77	0.06	0.75	0.07	0.28	1.9	0.031	0.250
Frontal Pole R	0.74	0.02	0.73	0.03	0.29	1.4	0.089	0.155	0.77	0.06	0.74	0.08	0.28	1.7	0.042	0.262
Frontal Operculum Cortex R	0.78	0.15	0.75	0.09	0.24	1.1	0.140	0.212	0.78	0.06	0.79	0.08	-0.02	-0.2	0.439	0.491
Superior Frontal Gyrus R	0.77	0.13	0.74	0.10	0.34	1.6	0.059	0.111	0.77	0.07	0.74	0.08	0.15	2.2	0.015	0.177
Middle Frontal Gyrus R	0.77	0.05	0.74	0.05	0.70	3.2	0.001	0.006	0.77	0.06	0.76	0.07	0.17	0.7	0.240	0.432
Inferior Frontal Gyrus; Pars Triangularis R	0.82	0.07	0.76	0.06	0.89	4.1	0.000	0.001	0.77	0.06	0.76	0.05	0.00	0.8	0.227	0.417
Inferior Frontal Gyrus; Pars Opercularis R	0.81	0.05	0.76	0.07	0.83	3.8	0.000	0.001	0.76	0.09	0.74	0.11	0.30	1.0	0.149	0.368
Paracingulate Gyrus R	0.80	0.05	0.77	0.06	0.51	2.3	0.011	0.032	0.75	0.11	0.75	0.06	0.26	-0.1	0.463	0.491
Insular Cortex R	0.72	0.17	0.69	0.12	0.20	0.9	0.175	0.250	0.77	0.06	0.76	0.05	0.30	0.9	0.181	0.373
Amygdala r	0.58	0.28	0.58	0.18	0.00	0.0	0.491	0.494	0.79	0.12	0.82	0.11	-0.09	-1.1	0.132	0.368
Juxtapositional Lobule Cortex R	0.77	0.17	0.70	0.11	0.46	2.1	0.020	0.048	0.81	0.06	0.79	0.06	-0.09	1.5	0.069	0.331
Precentral Gyrus R	0.79	0.05	0.73	0.07	0.98	4.5	0.000	0.000	0.80	0.05	0.78	0.05	0.06	1.1	0.135	0.368
Postcentral Gyrus R	0.80	0.10	0.68	0.17	0.80	3.7	0.000	0.002	0.79	0.07	0.78	0.05	-0.08	0.4	0.344	0.480
Central Opercular Cortex R	0.74	0.15	0.72	0.10	0.12	0.5	0.298	0.347	0.80	0.06	0.80	0.05	-0.10	0.5	0.293	0.465
Superior Parietal Lobule R	0.79	0.09	0.73	0.12	0.56	2.6	0.006	0.020	0.78	0.07	0.77	0.07	-0.48	0.6	0.279	0.463
Supramarginal Gyrus; Anterior Division R	0.79	0.09	0.69	0.12	0.98	4.5	0.000	0.000	0.79	0.08	0.80	0.07	-0.28	-0.7	0.252	0.438
Supramarginal Gyrus; Posterior Division R	0.79	0.08	0.73	0.09	0.61	2.8	0.003	0.014	0.77	0.06	0.77	0.06	-0.83	0.0	0.496	0.500
Angular Gyrus R	0.78	0.09	0.73	0.10	0.56	2.6	0.006	0.020	0.77	0.08	0.76	0.07	-0.24	0.8	0.204	0.393
Parietal Operculum Cortex R	0.72	0.18	0.67	0.18	0.26	1.2	0.119	0.189	0.80	0.06	0.81	0.05	-0.20	-0.1	0.447	0.491
Parahippocampal Gyrus; Anterior Division R	0.50	0.23	0.57	0.18	-0.37	-1.7	0.047	0.094	0.80	0.06	0.79	0.07	-0.35	0.5	0.308	0.474
Parahippocampal Gyrus; Posterior Division R	0.57	0.19	0.59	0.15	-0.12	-0.6	0.291	0.347	0.75	0.11	0.82	0.10	-0.08	-3.0	0.002	0.061
Hippocampus R	0.56	0.29	0.57	0.23	-0.03	-0.1	0.442	0.473	0.74	0.14	0.77	0.07	-0.04	-1.2	0.122	0.368
Thalamus R	0.68	0.18	0.65	0.19	0.17	0.8	0.224	0.304	0.74	0.11	0.77	0.07	-0.04	-1.2	0.109	0.361
Caudate R	0.61	0.23	0.68	0.15	-0.35	-1.6	0.055	0.107	0.78	0.10	0.77	0.10	-0.04	0.5	0.294	0.465
Putamen R	0.67	0.16	0.73	0.06	-0.47	-2.2	0.016	0.042	0.78	0.09	0.75	0.15	0.03	1.0	0.163	0.368
Pallidum R	0.50	0.28	0.60	0.17	-0.49	-2.2	0.015	0.041	0.77	0.18	0.77	0.16	0.02	0.0	0.488	0.500
Accumbens R	0.53	0.23	0.63	0.12	-0.54	-2.5	0.008	0.025	0.79	0.14	0.82	0.13	0.03	-0.9	0.187	0.373
Temporal Pole R	0.74	0.12	0.66	0.13	0.64	2.9	0.002	0.011	0.78	0.05	0.74	0.05	-0.08	3.3	0.001	0.043
Planum Polare R	0.65	0.19	0.67	0.16	-0.09	-0.4	0.334	0.377	0.81	0.07	0.80	0.08	-0.08	0.4	0.328	0.480
Superior Temporal Gyrus; Anterior Division R	0.65	0.22	0.70	0.12	-0.31	-1.4	0.080	0.141	0.78	0.05	0.78	0.05	-0.05	0.1	0.476	0.495
Superior Temporal Gyrus; Posterior Division R	0.78	0.10	0.75	0.08	0.36	1.7	0.049	0.095	0.79	0.05	0.79	0.04	-0.05	-0.5	0.300	0.467
Middle Temporal Gyrus; Anterior Division R	0.73	0.15	0.73	0.08	0.00	0.0	0.494	0.494	0.78	0.06	0.77	0.06	-0.05	1.3	0.101	0.356
Middle Temporal Gyrus; Posterior Division R	0.77	0.06	0.74	0.07	0.45	2.1	0.019	0.048	0.78	0.05	0.76	0.06	-0.41	1.1	0.140	0.368
Inferior Temporal Gyrus; Anterior Division R	0.73	0.15	0.65	0.13	0.56	2.6	0.006	0.020	0.81	0.06	0.78	0.08	0.16	1.6	0.055	0.292
Inferior Temporal Gyrus; Posterior Division R	0.76	0.10	0.70	0.12	0.53	2.5	0.008	0.025	0.79	0.09	0.75	0.07	0.15	2.3	0.012	0.175
Planum Temporale R	0.80	0.07	0.75	0.08	0.64	3.0	0.002	0.011	0.80	0.04	0.78	0.04	0.52	1.4	0.076	0.347
Heschl's Gyrus R	0.75	0.12	0.75	0.09	0.01	0.0	0.486	0.494	0.79	0.07	0.78	0.06	-0.25	0.2	0.408	0.491
Temporal Fusiform Cortex; Anterior Division R	0.55	0.23	0.59	0.16	-0.22	-1.0	0.158	0.237	0.79	0.16	0.78	0.14	-0.26	0.3	0.395	0.491
Temporal Fusiform Cortex; Posterior Division R	0.62	0.18	0.62	0.13	-0.01	-0.1	0.476	0.494	0.77	0.05	0.76	0.07	0.71	1.0	0.156	0.368
Temporal Occipital Fusiform Cortex R	0.70	0.17	0.69	0.12	0.10	0.5	0.322	0.367	0.77	0.06	0.79	0.05	0.22	-1.8	0.035	0.250
Middle Temporal Gyrus; Temporooccipital Part R	0.75	0.11	0.73	0.08	0.19	0.9	0.187	0.261	0.76	0.06	0.77	0.06	0.17	-0.4	0.333	0.480
Inferior Temporal Gyrus; Temporooccipital Part R	0.71	0.14	0.69	0.14	0.20	0.9	0.183	0.258	0.79	0.06	0.78	0.06	-0.20	0.4	0.357	0.480
Occipital Fusiform Gyrus R	0.73	0.11	0.72	0.11	0.12	0.5	0.296	0.347	0.81	0.07	0.83	0.05	0.04	-1.9	0.031	0.250
Supracalcarine Cortex R	0.74	0.14	0.72	0.10	0.15	0.7	0.242	0.321	0.85	0.08	0.86	0.06	0.04	-0.2	0.405	0.491
Cuneal Cortex R	0.78	0.10	0.72	0.12	0.47	2.2	0.016	0.042	0.83	0.07	0.83	0.06	-0.27	0.0	0.500	0.500
Lingual Gyrus R	0.72	0.13	0.69	0.13	0.28	1.3	0.099	0.169	0.82	0.06	0.83	0.06	0.33	-0.9	0.183	0.373
Intracalcarine Cortex R	0.75	0.15	0.72	0.10	0.24	1.1	0.132	0.202	0.83	0.08	0.86	0.07	0.30	-1.8	0.034	0.250
Lateral Occipital Cortex; Superior Division R	0.77	0.04	0.75	0.04	0.58	2.7	0.004	0.019	0.76	0.07	0.78	0.06	0.35	-1.9	0.030	0.250
Lateral Occipital Cortex; Inferior Division R	0.74	0.10	0.73	0.08	0.05	0.2	0.416	0.450	0.80	0.05	0.80	0.05	0.20	-0.1	0.459	0.491
Occipital Pole R	0.75	0.08	0.74	0.07	0.15	0.7	0.251	0.328	0.82	0.09	0.84	0.06	0.17	-1.3	0.091	0.356

Supplementary Table 9. Mean, SD and effect sizes (Cohen's D) of regional betweenness centrality for younger (N = 40) and old (N = 46) adults in fPET and fMRI from the Harvard Oxford Atlas, and t-tests of age group differences.

	fPET								fMRI							
	Younger		Older		Younger vs Older				Younger		Older		Younger vs Older			
	Mean	SD	Mean	SD	Cohen's D	*t*-value	p	p-FDR	Mean	SD	Mean	SD	Cohen's D	*t*-value	p	p-FDR
Frontal Orbital Cortex L	0.011	0.007	0.008	0.005	0.60	2.8	0.003	0.022	0.012	0.009	0.011	0.008	-0.13	0.5	0.315	0.433
Frontal Pole L	0.026	0.013	0.021	0.009	0.53	2.4	0.008	0.043	0.009	0.006	0.010	0.007	0.25	-1.0	0.156	0.373
Frontal Operculum Cortex L	0.005	0.004	0.005	0.004	0.07	0.3	0.379	0.423	0.007	0.006	0.007	0.005	0.23	-0.1	0.480	0.496
Superior Frontal Gyrus L	0.013	0.008	0.008	0.006	0.63	2.9	0.002	0.022	0.008	0.006	0.009	0.007	-0.05	-0.7	0.229	0.389
Middle Frontal Gyrus L	0.015	0.006	0.012	0.007	0.39	1.8	0.039	0.115	0.005	0.004	0.006	0.004	0.17	-1.5	0.071	0.354
Inferior Frontal Gyrus; Pars Triangularis L	0.008	0.005	0.008	0.004	0.01	0.0	0.482	0.490	0.006	0.005	0.006	0.004	0.17	0.6	0.291	0.417
Inferior Frontal Gyrus; pars opercularis L	0.007	0.005	0.006	0.005	0.15	0.7	0.243	0.362	0.007	0.006	0.006	0.005	-0.21	0.7	0.238	0.389
Paracingulate Gyrus L	0.010	0.007	0.006	0.004	0.60	2.8	0.003	0.022	0.007	0.005	0.008	0.005	0.12	-1.3	0.103	0.360
Insular Cortex L	0.004	0.003	0.004	0.003	0.05	0.2	0.410	0.439	0.010	0.006	0.010	0.007	0.10	-0.2	0.414	0.462
Amygdala l	0.004	0.004	0.003	0.002	0.31	1.4	0.077	0.190	0.005	0.006	0.003	0.005	-0.10	1.9	0.030	0.255
Juxtapositional Lobule Cortex - L	0.005	0.006	0.005	0.003	0.13	0.6	0.274	0.392	0.005	0.004	0.007	0.004	-0.06	-1.9	0.031	0.255
Precentral Gyrus L	0.011	0.006	0.010	0.008	0.06	0.3	0.398	0.435	0.010	0.007	0.012	0.007	-0.07	-1.5	0.069	0.354
PostCG l Postcentral Gyrus L	0.009	0.006	0.012	0.008	-0.39	-1.8	0.037	0.115	0.009	0.008	0.010	0.005	-0.22	-0.6	0.260	0.394
Central Opercular Cortex L	0.003	0.003	0.006	0.005	-0.63	-2.9	0.002	0.022	0.008	0.005	0.007	0.004	-0.08	1.1	0.136	0.360
Superior Parietal Lobule L	0.007	0.005	0.006	0.004	0.25	1.1	0.129	0.261	0.007	0.005	0.010	0.005	-0.10	-2.7	0.004	0.136
Supramarginal Gyrus; Anterior Division L	0.005	0.004	0.005	0.004	0.10	0.5	0.318	0.409	0.005	0.005	0.005	0.004	-0.37	-0.5	0.301	0.419
Supramarginal Gyrus; Posterior Division L	0.008	0.004	0.008	0.004	-0.07	-0.3	0.368	0.422	0.009	0.007	0.008	0.004	-0.18	0.3	0.394	0.460
Angular Gyrus L	0.005	0.004	0.006	0.004	-0.24	-1.1	0.138	0.263	0.011	0.009	0.005	0.004	-0.20	3.8	0.000	0.015
Parietal Operculum Cortex L	0.005	0.004	0.006	0.005	-0.29	-1.3	0.091	0.201	0.009	0.006	0.009	0.006	-0.24	0.0	0.498	0.498
Parahippocampal Gyrus; Anterior Division L	0.003	0.003	0.005	0.003	-0.46	-2.1	0.019	0.076	0.007	0.010	0.005	0.005	-0.14	0.9	0.198	0.373
Parahippocampal Gyrus; Posterior Division L	0.002	0.002	0.003	0.003	-0.67	-3.1	0.001	0.022	0.005	0.004	0.005	0.005	-0.14	0.1	0.477	0.496
Hippocampus l	0.005	0.005	0.004	0.003	0.24	1.1	0.131	0.261	0.008	0.005	0.006	0.006	-0.23	1.1	0.130	0.360
Thalamus L	0.002	0.002	0.004	0.003	-0.62	-2.9	0.002	0.022	0.007	0.006	0.006	0.006	-0.02	1.1	0.131	0.360
Caudate L	0.004	0.004	0.007	0.004	-0.61	-2.8	0.003	0.022	0.005	0.004	0.006	0.008	-0.03	-0.7	0.240	0.389
Putamen L	0.009	0.009	0.008	0.006	0.15	0.7	0.249	0.366	0.005	0.004	0.006	0.009	-0.02	-0.3	0.365	0.457
Pallidum L	0.003	0.002	0.005	0.003	-0.79	-3.6	0.000	0.008	0.004	0.004	0.005	0.006	0.04	-1.3	0.100	0.360
Accumbens L	0.002	0.002	0.003	0.003	-0.39	-1.8	0.039	0.115	0.005	0.007	0.004	0.006	0.05	1.0	0.168	0.373
Temporal Pole L	0.006	0.005	0.005	0.003	0.38	1.8	0.041	0.116	0.011	0.007	0.013	0.010	-0.35	-1.3	0.095	0.360
Planum Polare L	0.002	0.002	0.003	0.002	-0.63	-2.9	0.002	0.022	0.006	0.004	0.005	0.004	-0.60	0.8	0.213	0.382
Superior Temporal Gyrus; Anterior Division L	0.002	0.002	0.003	0.002	-0.43	-2.0	0.026	0.095	0.009	0.008	0.008	0.008	-1.20	0.2	0.437	0.468
Superior Temporal Gyrus; Posterior Division L	0.004	0.004	0.005	0.004	-0.42	-1.9	0.028	0.098	0.009	0.006	0.009	0.006	0.29	0.0	0.484	0.496
Middle Temporal Gyrus; Anterior Division L	0.006	0.006	0.004	0.003	0.40	1.9	0.033	0.105	0.007	0.005	0.006	0.005	0.41	0.6	0.271	0.405
Middle Temporal Gyrus; Posterior Division L	0.014	0.010	0.007	0.004	0.89	4.1	0.000	0.004	0.008	0.006	0.008	0.005	0.39	-0.1	0.442	0.468
Inferior Temporal Gyrus; Anterior Division L	0.004	0.003	0.003	0.002	0.10	0.5	0.324	0.409	0.005	0.004	0.005	0.004	0.37	0.2	0.437	0.468
Inferior Temporal Gyrus; Posterior Division L	0.008	0.006	0.006	0.004	0.51	2.4	0.011	0.051	0.010	0.010	0.005	0.004	-0.03	3.1	0.001	0.074
Planum Temporale L	0.010	0.007	0.007	0.004	0.44	2.0	0.023	0.086	0.009	0.006	0.011	0.006	-0.03	-1.8	0.041	0.262
Heschl's Gyrus L	0.006	0.005	0.007	0.004	-0.23	-1.1	0.141	0.263	0.008	0.007	0.009	0.006	0.47	-0.9	0.191	0.373
Temporal Fusiform Cortex; Anterior Division L	0.003	0.003	0.003	0.003	-0.24	-1.1	0.139	0.263	0.005	0.006	0.005	0.005	0.15	0.3	0.367	0.457
Temporal Fusiform Cortex; Posterior Division L	0.004	0.003	0.005	0.004	-0.27	-1.3	0.106	0.230	0.012	0.010	0.011	0.007	0.14	0.2	0.402	0.460
Temporal Occipital Fusiform Cortex L	0.003	0.003	0.005	0.004	-0.63	-2.9	0.002	0.022	0.011	0.009	0.008	0.006	0.16	2.1	0.019	0.220
Middle Temporal Gyrus; Temporooccipital Part L	0.006	0.004	0.006	0.004	0.18	0.8	0.202	0.330	0.009	0.006	0.007	0.004	0.23	2.4	0.009	0.136
Inferior Temporal Gyrus; Temporooccipital Part L	0.004	0.003	0.005	0.004	-0.26	-1.2	0.113	0.240	0.009	0.006	0.007	0.004	0.21	2.5	0.008	0.136
Occipital Fusiform Gyrus L	0.006	0.005	0.007	0.005	-0.14	-0.7	0.253	0.367	0.008	0.010	0.006	0.005	-0.02	1.2	0.121	0.360
Supracalcarine Cortex L	0.004	0.003	0.005	0.003	-0.46	-2.1	0.019	0.076	0.004	0.004	0.004	0.003	0.20	0.4	0.343	0.449
Cuneal Cortex L	0.004	0.004	0.005	0.003	-0.08	-0.4	0.350	0.422	0.006	0.005	0.007	0.005	0.22	-0.4	0.330	0.448
Lingual Gyrus L	0.006	0.005	0.007	0.005	-0.11	-0.5	0.301	0.404	0.006	0.007	0.005	0.005	-0.25	1.0	0.172	0.373
Intracalcarine Cortex L	0.006	0.006	0.006	0.004	0.13	0.6	0.278	0.392	0.004	0.004	0.004	0.004	0.32	-0.3	0.383	0.460
Lateral Occipital Cortex; Superior Division L	0.017	0.009	0.015	0.008	0.29	1.3	0.090	0.201	0.014	0.008	0.012	0.008	0.33	0.9	0.176	0.373
Lateral Occipital Cortex; Inferior Division L	0.008	0.006	0.009	0.006	-0.10	-0.5	0.317	0.409	0.011	0.007	0.009	0.006	0.24	1.3	0.104	0.360
Occipital Pole L	0.011	0.009	0.012	0.007	-0.09	-0.4	0.346	0.421	0.004	0.004	0.005	0.005	0.09	-0.2	0.403	0.460
Frontal Medial Cortex	0.012	0.008	0.010	0.006	0.31	1.4	0.080	0.192	0.007	0.005	0.007	0.007	0.10	-0.6	0.282	0.410
Cingulate Gyrus; Anterior Division	0.009	0.006	0.008	0.006	0.23	1.1	0.144	0.264	0.008	0.006	0.010	0.006	0.12	-1.8	0.038	0.262
Subcallosal Cortex	0.005	0.005	0.004	0.004	0.32	1.5	0.073	0.185	0.007	0.006	0.006	0.007	0.13	0.8	0.225	0.389
Cingulate Gyrus; Posterior Division	0.016	0.010	0.015	0.007	0.12	0.6	0.292	0.400	0.008	0.009	0.009	0.008	0.13	-0.3	0.397	0.460
Precuneous Cortex	0.021	0.010	0.022	0.009	-0.12	-0.6	0.291	0.400	0.010	0.008	0.008	0.005	-0.14	0.9	0.190	0.373
Brain-Stem	0.010	0.008	0.006	0.005	0.67	3.1	0.001	0.022	0.003	0.005	0.004	0.006	0.00	-0.8	0.216	0.382
Frontal Orbital Cortex R	0.011	0.006	0.009	0.005	0.37	1.7	0.047	0.129	0.009	0.008	0.011	0.010	0.00	-1.1	0.147	0.370
Frontal Pole R	0.030	0.012	0.022	0.007	0.86	4.0	0.000	0.004	0.008	0.004	0.009	0.005	0.18	-0.4	0.334	0.448
Frontal Operculum Cortex R	0.006	0.005	0.007	0.005	-0.29	-1.3	0.091	0.201	0.008	0.006	0.007	0.006	-0.03	0.7	0.249	0.389
Superior Frontal Gyrus R	0.011	0.006	0.008	0.005	0.47	2.2	0.016	0.073	0.008	0.005	0.009	0.004	-0.03	-0.9	0.184	0.373
Middle Frontal Gyrus R	0.018	0.007	0.014	0.007	0.55	2.6	0.006	0.037	0.005	0.004	0.006	0.006	0.11	-0.8	0.199	0.373
Inferior Frontal Gyrus; Pars Triangularis R	0.007	0.005	0.009	0.005	-0.37	-1.7	0.047	0.129	0.007	0.005	0.007	0.005	-0.65	0.3	0.398	0.460
Inferior Frontal Gyrus; Pars Opercularis R	0.011	0.005	0.008	0.004	0.61	2.8	0.003	0.022	0.007	0.005	0.006	0.004	-0.67	1.2	0.124	0.360
Paracingulate Gyrus R	0.010	0.006	0.008	0.005	0.50	2.3	0.012	0.056	0.005	0.005	0.008	0.007	-0.25	-2.2	0.017	0.220
Insular Cortex R	0.005	0.005	0.004	0.003	0.17	0.8	0.213	0.333	0.011	0.007	0.012	0.007	-0.27	-0.7	0.235	0.389
Amygdala r	0.005	0.006	0.005	0.004	0.04	0.2	0.433	0.454	0.004	0.004	0.004	0.005	-0.35	0.2	0.408	0.460
Juxtapositional Lobule Cortex R	0.005	0.006	0.005	0.004	0.03	0.1	0.449	0.462	0.006	0.004	0.008	0.005	0.12	-2.4	0.009	0.136
Precentral Gyrus R	0.013	0.007	0.011	0.007	0.22	1.0	0.154	0.276	0.009	0.006	0.010	0.006	0.21	-1.2	0.125	0.360
Postcentral Gyrus R	0.006	0.004	0.008	0.005	-0.41	-1.9	0.029	0.101	0.009	0.006	0.009	0.006	0.18	0.0	0.495	0.498
Central Opercular Cortex R	0.006	0.007	0.007	0.004	-0.08	-0.4	0.357	0.422	0.008	0.009	0.008	0.005	0.01	0.0	0.486	0.496
Superior Parietal Lobule R	0.006	0.005	0.005	0.003	0.24	1.1	0.136	0.263	0.007	0.005	0.010	0.006	-0.20	-1.9	0.031	0.255
Supramarginal Gyrus; Anterior Division R	0.004	0.004	0.004	0.002	0.09	0.4	0.339	0.420	0.006	0.005	0.005	0.004	-0.21	1.1	0.143	0.369
Supramarginal Gyrus; Posterior Division R	0.007	0.006	0.007	0.004	0.03	0.1	0.444	0.462	0.006	0.004	0.006	0.004	0.70	0.4	0.348	0.449
Angular Gyrus R	0.008	0.006	0.008	0.005	0.06	0.3	0.398	0.435	0.006	0.005	0.005	0.004	0.10	1.4	0.084	0.354
Parietal Operculum Cortex R	0.005	0.004	0.005	0.004	-0.07	-0.3	0.374	0.422	0.007	0.006	0.008	0.005	0.09	-0.4	0.343	0.449
Parahippocampal Gyrus; Anterior Division R	0.005	0.005	0.004	0.002	0.33	1.5	0.067	0.174	0.006	0.008	0.008	0.007	0.01	-0.8	0.209	0.381
Parahippocampal Gyrus; Posterior Division R	0.002	0.002	0.003	0.003	-0.51	-2.4	0.010	0.051	0.009	0.009	0.005	0.006	-0.11	2.4	0.009	0.136
Hippocampus R	0.005	0.006	0.004	0.003	0.35	1.6	0.054	0.142	0.007	0.005	0.009	0.008	-0.12	-0.9	0.188	0.373
Thalamus R	0.003	0.003	0.004	0.005	-0.44	-2.0	0.023	0.086	0.009	0.010	0.008	0.006	0.28	0.9	0.193	0.373
Caudate R	0.003	0.003	0.005	0.003	-0.68	-3.1	0.001	0.022	0.007	0.017	0.007	0.007	0.24	0.2	0.423	0.466
Putamen R	0.009	0.008	0.009	0.006	-0.07	-0.3	0.372	0.422	0.006	0.007	0.008	0.009	0.22	-1.0	0.155	0.373
Pallidum R	0.003	0.003	0.003	0.003	-0.18	-0.8	0.209	0.333	0.004	0.005	0.003	0.004	0.35	1.0	0.165	0.373
Accumbens R	0.002	0.002	0.003	0.002	-0.41	-1.9	0.031	0.103	0.004	0.005	0.003	0.004	0.51	0.9	0.178	0.373
Temporal Pole R	0.007	0.007	0.006	0.005	0.07	0.3	0.367	0.422	0.013	0.008	0.014	0.008	0.60	-0.7	0.253	0.389
Planum Polare R	0.003	0.003	0.004	0.005	-0.20	-0.9	0.183	0.312	0.007	0.007	0.007	0.005	0.31	0.3	0.380	0.460
Superior Temporal Gyrus; Anterior Division R	0.002	0.002	0.004	0.002	-0.60	-2.8	0.004	0.022	0.011	0.008	0.010	0.007	0.05	0.4	0.354	0.452
Superior Temporal Gyrus; Posterior Division R	0.006	0.005	0.006	0.004	0.04	0.2	0.429	0.454	0.009	0.006	0.008	0.005	0.05	1.4	0.082	0.354
Middle Temporal Gyrus; Anterior Division R	0.008	0.006	0.005	0.003	0.54	2.5	0.007	0.039	0.008	0.005	0.006	0.003	0.06	1.8	0.036	0.262
Middle Temporal Gyrus; Posterior Division R	0.013	0.008	0.009	0.006	0.62	2.9	0.003	0.022	0.010	0.005	0.008	0.004	0.22	1.7	0.044	0.262
Inferior Temporal Gyrus; Anterior Division R	0.006	0.005	0.005	0.003	0.19	0.9	0.185	0.312	0.005	0.003	0.006	0.005	0.20	-1.2	0.126	0.360
Inferior Temporal Gyrus; Posterior Division R	0.007	0.006	0.006	0.005	0.16	0.8	0.224	0.345	0.006	0.006	0.008	0.008	-0.39	-1.1	0.136	0.360
Planum Temporale R	0.008	0.005	0.008	0.005	-0.05	-0.2	0.404	0.437	0.009	0.006	0.013	0.011	-0.09	-2.0	0.023	0.242
Heschl's Gyrus R	0.006	0.005	0.006	0.004	0.00	0.0	0.491	0.491	0.008	0.007	0.009	0.008	-0.10	-0.2	0.426	0.466
Temporal Fusiform Cortex; Anterior Division R	0.003	0.004	0.004	0.003	-0.16	-0.7	0.228	0.346	0.003	0.003	0.004	0.004	0.08	-1.4	0.079	0.354
Temporal Fusiform Cortex; Posterior Division R	0.006	0.007	0.005	0.003	0.12	0.5	0.294	0.400	0.013	0.008	0.011	0.007	-0.41	1.2	0.123	0.360
Temporal Occipital Fusiform Cortex R	0.005	0.006	0.005	0.004	-0.01	0.0	0.486	0.490	0.011	0.008	0.010	0.008	-0.46	0.2	0.408	0.460
Middle Temporal Gyrus; Temporooccipital Part R	0.007	0.005	0.007	0.004	0.09	0.4	0.341	0.420	0.012	0.008	0.010	0.008	-0.05	1.4	0.081	0.354
Inferior Temporal Gyrus; Temporooccipital Part R	0.006	0.006	0.005	0.003	0.20	0.9	0.185	0.312	0.008	0.005	0.007	0.005	0.00	1.5	0.074	0.354
Occipital Fusiform Gyrus R	0.007	0.007	0.007	0.005	0.10	0.5	0.323	0.409	0.006	0.008	0.005	0.004	0.00	0.7	0.248	0.389
Supracalcarine Cortex R	0.006	0.004	0.005	0.004	0.17	0.8	0.214	0.333	0.002	0.002	0.004	0.003	-0.19	-1.7	0.044	0.262
Cuneal Cortex R	0.005	0.004	0.006	0.004	-0.18	-0.8	0.202	0.330	0.007	0.007	0.006	0.006	-0.40	0.5	0.300	0.419
Lingual Gyrus R	0.005	0.004	0.007	0.006	-0.25	-1.1	0.129	0.261	0.006	0.004	0.005	0.004	-0.43	1.2	0.119	0.360
Intracalcarine Cortex R	0.006	0.003	0.005	0.003	0.07	0.3	0.367	0.422	0.005	0.004	0.004	0.004	-0.41	1.2	0.119	0.360
Lateral Occipital Cortex; Superior Division R	0.014	0.007	0.016	0.008	-0.21	-1.0	0.168	0.297	0.011	0.006	0.010	0.006	-0.02	0.7	0.248	0.389
Lateral Occipital Cortex; Inferior Division R	0.010	0.008	0.009	0.006	0.11	0.5	0.309	0.409	0.009	0.009	0.008	0.006	-0.02	0.8	0.200	0.373
Occipital Pole R	0.014	0.011	0.011	0.007	0.30	1.4	0.082	0.192	0.005	0.006	0.005	0.005	-0.29	0.6	0.280	0.410

Supplementary Table 10. Mean, SD and effect sizes (Cohen's D) of regional betweenness centrality for younger (N = 40) and older (N = 46) adults in fPET and fMRI from the Harvard Oxford Atlas, and t-tests of age group differences.

	fPET								fMRI							
	Younger		Older		Younger vs Older				Younger		Older		Younger vs Older			
	Mean	SD	Mean	SD	Cohen's D	*t*-value	p	p-FDR	Mean	SD	Mean	SD	Cohen's D	*t*-value	p	p-FDR
Frontal Orbital Cortex L	44.28	13.57	37.43	13.00	0.52	2.4	0.010	0.032	39.00	12.37	32.35	15.24	0.10	2.2	0.015	0.057
Frontal Pole L	68.63	8.77	61.76	10.48	0.71	3.3	0.001	0.006	35.20	13.42	32.89	12.98	0.11	0.8	0.210	0.329
Frontal Operculum Cortex L	27.05	12.22	28.41	12.01	-0.11	-0.5	0.302	0.410	30.63	11.84	28.87	11.23	-0.22	0.7	0.241	0.347
Superior Frontal Gyrus L	50.20	15.53	39.35	16.10	0.69	3.2	0.001	0.006	31.80	13.46	31.54	12.12	-0.01	0.1	0.463	0.487
Middle Frontal Gyrus L	55.45	8.63	47.98	12.22	0.70	3.2	0.001	0.006	26.05	12.63	27.83	12.30	-0.01	-0.7	0.256	0.348
Inferior Frontal Gyrus; Pars Triangularis L	43.10	13.10	39.24	11.12	0.32	1.5	0.072	0.147	28.35	12.86	26.41	9.98	-0.16	0.8	0.217	0.333
Inferior Frontal Gyrus; pars opercularis L	37.88	13.99	31.52	12.82	0.48	2.2	0.015	0.049	29.50	10.70	27.67	11.47	-0.32	0.8	0.225	0.333
Paracingulate Gyrus L	45.48	10.48	33.43	12.43	1.04	4.8	0.000	0.000	28.00	13.22	30.11	12.34	-0.30	-0.8	0.223	0.333
Insular Cortex L	20.28	10.54	21.15	9.65	-0.09	-0.4	0.344	0.414	36.00	12.63	36.54	10.11	0.12	-0.2	0.413	0.460
Amygdala l	15.00	11.79	16.72	8.76	-0.17	-0.8	0.221	0.349	25.60	12.81	17.50	12.78	0.15	2.9	0.002	0.014
Juxtapositional Lobule Cortex - L	26.85	13.62	26.93	12.59	-0.01	0.0	0.488	0.493	30.30	12.68	36.20	10.10	0.17	-2.4	0.009	0.043
Precentral Gyrus L	46.28	16.53	40.91	15.80	0.33	1.5	0.064	0.142	38.83	16.48	45.52	9.99	-0.27	-2.3	0.012	0.049
PostCG l Postcentral Gyrus L	43.43	15.64	44.35	15.80	-0.06	-0.3	0.393	0.439	35.50	14.58	44.15	9.04	-0.05	-3.4	0.001	0.005
Central Opercular Cortex L	20.93	12.94	28.76	12.83	-0.61	-2.8	0.003	0.014	38.03	12.49	37.33	11.97	-0.04	0.3	0.396	0.451
Superior Parietal Lobule L	37.78	16.33	34.17	12.87	0.25	1.1	0.128	0.219	31.98	12.63	41.28	9.63	0.41	-3.9	0.000	0.003
Supramarginal Gyrus; Anterior Division L	34.88	13.80	28.15	12.07	0.52	2.4	0.009	0.031	29.85	11.58	31.80	9.80	-0.41	-0.8	0.200	0.320
Supramarginal Gyrus; Posterior Division L	41.25	11.81	37.57	11.35	0.32	1.5	0.072	0.147	34.30	13.30	35.72	12.72	-0.39	-0.5	0.308	0.388
Angular Gyrus L	33.68	12.86	34.15	11.49	-0.04	-0.2	0.428	0.468	35.60	12.65	25.76	12.75	-0.32	3.6	0.000	0.004
Parietal Operculum Cortex L	28.33	14.59	29.09	10.75	-0.06	-0.3	0.391	0.439	40.60	10.82	40.80	13.14	-0.14	-0.1	0.469	0.487
Parahippocampal Gyrus; Anterior Division L	14.45	9.49	18.57	8.42	-0.46	-2.1	0.018	0.054	27.13	14.33	25.93	12.94	-0.19	0.4	0.343	0.409
Parahippocampal Gyrus; Posterior Division L	11.18	6.50	17.00	9.66	-0.70	-3.2	0.001	0.006	23.63	12.72	23.89	13.77	0.24	-0.1	0.463	0.487
Hippocampus l	17.23	12.68	17.91	10.84	-0.06	-0.3	0.393	0.439	31.38	13.28	24.59	15.22	-0.58	2.2	0.016	0.057
Thalamus L	14.80	9.45	23.02	11.04	-0.80	-3.7	0.000	0.003	27.03	15.29	24.35	15.43	-0.57	0.8	0.211	0.329
Caudate L	19.73	11.53	29.72	13.14	-0.80	-3.7	0.000	0.003	21.63	12.89	21.76	12.51	-0.11	0.0	0.480	0.488
Putamen L	29.43	14.48	34.15	14.50	-0.33	-1.5	0.068	0.146	23.80	13.47	21.22	12.77	0.06	0.9	0.182	0.307
Pallidum L	12.43	7.45	21.11	10.15	-0.97	-4.5	0.000	0.000	22.03	14.52	20.20	14.79	0.07	0.6	0.283	0.366
Accumbens L	13.20	9.02	21.65	9.86	-0.89	-4.1	0.000	0.001	19.25	15.16	15.39	14.51	0.81	1.2	0.116	0.223
Temporal Pole L	24.13	13.39	21.85	10.39	0.19	0.9	0.189	0.303	40.80	10.65	36.78	11.73	0.00	1.7	0.051	0.132
Planum Polare L	12.65	8.08	19.00	9.02	-0.74	-3.4	0.000	0.005	32.50	12.01	29.70	14.23	0.00	1.0	0.165	0.282
Superior Temporal Gyrus; Anterior Division L	15.23	10.33	18.52	8.33	-0.35	-1.6	0.053	0.122	34.90	13.34	34.50	15.01	0.18	0.1	0.449	0.485
Superior Temporal Gyrus; Posterior Division L	23.38	13.93	28.72	10.72	-0.43	-2.0	0.024	0.067	36.83	13.21	37.22	12.49	0.01	-0.1	0.444	0.485
Middle Temporal Gyrus; Anterior Division L	22.30	12.83	20.93	9.05	0.12	0.6	0.283	0.395	30.75	13.69	29.41	12.49	0.01	0.5	0.319	0.397
Middle Temporal Gyrus; Posterior Division L	46.13	13.93	35.96	14.04	0.73	3.4	0.001	0.005	35.33	14.16	30.78	10.71	0.24	1.7	0.047	0.125
Inferior Temporal Gyrus; Anterior Division L	19.73	11.35	18.76	8.54	0.10	0.4	0.327	0.414	29.90	11.71	23.85	13.06	0.24	2.2	0.014	0.053
Inferior Temporal Gyrus; Posterior Division L	36.58	12.81	27.65	12.88	0.69	3.2	0.001	0.006	36.53	13.57	25.35	12.49	0.25	4.0	0.000	0.003
Planum Temporale L	43.05	12.38	37.89	10.63	0.45	2.1	0.020	0.058	39.13	12.98	42.93	12.78	-0.15	-1.4	0.087	0.181
Heschl's Gyrus L	30.60	12.90	33.87	11.17	-0.27	-1.3	0.106	0.189	32.70	12.43	34.72	14.33	-0.08	-0.7	0.245	0.347
Temporal Fusiform Cortex; Anterior Division L	13.83	9.57	18.54	10.62	-0.47	-2.2	0.017	0.054	23.75	16.07	23.78	12.75	-0.06	0.0	0.496	0.496
Temporal Fusiform Cortex; Posterior Division L	19.85	9.37	22.67	9.95	-0.29	-1.3	0.091	0.178	38.35	15.11	35.98	9.92	-0.28	0.9	0.193	0.320
Temporal Occipital Fusiform Cortex L	19.50	10.56	25.41	9.03	-0.60	-2.8	0.003	0.014	36.25	12.40	34.61	10.76	0.21	0.7	0.256	0.348
Middle Temporal Gyrus; Temporooccipital Part L	30.68	12.73	30.72	11.99	0.00	0.0	0.494	0.494	38.03	13.53	32.63	10.15	0.23	2.1	0.019	0.062
Inferior Temporal Gyrus; Temporooccipital Part L	24.18	12.04	25.67	9.34	-0.14	-0.6	0.259	0.376	36.08	12.23	30.17	10.60	-0.28	2.4	0.009	0.043
Occipital Fusiform Gyrus L	30.65	16.60	34.70	13.31	-0.27	-1.3	0.107	0.189	29.58	11.94	33.74	10.15	0.17	-1.7	0.042	0.122
Supracalcarine Cortex L	25.45	9.50	27.87	10.66	-0.24	-1.1	0.136	0.230	24.60	11.43	33.28	9.68	0.18	-3.8	0.000	0.003
Cuneal Cortex L	25.18	13.49	26.83	10.96	-0.14	-0.6	0.267	0.377	27.80	11.57	37.28	11.33	0.03	-3.8	0.000	0.003
Lingual Gyrus L	27.95	12.90	29.93	13.72	-0.15	-0.7	0.247	0.368	28.68	11.01	34.26	9.29	-0.01	-2.6	0.006	0.033
Intracalcarine Cortex L	31.48	15.30	30.78	13.16	0.05	0.2	0.411	0.454	23.80	11.74	32.54	9.79	-0.01	-3.8	0.000	0.003
Lateral Occipital Cortex; Superior Division L	56.88	10.17	52.76	12.34	0.36	1.7	0.049	0.121	37.83	11.14	39.37	10.62	0.13	-0.7	0.256	0.348
Lateral Occipital Cortex; Inferior Division L	33.18	17.22	38.28	14.40	-0.32	-1.5	0.069	0.146	38.20	11.90	38.30	11.76	-0.03	0.0	0.484	0.488
Occipital Pole L	41.60	16.29	44.07	16.33	-0.15	-0.7	0.243	0.368	23.78	9.30	30.98	9.47	-0.03	-3.5	0.000	0.004
Frontal Medial Cortex	44.60	15.99	39.63	12.70	0.35	1.6	0.056	0.126	29.23	12.48	27.80	13.34	0.03	0.5	0.307	0.388
Cingulate Gyrus; Anterior Division	42.80	14.40	34.30	15.65	0.56	2.6	0.005	0.021	28.88	12.52	34.39	11.15	0.67	-2.2	0.017	0.059
Subcallosal Cortex	27.50	15.87	24.35	11.13	0.23	1.1	0.142	0.236	26.78	16.09	24.09	16.75	1.19	0.8	0.226	0.333
Cingulate Gyrus; Posterior Division	54.88	12.82	54.76	10.69	0.01	0.0	0.482	0.491	25.50	14.36	29.41	11.95	-0.38	-1.4	0.086	0.181
Precuneous Cortex	62.65	12.27	63.89	8.90	-0.12	-0.5	0.295	0.406	25.75	12.31	34.76	11.77	-0.19	-3.5	0.000	0.004
Brain-Stem	41.30	15.99	32.13	15.03	0.59	2.7	0.004	0.015	16.10	13.84	19.39	14.47	-0.21	-1.1	0.143	0.257
Frontal Orbital Cortex R	46.63	13.98	41.48	11.74	0.40	1.9	0.033	0.091	35.90	12.33	31.59	12.79	0.07	1.6	0.058	0.140
Frontal Pole R	71.75	6.84	65.41	9.03	0.78	3.6	0.000	0.003	36.10	14.67	30.24	11.47	0.05	2.1	0.020	0.064
Frontal Operculum Cortex R	32.38	11.76	35.57	10.80	-0.28	-1.3	0.097	0.183	32.35	11.78	28.48	12.00	0.07	1.5	0.068	0.153
Superior Frontal Gyrus R	48.30	13.63	40.91	14.43	0.53	2.4	0.009	0.031	34.00	12.20	33.83	11.54	0.45	0.1	0.473	0.487
Middle Frontal Gyrus R	60.63	11.40	52.54	15.01	0.60	2.8	0.003	0.014	27.33	13.73	26.50	11.29	0.52	0.3	0.380	0.438
Inferior Frontal Gyrus; Pars Triangularis R	42.03	10.33	40.83	11.49	0.11	0.5	0.307	0.412	33.93	12.76	29.22	10.10	0.66	1.9	0.030	0.091
Inferior Frontal Gyrus; Pars Opercularis R	48.85	8.82	40.00	10.45	0.91	4.2	0.000	0.001	33.40	13.46	27.07	10.50	0.53	2.4	0.008	0.041
Paracingulate Gyrus R	48.08	12.11	38.85	11.33	0.79	3.6	0.000	0.003	25.23	12.70	29.67	11.03	0.25	-1.7	0.043	0.122
Insular Cortex R	24.63	10.88	23.61	10.66	0.09	0.4	0.332	0.414	39.83	9.34	39.98	10.39	0.38	-0.1	0.472	0.487
Amygdala r	20.05	14.38	19.61	10.28	0.04	0.2	0.435	0.470	22.90	10.82	19.28	12.72	0.09	1.4	0.081	0.176
Juxtapositional Lobule Cortex R	25.70	15.05	25.46	13.02	0.02	0.1	0.468	0.483	31.45	11.86	38.15	8.88	-0.10	-3.0	0.002	0.013
Precentral Gyrus R	48.60	12.19	42.91	15.92	0.40	1.8	0.035	0.092	41.85	12.83	43.85	8.62	-0.09	-0.9	0.197	0.320
Postcentral Gyrus R	34.23	13.01	35.33	16.64	-0.07	-0.3	0.368	0.429	39.38	10.91	41.78	9.68	0.20	-1.1	0.141	0.257
Central Opercular Cortex R	28.43	14.55	33.11	11.25	-0.36	-1.7	0.048	0.121	38.28	10.97	39.65	11.03	-0.06	-0.6	0.282	0.366
Superior Parietal Lobule R	29.55	15.76	28.28	13.33	0.09	0.4	0.344	0.414	33.35	10.32	38.35	11.24	-0.07	-2.1	0.018	0.061
Supramarginal Gyrus; Anterior Division R	26.15	14.04	24.46	10.58	0.14	0.6	0.263	0.377	33.18	11.07	30.78	10.57	0.20	1.0	0.154	0.268
Supramarginal Gyrus; Posterior Division R	34.13	13.41	35.33	12.10	-0.09	-0.4	0.332	0.414	32.10	14.28	30.87	11.51	0.27	0.4	0.330	0.405
Angular Gyrus R	37.73	13.62	38.83	14.17	-0.08	-0.4	0.358	0.421	28.70	14.02	25.04	12.41	0.30	1.3	0.101	0.199
Parietal Operculum Cortex R	24.48	12.54	25.48	11.71	-0.08	-0.4	0.351	0.418	36.23	12.09	39.54	11.60	-0.05	-1.3	0.099	0.198
Parahippocampal Gyrus; Anterior Division R	14.53	8.95	18.41	8.28	-0.45	-2.1	0.020	0.058	28.25	12.81	29.35	12.94	-0.13	-0.4	0.347	0.409
Parahippocampal Gyrus; Posterior Division R	11.68	8.44	16.57	8.34	-0.58	-2.7	0.004	0.017	26.53	14.39	24.61	13.66	-0.11	0.6	0.264	0.350
Hippocampus R	17.53	12.54	19.50	12.62	-0.16	-0.7	0.235	0.361	29.65	12.95	31.61	15.16	-0.39	-0.6	0.262	0.350
Thalamus R	15.83	11.47	22.39	13.41	-0.52	-2.4	0.009	0.031	29.10	15.23	28.15	13.06	0.16	0.3	0.378	0.438
Caudate R	16.60	10.45	27.72	11.49	-1.01	-4.7	0.000	0.000	20.73	11.99	24.61	14.95	0.15	-1.3	0.096	0.196
Putamen R	26.88	15.77	36.33	14.26	-0.63	-2.9	0.002	0.011	23.43	13.14	24.02	12.20	-0.06	-0.2	0.414	0.460
Pallidum R	12.00	8.16	17.76	9.73	-0.64	-2.9	0.002	0.011	20.55	13.90	17.26	13.30	0.19	1.1	0.133	0.247
Accumbens R	11.93	6.73	18.30	8.63	-0.82	-3.8	0.000	0.003	17.63	13.81	17.00	13.69	0.24	0.2	0.417	0.460
Temporal Pole R	28.25	13.28	27.93	15.00	0.02	0.1	0.459	0.483	44.33	10.72	38.98	12.61	-0.17	2.1	0.019	0.062
Planum Polare R	19.73	11.65	21.48	10.29	-0.16	-0.7	0.230	0.359	36.13	11.20	32.24	13.54	-0.23	1.4	0.077	0.170
Superior Temporal Gyrus; Anterior Division R	15.85	10.21	22.74	9.41	-0.70	-3.3	0.001	0.006	42.93	13.16	39.96	13.25	-0.22	1.0	0.151	0.266
Superior Temporal Gyrus; Posterior Division R	34.48	13.11	33.20	11.03	0.11	0.5	0.312	0.414	42.48	12.03	36.30	12.17	-0.09	2.4	0.010	0.046
Middle Temporal Gyrus; Anterior Division R	31.10	12.28	27.98	8.45	0.30	1.4	0.084	0.169	36.23	10.95	29.61	9.83	0.15	3.0	0.002	0.014
Middle Temporal Gyrus; Posterior Division R	47.73	14.07	38.72	13.63	0.65	3.0	0.002	0.010	40.55	11.26	32.80	10.39	0.15	3.3	0.001	0.005
Inferior Temporal Gyrus; Anterior Division R	27.43	13.65	23.30	9.59	0.35	1.6	0.053	0.122	31.23	13.31	26.98	12.31	-0.19	1.5	0.064	0.148
Inferior Temporal Gyrus; Posterior Division R	31.50	11.85	28.41	11.66	0.26	1.2	0.114	0.198	31.98	13.04	28.46	14.79	-0.18	1.2	0.124	0.235
Planum Temporale R	37.10	12.03	37.39	9.72	-0.03	-0.1	0.451	0.483	40.88	12.68	45.17	10.90	-0.16	-1.7	0.047	0.125
Heschl's Gyrus R	28.35	13.09	32.50	9.49	-0.37	-1.7	0.047	0.121	34.83	12.74	33.57	13.99	0.06	0.4	0.333	0.405
Temporal Fusiform Cortex; Anterior Division R	12.45	8.20	18.09	9.45	-0.63	-2.9	0.002	0.011	22.55	14.11	23.72	12.01	0.25	-0.4	0.340	0.409
Temporal Fusiform Cortex; Posterior Division R	20.03	10.83	22.33	9.56	-0.23	-1.0	0.149	0.243	39.48	10.83	33.93	11.57	0.28	2.3	0.013	0.051
Temporal Occipital Fusiform Cortex R	21.83	10.96	24.76	9.95	-0.28	-1.3	0.098	0.183	34.80	13.62	39.39	12.80	-0.46	-1.6	0.056	0.137
Middle Temporal Gyrus; Temporooccipital Part R	33.13	13.13	33.33	11.09	-0.02	-0.1	0.469	0.483	40.98	13.88	36.07	14.23	-0.16	1.6	0.055	0.137
Inferior Temporal Gyrus; Temporooccipital Part R	24.18	11.44	25.15	10.05	-0.09	-0.4	0.337	0.414	36.10	10.85	29.85	10.56	-0.15	2.7	0.004	0.024
Occipital Fusiform Gyrus R	31.35	14.82	33.28	12.72	-0.14	-0.7	0.258	0.376	25.78	9.78	33.54	10.14	0.05	-3.6	0.000	0.004
Supracalcarine Cortex R	32.80	13.28	28.67	9.85	0.36	1.6	0.051	0.122	20.83	8.95	31.26	12.03	-0.52	-4.5	0.000	0.001
Cuneal Cortex R	29.78	12.77	30.59	10.74	-0.07	-0.3	0.375	0.432	28.18	12.19	36.43	11.04	-0.49	-3.3	0.001	0.005
Lingual Gyrus R	25.93	12.63	29.50	13.24	-0.28	-1.3	0.103	0.188	27.93	9.37	35.13	10.68	-0.25	-3.3	0.001	0.005
Intracalcarine Cortex R	31.28	12.95	30.20	11.40	0.09	0.4	0.341	0.414	24.85	12.32	31.07	10.10	0.00	-2.6	0.006	0.033
Lateral Occipital Cortex; Superior Division R	53.73	13.04	54.91	11.26	-0.10	-0.5	0.326	0.414	34.15	9.62	37.52	10.31	0.00	-1.6	0.061	0.144
Lateral Occipital Cortex; Inferior Division R	36.18	16.07	40.52	14.73	-0.28	-1.3	0.097	0.183	36.53	10.05	38.04	10.20	0.01	-0.7	0.245	0.347
Occipital Pole R	44.68	17.49	44.39	14.79	0.02	0.1	0.468	0.483	24.83	11.80	28.87	9.88	-0.41	-1.7	0.044	0.122

Supplementary Table 11. Regression analyses predicting cognitive performance from fPET and fMRI whole brain graph metrics from the Harvard Oxford Atlas in the whole sample (N = 86). The ANOVA results for the overall models are shown in the top three rows, and the standardized beta weights for each predictor in subsequent rows. Significant beta weights are indicated at *p < 0.05; and **p < 0.001.

	HVLT: Delayed Recall	HVLT: Discrim. Index	Digit Span: Forward	Digit Span: Back	Category Switch: % Trials	Category Switch: RT	Dig Sub: Number Correct	Dig Sub: Sec Per Correct	Stop Signal: Pro Reac	Stop Signal: RT
ANOVA F	1.2	3.1	0.3	1.6	2.6	1.8	2.5	0.6	1.5	1.9
ANOVA p	0.341	0.010	0.936	0.167	0.023	0.106	0.026	0.699	0.181	0.089
Variance Explained	8%	19%	2%	11%	17%	12%	17%	5%	10%	13%
fPET: Global Efficiency	0.16	0.73*	0.12	0.12	0.26	-0.09	-0.07	-0.39	-0.29	0.30
fPET: Local Efficiency	0.35	0.83**	0.00	-0.08	0.41	0.02	0.19	-0.29	0.00	0.47*
fPET: Betweenness Centrality	0.01	-0.14	-0.04	0.05	-0.09	0.17	0.26	0.17	0.32*	0.05
fMRI: Global Efficiency	-0.25	-0.16	0.09	0.17	-0.30	0.07	-0.18	-0.13	-0.01	-0.16
fMRI: Local Efficiency	-0.35	-0.05	-0.10	-0.24	-0.39*	-0.40	-0.37	-0.06	-0.07	-0.28
fMRI: Betweenness Centrality	0.11	0.03	0.12	0.31	0.33*	0.35*	0.34*	0.07	0.04	-0.12

Discrim = discrimination; Dig Sub = Digit Substitution; Pro React = probability of reacting.

Supplementary Table 12. Regression analyses predicting cognitive performance from fPET and fMRI whole brain graph metrics from the Harvard Oxford Atlas and age group in the whole sample (N = 86). The ANOVA results for the overall models are shown in the top three rows, and the standardized beta weights for each predictor in subsequent rows. Significant beta weights are indicated at *p < 0.05; and **p < 0.001

	HVLT: Delayed Recall	HVLT: Discrim. Index	Digit Span: Forward	Digit Span: Back	Category Switch: % Trials	Category Switch: RT	Dig Sub: Number Correct	Dig Sub: Sec Per Correct	Stop Signal: Pro Reac	Stop Signal: RT
ANOVA F	2.4	4.7	0.3	1.7	2.6	6.3	17.2	0.9	1.4	2.3
ANOVA p	0.028	<.001	0.948	0.113	0.018	<.001	<.001	0.529	0.230	0.038
Variance Explained	18%	30%	3%	14%	19%	36%	61%	8%	11%	17%
fPET: Global Efficiency	0.09	0.66*	0.13	0.08	0.23	-0.19	-0.21	-0.42	-0.31	0.25
fPET: Local Efficiency	0.20	0.67*	0.03	-0.16	0.33	-0.21	-0.14	-0.38	-0.04	0.37
fPET: Betweenness Centrality	-0.09	-0.24	-0.02	-0.01	-0.14	0.02	0.06	0.12	0.29*	-0.02
fMRI: Global Efficiency	-0.24	-0.16	0.09	0.17	-0.29	0.08	-0.14	-0.12	-0.01	-0.15
fMRI: Local Efficiency	-0.27	0.04	-0.12	-0.20	-0.35	-0.27	-0.15	0.00	-0.05	-0.22
fMRI: Betweenness Centrality	0.01	-0.07	0.15	0.26	0.28	0.20	0.11	0.01	0.01	-0.19
Age Group	-0.35*	-0.37*	0.08	-0.19	-0.17	-0.55*	-0.75**	-0.19	-0.08	-0.23

Discrim = discrimination; Dig Sub = Digit Substitution; Pro React = probability of reacting.

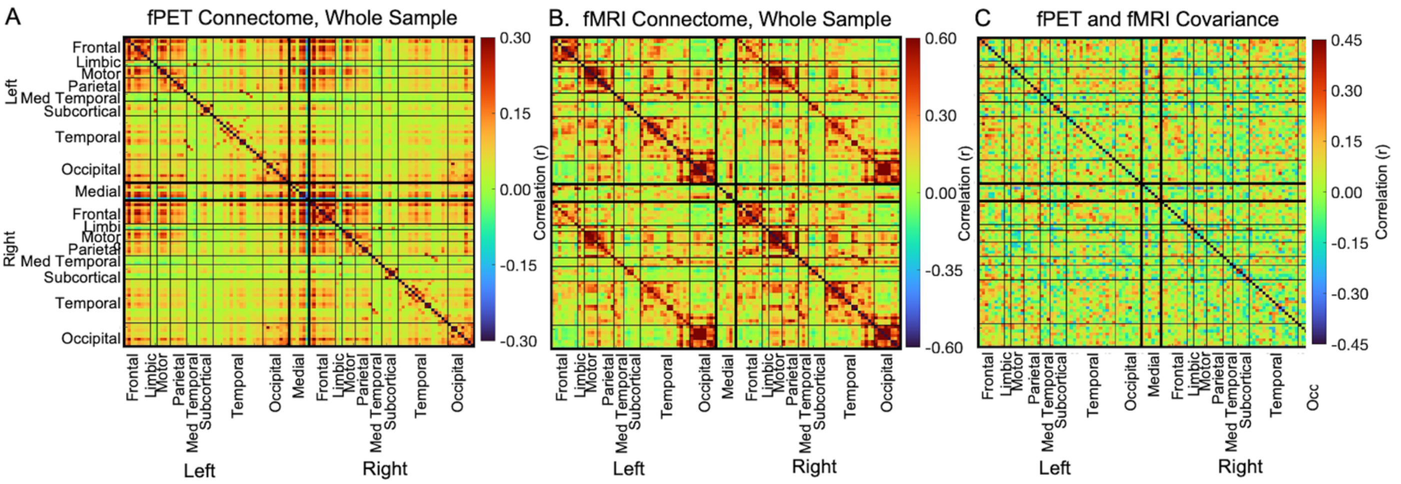

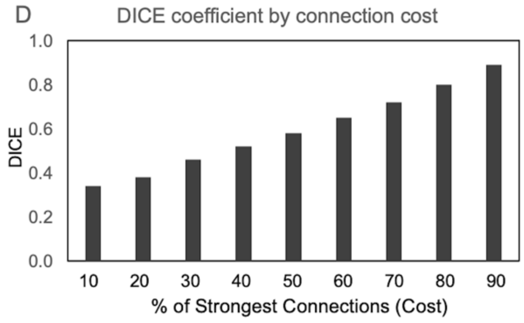

E Descriptive Statistics of Connectome Connections

	Min	Max	Mean	SD
fPET	-0.24	0.50	0.06	0.06
fMRI	-0.21	0.88	0.14	0.16

Supplementary Figure 1. fPET and fMRI connectomes and covariance among the whole sample (N = 86) derived from the Harvard Oxford Atlas parcellation. (A) fPET connectome, (B) fMRI connectome,(C) across-subject covariance of fPET and fMRI connectomes, (D) Dice-Sørensen coefficient (DICE coefficient) of fPET and fMRI connectome similarity at connection costs, and (E) descriptive statistics and z-test of modality differences.

It is noteworthy that the maximum connections in the fPET connectomes derived from the Harvard Oxford Atlas (Supplementary Figure 1) were higher than in the Schaefer (max r = .50) but were similar for the fMRI connectomes (max r = .88). The DICE coefficient was .58 for the top 50% of connections and .34 at top 10% of edges.

The fPET connectome has a relatively homogenous connection strength (SD = .06) within and between networks, mostly in the order of *r* = .20 to 0.40 (Supplementary Figure 1A). In contrast, although not statistically significant, the fMRI connectome is more heterogenous in strength (SD = .16). The highest correlations in the fMRI are larger than the fPET connections, particularly the within-lobe network connections (Supplementary Figure 1B). The strongest covariance for appears to be in between-lobe connections, particularly for regions between the frontal, motor, parietal and occipital cortices (Supplementary Figure 1C).

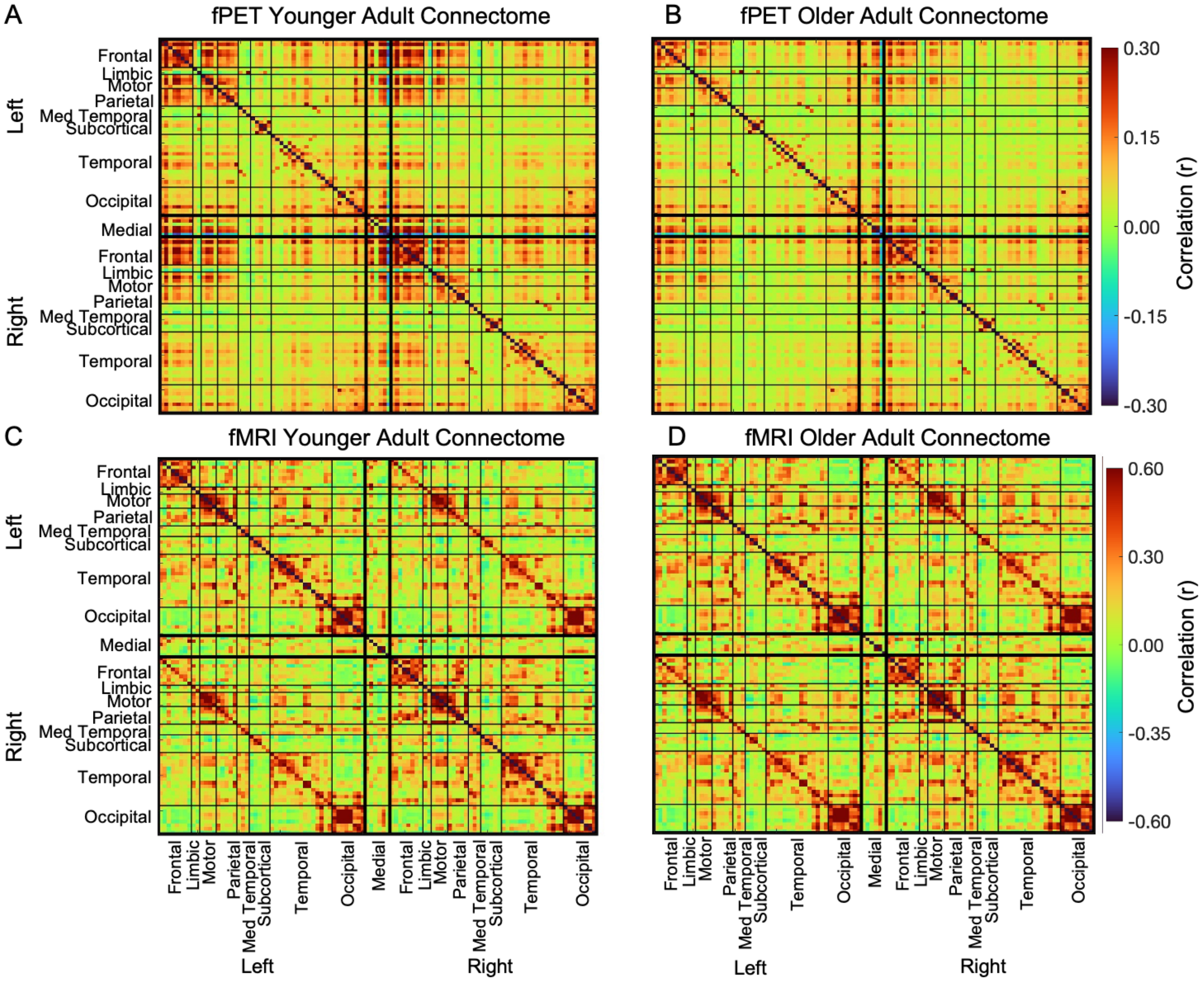

Supplementary Figure 2. fPET and fMRI connectivity for younger and older adults from the Harvard Oxford Atlas parcellation. (A) Younger (N = 40) and (B) older (N = 46) adult fPET metabolic connectomes, and (C) younger (N = 40) and (D) older (N = 46) adult fMRI connectomes.

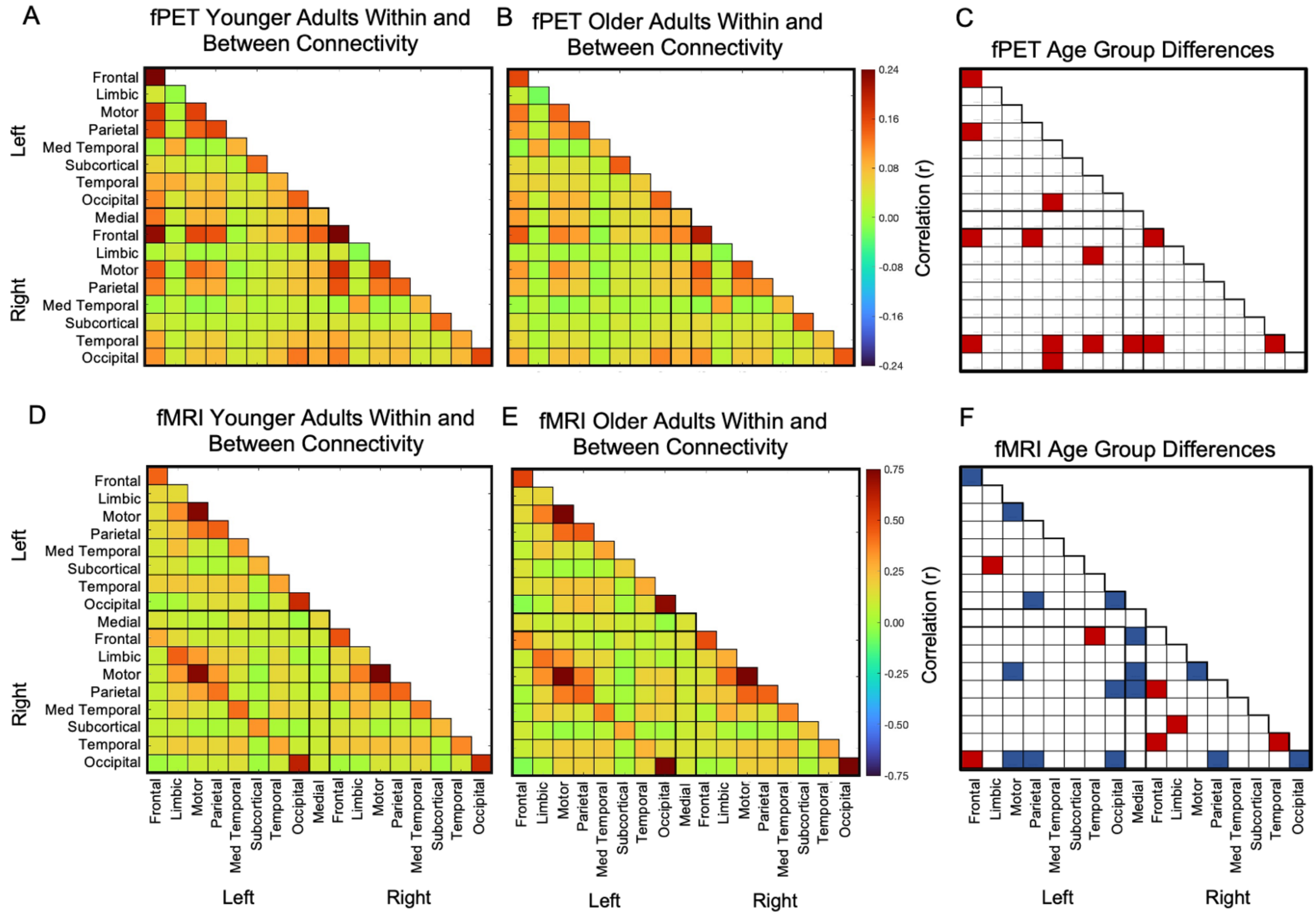

Supplementary Figure 3. Within and between connectivity averaging the nodes of the Harvard Oxford Alas within the lobes for (A) younger (N = 40) and (B) older (N = 46) adults using fPET, and (D) Younger (N = 40) and (E) older (N = 46) adults in fMRI. Within connectivity is shown in the diagonal cells and between connectivity on the off-diagonal cells of each matrix. (significance test (*t*-test, df = 84) of younger (N = 40) vs older (N = 46) adults, with shaded cells indicating a statistically significant age group differences at p-FDR<.05 ((C) and (F)). Red shading younger > older; blue sharing older > younger.

The younger adult fPET within-network connectivity Younger mean = .12, SD = .07 and between-network mean = .06, SD = .04. Older adult fPET within-network mean = .10, SD = .06 and between-network mean = .04, SD = .04. Younger adult fMRI within-network mean = .40, SD = .18 and between-network mean = .14, SD = .15. Older adult fMRI within-network mean = .43, SD = .19 and between-network mean = .15, SD = .15

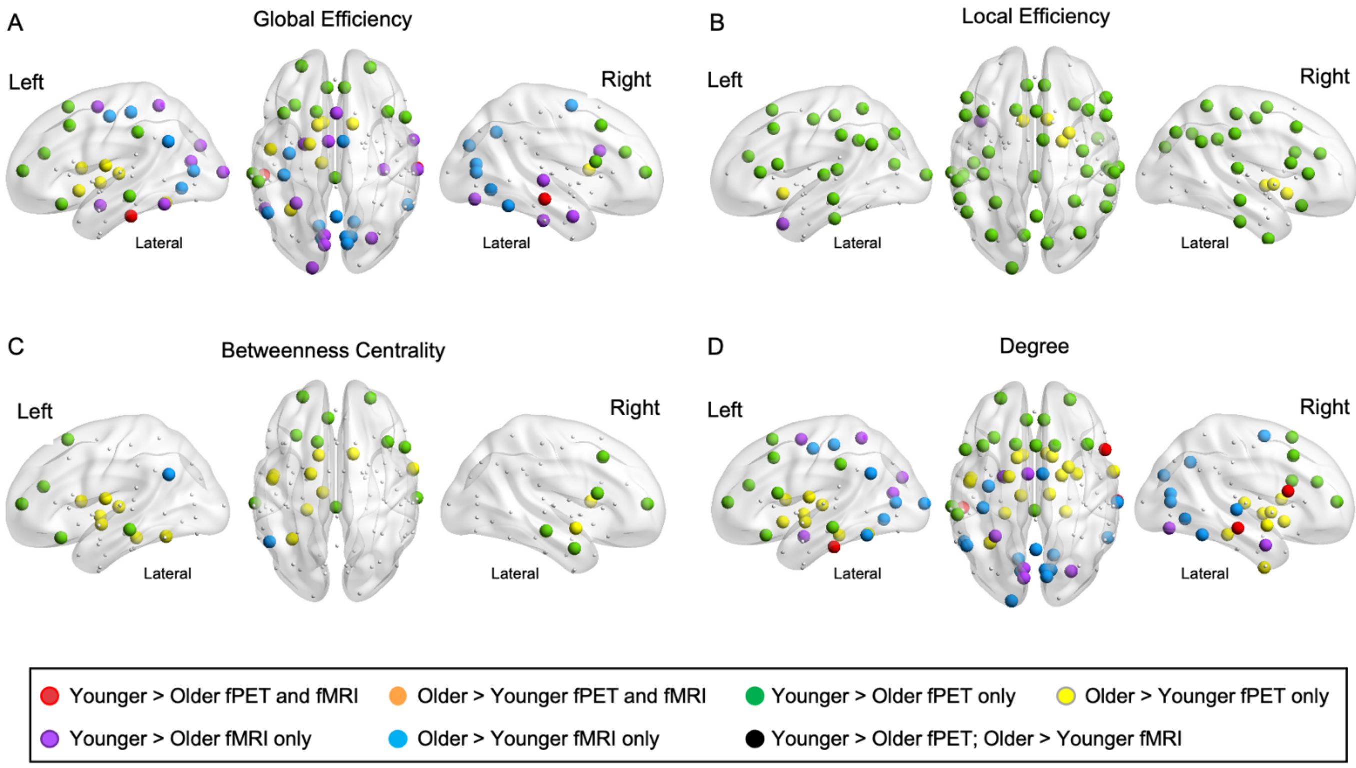

Supplementary Figure 4. fPET and fMRI age group differences in graph metrics for regions of the Harvard Oxford Atlas. Regions are shown as colored dots where there is a statistically significant difference at p-FDR < .05 for younger (N = 40) vs older (N = 46) adults from one-sided *t*-tests. For each region, the mean, standard deviations, effect sizes and t-tests are shown in Supplementary Tables 7 to 10. FR = frontal; LIM = limbic; SM = motor; PAR = parietal; MEDTP = medial temporal; SUB = subcortical; TEMP = temporal; OCC = occipital; MED = medial. Global efficiency (A) is average of the shortest inverse-distances between the node and all other nodes in the graph; local efficiency (B) is the average of shortest inverse-distances between the nodes within the neighbouring sub-graph; betweenness centrality (C) is the proportion of times that a node is part of a shortest-path between any two nodes within the graph; and degree (D) at each node defined as the number of edges from and to that node.

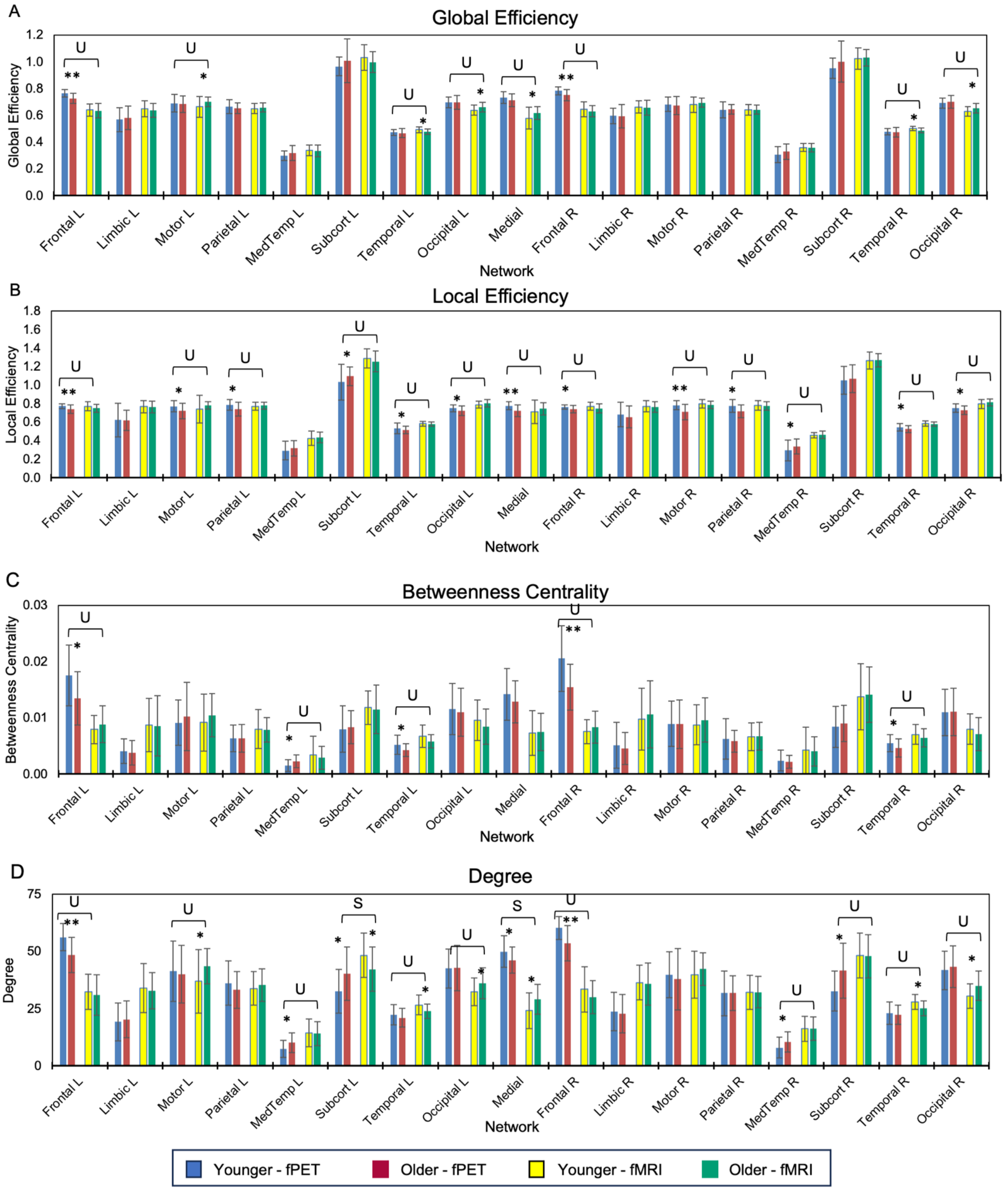

Supplementary Figure 5. Mean and standard deviation (error bars) of fPET and fMRI graph metrics for younger (N = 40) and older (N = 46) adults from the Harvard Oxford Atlas. Global efficiency (A) is average of the shortest inverse-distances between the node and all other nodes in the graph; local efficiency (B) is the average of shortest inverse-distances between the nodes within the neighbouring sub-graph; betweenness centrality (C) is the proportion of times that a node is part of a shortest-path between any two nodes within the graph; and degree (D) at each node defined as the number of edges from and to that node. Differences at *P-FDR < .05 and **p-FDR < .001 of younger (N = 40) vs older (N = 46) adults from one-sided *t*-tests; S = Age group differences, same direction in both modalities; U = unique age group differences in one modality; D = different age group direction in one modality vs the other.

Relative Predictive Strength of fPET and fMRI Graph Metrics for Age Group

The discriminant function analysis was significant for the fPET whole brain metrics (Wilk's Lambda = .837, Chi-square = 14.7, p < .002) but not the fMRI whole brain graph metrics (Wilk's Lamda = .955, Chi-square = 3.8, p = .285). The probability of a correct age group classification was 57% and 59% for younger and older adults in the fPET, respectively; and 52% and 52% for younger and older adults in the fMRI.

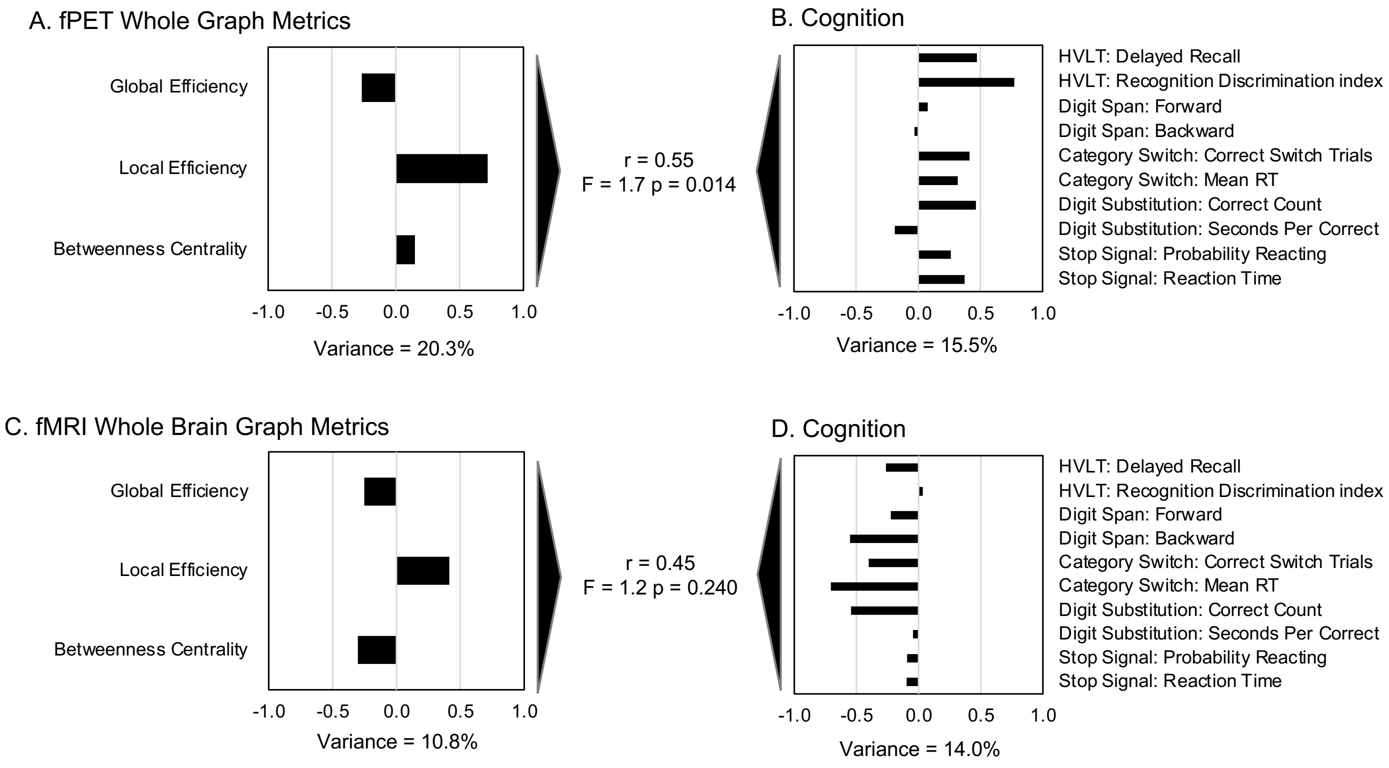

Supplementary Figure 6. Canonical correlations between the whole brain graph metrics from the Harvard Oxford Atlas for fPET (A) and fMRI (C) and cognition ((B) and (D)) in the whole sample (N = 86). r-value is the canonical correlation between the linear combinations of the graph metrics and cognition variables that maximally covary across subjects. F-statistic is Wilk's test of the null hypothesis that the canonical correlation and all smaller ones are equal to zero and was significant for one canonical variate for fPET but not fMRI. The correlations on each variable set represent the strength of the association between the variable and the canonical variate. Variance explained is the percentage of variance explained by the variables in their variate. Stop signal reaction time, seconds per correct response in the digit substitution and category switch reaction time were multiplied by -1 so that higher scores reflect better performance. The test of difference between two correlations = 0.86; p = .389.

Supplementary References

1. Jamadar SD, Ward PGD, Close TG, et al. Simultaneous BOLD-fMRI and constant infusion FDG-PET data of the resting human brain. *Sci Data*. Oct 21 2020;7(1):363. doi:10.1038/s41597-020-00699-5
2. Greve DN, Salat DH, Bowen SL, et al. Different partial volume correction methods lead to different conclusions: An (18)F-FDG-PET study of aging. *Neuroimage*. May 15 2016;132:334-343. doi:10.1016/j.neuroimage.2016.02.042
3. Greve DN, Svarer C, Fisher PM, et al. Cortical surface-based analysis reduces bias and variance in kinetic modeling of brain PET data. *Neuroimage*. May 15 2014;92:225-36. doi:10.1016/j.neuroimage.2013.12.021
4. Schaefer A, Kong R, Gordon EM, et al. Local-Global Parcellation of the Human Cerebral Cortex from Intrinsic Functional Connectivity MRI. *Cereb Cortex*. Sep 1 2018;28(9):3095-3114. doi:10.1093/cercor/bhx179
5. Eickhoff SB, Constable RT, Yeo BTT. Topographic organization of the cerebral cortex and brain cartography. *Neuroimage*. Apr 15 2018;170:332-347. doi:10.1016/j.neuroimage.2017.02.018
6. Di X, Biswal BB, Alzheimer's Disease Neuroimaging I. Metabolic brain covariant networks as revealed by FDG-PET with reference to resting-state fMRI networks. *Brain Connect*. 2012;2(5):275-83. doi:10.1089/brain.2012.0086
7. Savio A, Funger S, Tahmasian M, et al. Resting-State Networks as Simultaneously Measured with Functional MRI and PET. *J Nucl Med*. Aug 2017;58(8):1314-1317. doi:10.2967/jnumed.116.185835
8. Lodge MA, Rahmim A, Wahl RL. Simultaneous measurement of noise and spatial resolution in PET phantom images. *Phys Med Biol*. Feb 21 2010;55(4):1069-81. doi:10.1088/0031-9155/55/4/011
9. Deery HA, Di Paolo R, Moran C, Egan GF, Jamadar SD. Lower brain glucose metabolism in normal ageing is predominantly frontal and temporal: A systematic review and pooled effect size and activation likelihood estimates meta-analyses. *Hum Brain Mapp*. Feb 15 2023;44(3):1251-1277. doi:10.1002/hbm.26119
10. Deery HA, Liang, E., Siddiqui,M. N., Murray, G., Voigt, K., Di Paolo, R., Moran, C., Egan, G.F., and Jamadar, S.D. Metabolic Connectvity in Ageing. 2024
